# Loss-engineered reconfigurable polaritons

**DOI:** 10.1038/s41467-026-77412-4

**Published:** 2026-09-09

**Authors:** Hongyi Yuan, Kaijun Feng, Oubo You, Yihan Liao, Yongxin Lan, Shenghan Zhou, Shu Zhang, Yuchuan Xiao, Deng Pan, Zhiwen Shi, Cheng-Wei Qiu, Xiangdong Guo, Qing Dai

**Affiliations:** 1 Institute of Information Functional Materials, Shanghai Key Laboratory of Atomic-Level Intelligent Manufacturing of Materials and Devices, School of Materials Science and Engineering, Shanghai Jiao Tong University, Shanghai, China; 2 Key Laboratory for Micro-Nano Physics and Technology of Hunan Province, State Key Laboratory of Chemo/Biosensing and Chemometrics, College of Materials Science and Engineering, Hunan University, Changsha, Hunan China; 3 CAS Key Laboratory of Nanophotonic Materials and Devices, CAS Key Laboratory of Standardization and Measurement for Nanotechnology, National Center for Nanoscience and Technology, Beijing, China; 4 Suzhou Laboratory, Suzhou, China; 5 State Key Laboratory of Precision Spectroscopy, East China Normal University, Shanghai, China; 6 Key Laboratory of Artificial Structures and Quantum Control (Ministry of Education), School of Physics and Astronomy and Tsung-Dao Lee Institute, Shanghai Jiao Tong University, Shanghai, China; 7 Department of Electrical and Computer Engineering, National University of Singapore, Singapore, Singapore

**Keywords:** Polaritons, Nanophotonics and plasmonics, Two-dimensional materials

## Abstract

Phonon polaritons, with their strong field confinement, enable extreme miniaturization of photonic devices. While optical loss is conventionally perceived as a fundamental limit to be compensated, here we demonstrate a novel scheme by harnessing leakage-induced loss as a degree of freedom for mode manipulation. By coupling 2D hyperbolic polaritons (e.g., α-MoO_3_) with 3D bulk leaky channels (e.g., β-Ga_2_O_3_), we achieve a topological reconstruction into quasi-one-dimensional (quasi-1D) polaritons over a broad frequency range. In the frequency domain, out-of-plane phonon-driven leakage in the substrate potently controls the propagation direction of quasi-1D polaritons, enabling continuous frequency-dependent rotation and culminating in an abrupt transition at the phonon resonance. In real space, the quasi-1D isofrequency contours enable on-chip reflectionless propagation across wide angles (>170°) due to their extreme momentum-space collimation. This approach is further generalized to other 2D/3D heterostructures (e.g., α-MoO_3_/CdWO_4_), demonstrating operability across various discrete infrared spectral windows (total Δλ ~ 7 μm). Our findings not only introduce a loss-engineering framework for precise polariton control but also open new possibilities for zero-reflection on-chip optical routing and advanced optical information processing.

## Abstract (web summary)

Here, the authors show that, by harnessing angle-selective substrate leakage, hyperbolic phonon polaritons can be topologically reconstructed into quasi-one-dimensional modes with frequency-tunable directional propagation.

## Introduction

As hybrid quasiparticles of photons and lattice vibrations, phonon polaritons enable the confinement and manipulation of light at deep-subwavelength scales, positioning them as key building blocks for on-chip photonics. Progressive lowering of crystal symmetry breaks modal symmetry, evolving their isofrequency contours (IFCs) from circular to hyperbolic and hyperbolic-shear shapes^1–5^. Accordingly, the materials toolkit is continuously advancing into low-symmetry systems, with its scope further expanding from two-dimensional (2D) layered materials to bulk crystals^1,5^, including h-BN (hexagonal)^6–9^, CaCO_3_ (trigonal)^10,11^, YVO_4_ (tetragonal)^12^, α-MoO_3_ (orthorhombic)^13,14^, and β-Ga_2_O_3_ (monoclinic)^15–17^. These developments lay the groundwork for next-generation integrated optical information processing^18,19^.

While reduced crystal symmetry broadens the available polariton types and operating bands, performance in functional device designs has long been limited by optical losses^1–3^, which are considered as the principal bottleneck hindering the practical realization of phonon polariton-based devices. Generally, the optical loss of phonon polaritons propagating in high-quality thin films comprises two categories: intrinsic material loss (mainly phonon absorption^20^) and extrinsic loss^21^. The latter mainly consists of two components: scattering loss from radiation of polariton energy into the ambient, and leakage loss from radiative coupling into the substrate. Strategies to mitigate loss have evolved from passive avoidance (e.g., isotope engineering^22–24^) to active compensation (e.g., synthetic complex frequency waves^25^). However, these approaches generally treat loss as a defect to be erased.

Here, we propose a loss-engineering strategy that exploits out-of-plane phonon resonance to topologically reconfigure the substrate’s leaky channels, effectively pruning 2D hyperbolic modes into quasi-1D rays instead of suppressing leakage. Specifically, we achieve precise alignment between hyperbolic waveguide modes in a 2D overlayer (e.g., α-MoO_3_) and bulk leaky channels within a three-dimensional (3D) substrate (e.g., β-Ga_2_O_3_). By leveraging leaky loss in β-Ga_2_O_3_ as a control mechanism, we effectively reconfigure α-MoO_3_ hyperbolic waveguide modes into quasi-1D polaritons. Furthermore, out-of-plane phonon-induced leakage in the substrate directs the propagation direction of quasi-1D polaritons, causing a continuous frequency-dependent rotation that culminates in an abrupt transition at the phonon resonance frequency. In real space, the unique isofrequency contours of these quasi-1D polaritons enable reflectionless propagation across interfaces at ultra-wide angles (>170°), a property directly applicable to the design of deep-subwavelength polaritonic rainbow routers. More importantly, this mechanism exhibits excellent universality, making it extendable to a wide range of heterostructures composed of 2D van der Waals (vdW) materials and 3D monoclinic crystals (e.g., α-MoO_3_/CdWO_4_) and enabling operation across an ultra-broad infrared band (Δλ ~ 7 μm). This work not only introduces a novel approach to precisely manipulating phonon polaritons via loss-engineering, but it also provides a possible platform for fast on-chip information processing devices.

## Results

### Reconfiguring polariton mechanisms via leaky loss

**Fig. 1:**
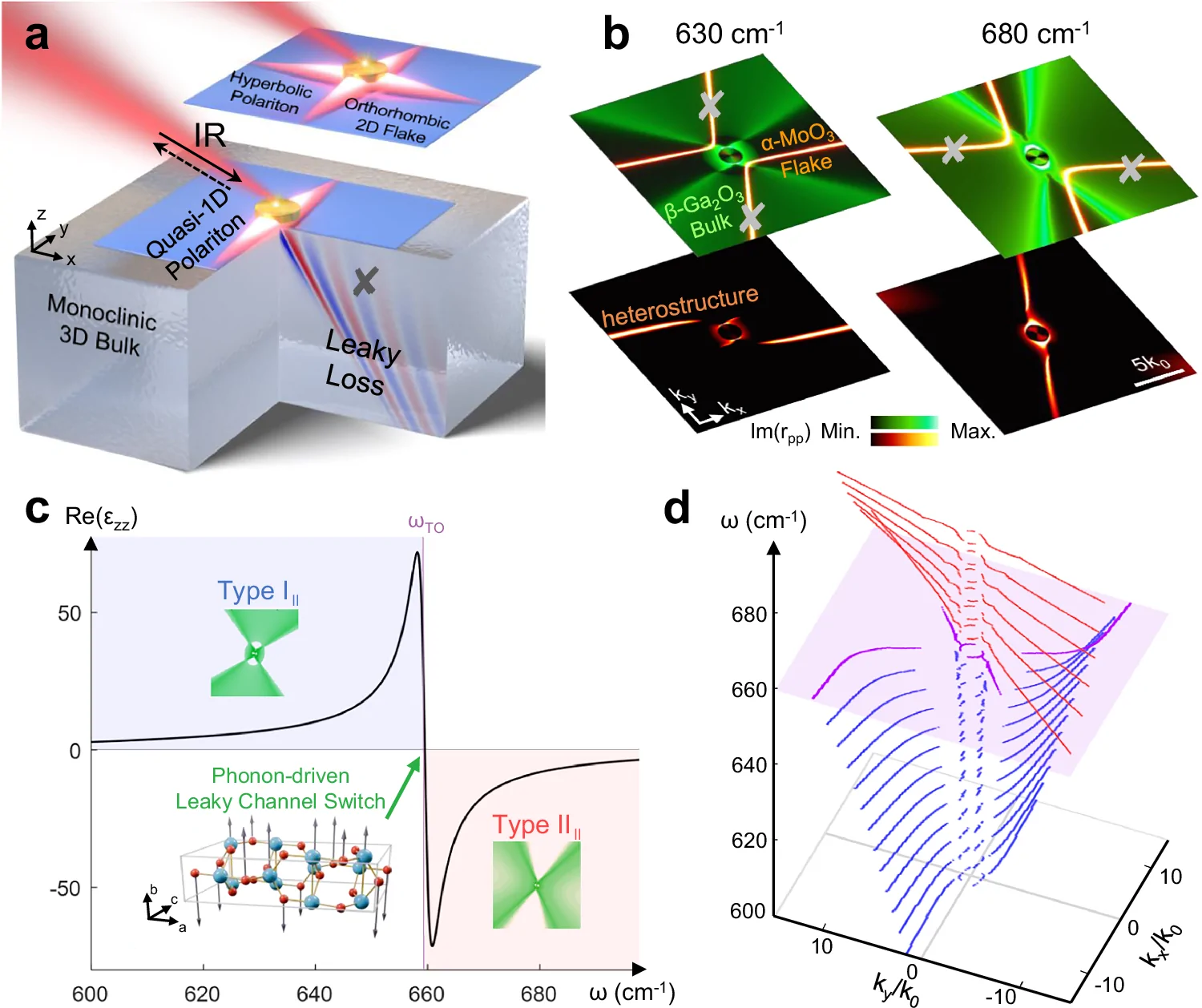
The mechanism of polariton tailoring and switching by out-of-plane phonons. **a** Schematic of quasi-1D polariton. The top panel shows the freestanding 2D flake’s hyperbolic polariton, while the 3D bulk’s leaky loss transforms it into quasi-1D polariton. **b** The formation mechanism of quasi-1D polaritons. Top panel: IFCs of the isolated α-MoO_3_ 2D flake (orange) and β-Ga_2_O_3_ 3D bulk (green) with twist angle 26°. Bottom panel: IFCs of their heterostructure. The light gray “×” marks indicate that a part of the hyperbola has been suppressed due to leaky loss into the substrate (corresponding to the dark gray “×” marks in **a**). **c** Switching of leaky channels caused by out-of-plane phonons in β-Ga_2_O_3_. There is a sign reversal of Re(ε_zz_) at the out-of-plane phonon frequency (~659 cm^–1^), resulting in a change of types of its in-plane hyperbolic modes. The illustrations are a rendering of the corresponding TO phonon mode in β-Ga_2_O_3_ with A_u_ symmetry, and IFCs of β-Ga_2_O_3_ of 655 cm^−1^ and 665 cm^−1^. **d** Evolution of the IFC for quasi−1D polariton in the α-MoO_3_/β-Ga_2_O_3_ heterostructure. On either side of 659 cm^−1^, the mode rotates clockwise and anticlockwise as frequency increases, respectively.

Loss engineering refers to leveraging optical losses to tailor the isofrequency contours, near-field distributions, and mode characteristics of polaritons, thereby turning loss into an effective tool for polariton modulation. Building upon this concept, we introduce a phonon-switch leaky loss mechanism that sculpts hyperbolic waveguide modes into switchable quasi-1D polaritons (see Supplementary Note 1 and Fig. S1 for its definition). To explain the proposed phonon-switch leaky loss mechanism, Fig. 1a presents two perpendicular cross-sections illustrating the differences in propagation directions of the hyperbolic polariton waveguide mode. In the leaky direction (cross-marked), polaritons leak into the 3D substrate and decay out-of-plane; in the non-leaky direction, they propagate as confined in-plane 1D modes. For the design of materials with practical application potential, we primarily consider orthorhombic 2D materials, such as α-MoO_3_, to realize hyperbolic waveguide modes. Meanwhile, for the 3D substrate serving as the leaky channel, we select monoclinic β-Ga_2_O_3_ with its intrinsic bulk properties. Specifically, from the perspective of momentum-space topological reconstruction mechanisms, Fig. 1b compares the evolution of the IFCs before and after forming a heterostructure composed of a 200 nm-thick α-MoO_3_ flake on a semi-infinite β-Ga_2_O_3_ bulk substrate (twist angle θ = 26°). The top row shows the IFCs of the isolated components in distinct colors; the green-shaded region represents continuous hyperbolic bulk modes which serve as leaky channels^26^, instead of previously reported hyperbolic surface shear mode^15,17^. The bottom row displays the IFC of their heterostructure: the substrate’s leaky region acts as a momentum-space filter, effectively ‘pruning’ parts of the hyperbolic branches. The surviving modes condense into highly directional modes, exhibiting quasi-1D diffraction-free propagation analogous to asymptotic line polaritons^27^. In addition, by simulating the real-space optical field distribution in the heterostructure, the process of hyperbolic waveguide polariton modes leaking into the substrate is further visually demonstrated. The extreme in-plane collimation overcomes the inherent spreading of conventional surface waves, enabling robust directional energy transport (details in the Supplementary Notes 2-4 and Fig. S2). Additionally, our analysis reveals that reducing the intrinsic loss of the substrate significantly enhances the downward leakage from the cladding layer, effectively facilitating the in-plane generation of quasi-1D polaritons by optimizing energy leakage transmission efficiency (Supplementary Notes 5 and 6; Fig. S3-5).

Critically, the propagation direction of quasi-1D polaritons is topologically reconfigured by the substrate’s out-of-plane transverse optical (TO) phonons (~659 cm^–1^). In Fig. 1b, distinct hyperbolic mode branches of α-MoO_3_ are selectively preserved at 630 cm⁻¹ and 680 cm⁻¹, due to a sign reversal in the real part of the out-of-plane dielectric function (Re(ε_zz_)) induced by out-of-plane TO phonons in the low-symmetry β-Ga_2_O_3_ substrate (Fig. 1c). This reconfigurability is driven by a phonon-induced topological transition in the substrate’s IFCs from type I_∥_ to type II_∥_^26^ (Supplementary Note 7 and Fig. S6). Consequently, the topological transition switches the leaky channel and finally redirects the quasi-1D polaritons (Fig. S7).

In addition to the discrete switching mediated by out-of-plane phonons, the opening angle of the in-plane hyperbolic modes in 2D vdW materials varies continuously with frequency due to dispersion characteristics, thereby inducing a smooth rotation of the quasi-1D polaritons. Together, these two phonon-mediated mechanisms yield uniquely reconfigurable, frequency-dependent propagation. Figure 1d shows the evolution of the IFC (extracted from Im(r_pp_)) of the heterostructure in Fig. 1b: as the frequency increases from 600 cm^–1^ to 659 cm^–1^, the mode rotates clockwise; beyond 659 cm^–1^, it rotates anticlockwise up to 690 cm^–1^ (Fig. S8). There is a discontinuous jump at the transition frequency of 659 cm⁻¹, where the polaritons exhibit a linear crossing-like dispersion characteristic (Fig. S8a). The rotation direction can also be reversed by changing the twist angle θ in the same frequency range, through which the different parts of branches are selected (Supplementary Note 8, Fig. S9a).

For highly directional quasi-1D polaritons, researchers have proposed several alternative approaches, such as twisted heterostructure^16,28,29^, fabricated nanostructures^30^, and changing the polarization direction of the excitation source^31^. However, to date, an effective method for achieving in-situ, broadband modulation of the directional propagation of such quasi-1D polaritons is still lacking. Here, our proposed loss-engineering strategy enables precise control over polariton modes by leveraging leaky loss, which is a novel mechanism physically distinct from ordinary polariton hybridization in heterostructures among several flakes^28,29,32–34^. Notably, beyond controlling hyperbolic modes, the same leaky channel mechanism can be further extended to transform in-plane isotropic modes into bracket-like or channelized modes (Fig. S10).

### Direct experimental observation of quasi-1D polaritons

**Fig. 2:**
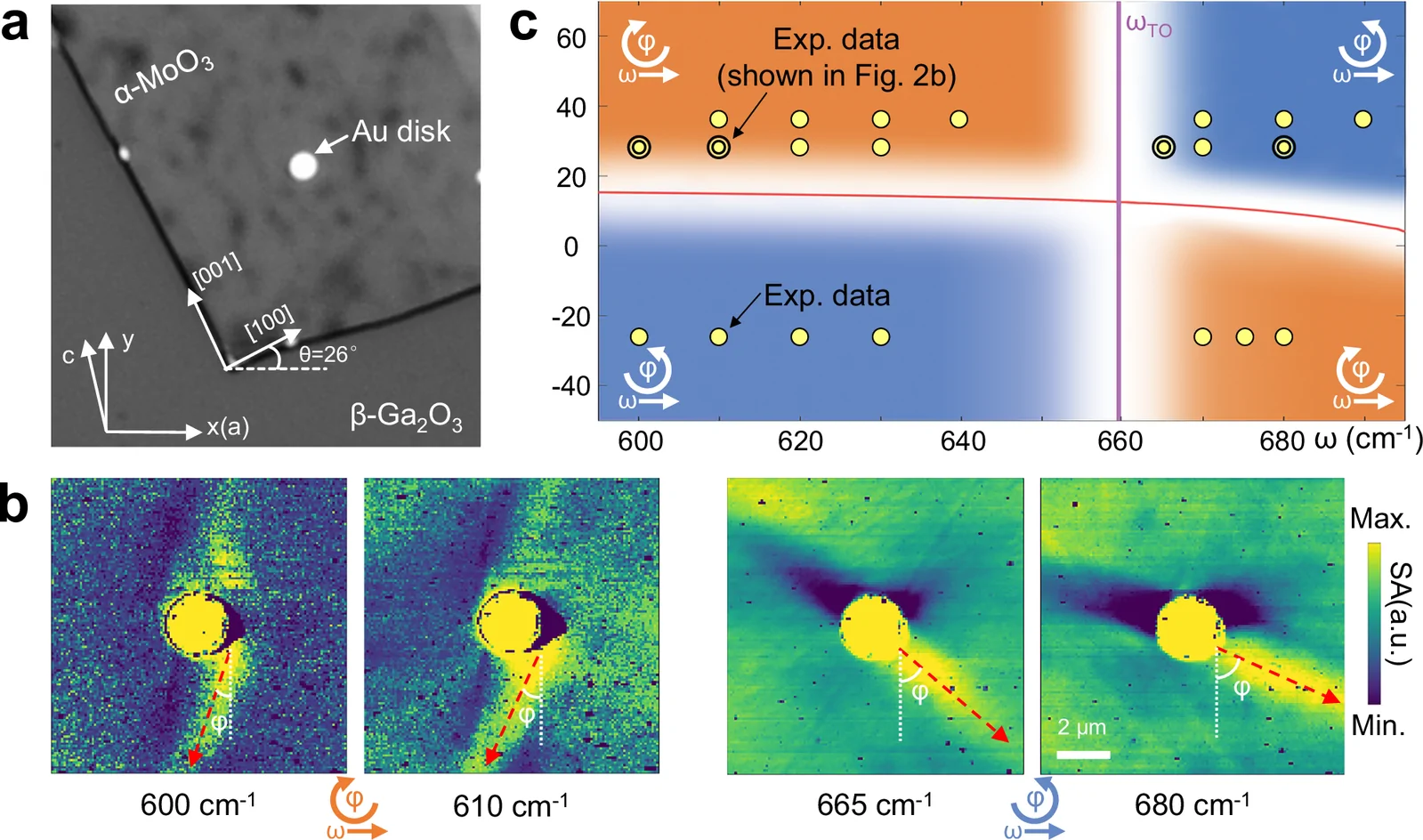
Direct near-field optical observation of quasi-1D polaritons. **a** Microscopic image of the measured α-MoO_3_ flake/β-Ga_2_O_3_ bulk heterostructure with a twist angle θ of 26°, featuring a central gold disk as the excitation source. **b** Near-field optical amplitude distribution illustrating the frequency-dependent rotation of the quasi-1D polariton propagation direction. The icon indicates the rotation direction of the propagation direction of polaritons as the frequency increases. The α-MoO_3_ flake thickness is 100 nm. The monoclinic angle of β-Ga_2_O_3_ between a-axis and c-axis is 103.7°. The diameter of the gold disk is ~2.5 μm. **c** Rotation direction of the quasi-1D polaritons’ propagation along the frequency increase. At a given twist angle, orange represents rotating clockwise and blue represents anticlockwise. The yellow dots represent the experimental verification data points, with those highlighted by circles shown in (**b**). The red solid line represents the rotation angle of in-plane hyperbola axis of β-Ga_2_O_3_ with changing frequency. The purple solid line represents the frequency of a β-Ga_2_O_3_’s out-of-plane TO phonon.

Building on the aforementioned theoretical design, we experimentally validate the mechanism of leveraging leaky loss as a regulatory tool (“scissors”) to tailor modes, thereby enabling precise control over quasi-1D polaritons. The in-plane hyperbolic mode’s opening angle of β-Ga_2_O_3_ offers a broad angular range for leaky channels, enabling quasi-1D polaritons to form across a wide range of twist angles. As a representative experimental realization within this regime, we fabricated and measured a heterostructure sample with a twist angle of 26° (Fig. 2a). Additional samples with other twist angles yield consistent results (Fig. S11). Because of the large mismatch between the free-space and polariton momenta, measuring the signal in far-field is challenging. We employ the scattering-type scanning near-field optical microscopy (s-SNOM) to measure the near-field optical signal (see Methods)^35,36^.

The near-field amplitude for different excitation frequencies across the phonon frequency is shown in Fig. 2b. By comparing the results of two bands, there is an obvious transition of both the mode propagation direction and frequency-dependent rotation direction before and after the TO phonon vibration frequency of β-Ga_2_O_3_ (~659 cm^–1^). A red dashed guideline is plotted to mark the quasi-1D polariton. To clearly show the rotation feature of the measured signal, an angle φ is defined as the angle between the guideline and the vertical reference line. Figure 2b shows that when frequency increases from 600 cm^–1^ to 610 cm^–1^, the quasi−1D polariton rotates clockwise. When frequency increases from 665 cm^–1^ to 680 cm^–1^, the quasi-1D polariton rotates anticlockwise. From the aspects of propagation feature and the contrast between the signal to background, the mode in the high frequency band is more intense than the one measured in low frequency band. This is a result of the difference in the mode type between two frequency bands. In the higher frequency band, the negative sign of the Re(ε_zz_) of β-Ga_2_O_3_ leads to the high reflectivity, which greatly compresses the mode and transforms the mode distribution into image phonon polariton^37^, without obvious decrease of quality factor. If the intrinsic loss of β-Ga_2_O_3_ substrate can be reduced, the pruning efficiency can be improved (Supplementary Note 9 and Fig. S12) and the quality factor of the mode can be even enhanced to surpass the original mode in freestanding α-MoO_3_ (Supplementary Note 10 and Fig. S13). The analysis of quasi-1D polariton ray-like (diffraction-free) patterns^10^, which extend over a distance up to 11 μm, is presented in Fig. S14 and Fig. S15. Through finite element method, simulation is conducted for the measured case, using the measured thickness of the overlayer (Supplementary Note 11, Fig. S16 and Fig. S17), which is consistent with the experiment results.

Figure 2c combines theoretical calculations and experimental filtered data to verify and summarize the rotational characteristics of quasi-1D polaritons as their frequency increases under specific twist angles. Theoretical calculations indicate that the orange and blue regions correspond to clockwise and anticlockwise rotation, respectively. Experimental data are represented by yellow dots (see experimental results in Fig. S11). Additionally, the data in Fig. 2b are highlighted with yellow dots encircled for emphasis. Further studies demonstrate that the propagation characteristics of these quasi-1D polaritons are robust against variations in both the incident direction of the excitation light (Supplementary Note 12 and Fig. S18) and the α-MoO_3_ flake thickness (Fig. S19).

### Wide-angle zero reflection of quasi-1D polaritons

**Fig. 3:**
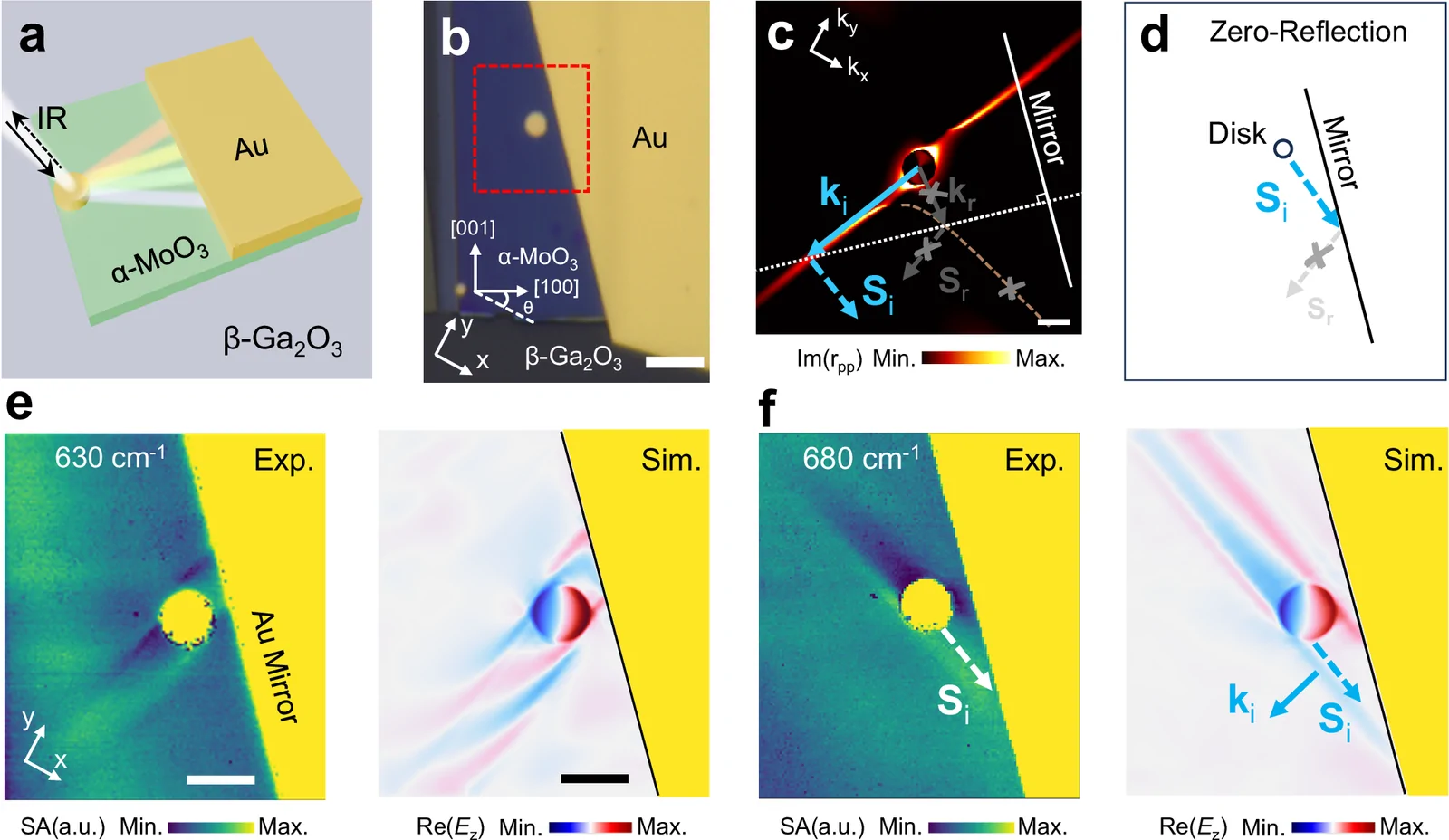
Zero reflection of quasi-1D polaritons. **a** Schematic of wide-band zero-reflection of quasi-1D polaritons whose frequencies are indicated by different colors. **b** Microscopic image of the measured sample. The twist angle is 26°. A single crystal gold plate is used as a mirror. The thickness of the α-MoO_3_ is 120 nm. The scale bar is 5 μm. **c** The theoretical analysis of zero reflection. The blue solid and dashed arrows show the incident wave vector and corresponding Poynting vector. The gray solid and dashed vectors show the reflected wavevector and Poynting vector that would occur if it was a hyperbolic situation (brown dashed curve), which are eliminated by the leakage mechanism. Subscripts “i” and “r” stand for incidence and reflection. The scale bar is 2*k*_0_. **d** Simplified real space schematic of zero-reflection of the case in (**c**). The experimental (left) and simulation (right) results of zero-reflection of quasi-1D polaritons of the heterostructure (measured in the red dashed box in **b**) for excitation frequency of 630 cm^–1^ (**e**) and 680 cm^–1^ (**f**). The quasi-1D polaritons propagate towards the boundary with no reflection, which is verified by the simulation results. The scale bar is 2 μm.

Leveraging the extreme asymmetry of the unique isofrequency contours of quasi-1D polaritons enables remarkable optical phenomena, such as broadband and wide-angle on-chip reflectionless propagation. Figure 3a shows the schematic figure of zero reflection in the heterostructure of α-MoO_3_/β-Ga_2_O_3_. Here, “zero reflection” specifically refers to the absence of a phase-matched (or momentum-matched) propagating reflected mode, rather than the complete absence of evanescent or weakly scattered fields. To experimentally investigate this effect, a single-crystalline gold flake is placed on the heterostructure, with its highly reflective edge serving as a mirror boundary. A gold disk launches quasi-1D polaritons towards this boundary, and an optical micrograph of the fabricated structure is shown in Fig. 3b.

In conventional hyperbolic polaritons, zero reflection occurs only when the interface is precisely aligned perpendicular to the hyperbolic asymptotes^38–40^. As a result, at any given incident angle, there exists only a single frequency that satisfies the zero-reflection condition—severely limiting its practical applicability. In contrast, quasi-1D polaritons enable zero reflection within the propagation plane, across multiple angles and frequencies, owing to their pronounced symmetry-breaking characteristics with highly directional propagating mode. To reveal the mechanism of the zero reflection of quasi-1D polariton, Fig. 3c provides a **k**-space analysis of the zero-reflection condition at 680 cm^–1^, while Fig. 3d illustrates the corresponding simplified real-space picture of reflectionless propagation. Based on the phase-matching requirement, specifically the continuity of the tangential component of the wavevector across the interface, we construct the incident wavevector **k**_i_ and its associated Poynting vector **S**_i_. Under these conditions, no physically valid reflected wavevector exists, marked with the gray arrows. Crucially, this behavior is no longer confined to a single asymptote-aligned angle. Instead, zero reflection can be achieved over an exceptionally wide angular range—over a > 170° angular span out of the full hemispherical incidence range—as illustrated by the angle dependence between Poynting vector and the boundary in Fig. S20. The remaining <10° angular range exhibits reversed reflection, where the reflected wave propagates at the same angle as the incident wave.

The wideband zero reflection is verified in experiment with s-SNOM using the sample in Fig. 3b. The experimental results are shown in the left panels of Fig. 3e and f. Cases of 630 and 680 cm^–1^ are presented for demonstration. Simulations are performed using the measured geometry parameters and correspond well with the experiment results, shown in the right panels of Fig. 3e, f. No reflection is observed in the measured near field signal and simulation, and it should be noted that when quasi-1D polaritons arrive at the gold edge, their energy flow transmits into the α-MoO_3_ layer beneath the gold flake, which cannot be observed in the measured near-field image. For comparison, the reflection of hyperbolic polariton at the gold mirror is given in Fig. S21. The zero-reflection phenomenon of quasi-1D polariton is not limited to gold-mirror interfaces but is also observed at the natural boundaries of α-MoO_3_ (Fig. S11b). It is possible to construct a dual-band reflectionless frequency router based on the zero-reflection property combined with the unique rotation feature of quasi-1D polariton (Fig. S22). The unique routing mechanism is rooted in the extreme anisotropy of the materials and needs no complex nanofabrication, which has application potential in robust directional information processing.

### Universal heterostructure platform for quasi-1D polaritons

To broaden the frequency spectrum for the application of quasi-1D polaritons, we further investigated and confirmed the universality of this mechanism in material design. Theoretical calculations reveal that quasi-1D polaritons can be formed when the hyperbolic waveguide modes in the thin cladding layer overlap with the hyperbolic bulk modes serving as leaky channels in the bulk substrate. Accordingly, materials capable of supporting in-plane hyperbolic bulk polaritons across diverse crystal systems, such as monoclinic β-Ga_2_O_3_ or trigonal CaCO_3_, may all be regarded as viable candidates. Remarkably, the construction of quasi-1D polaritons is achievable even when the cladding and substrate are composed of the same material^16^. However, this mechanism is limited by the frequency response matching between the cladding and the substrate. If a frequency-matching material combination cannot be found, this mode cannot be realized. For example, when employing CaCO_3_ with hyperbolic bulk modes as the leakage substrate in its upper Reststrahlen band (1410-1550 cm^-1^), it is currently very difficult to find a suitable cladding material that both matches the frequency and supports hyperbolic waveguide modes.

**Fig. 4:**
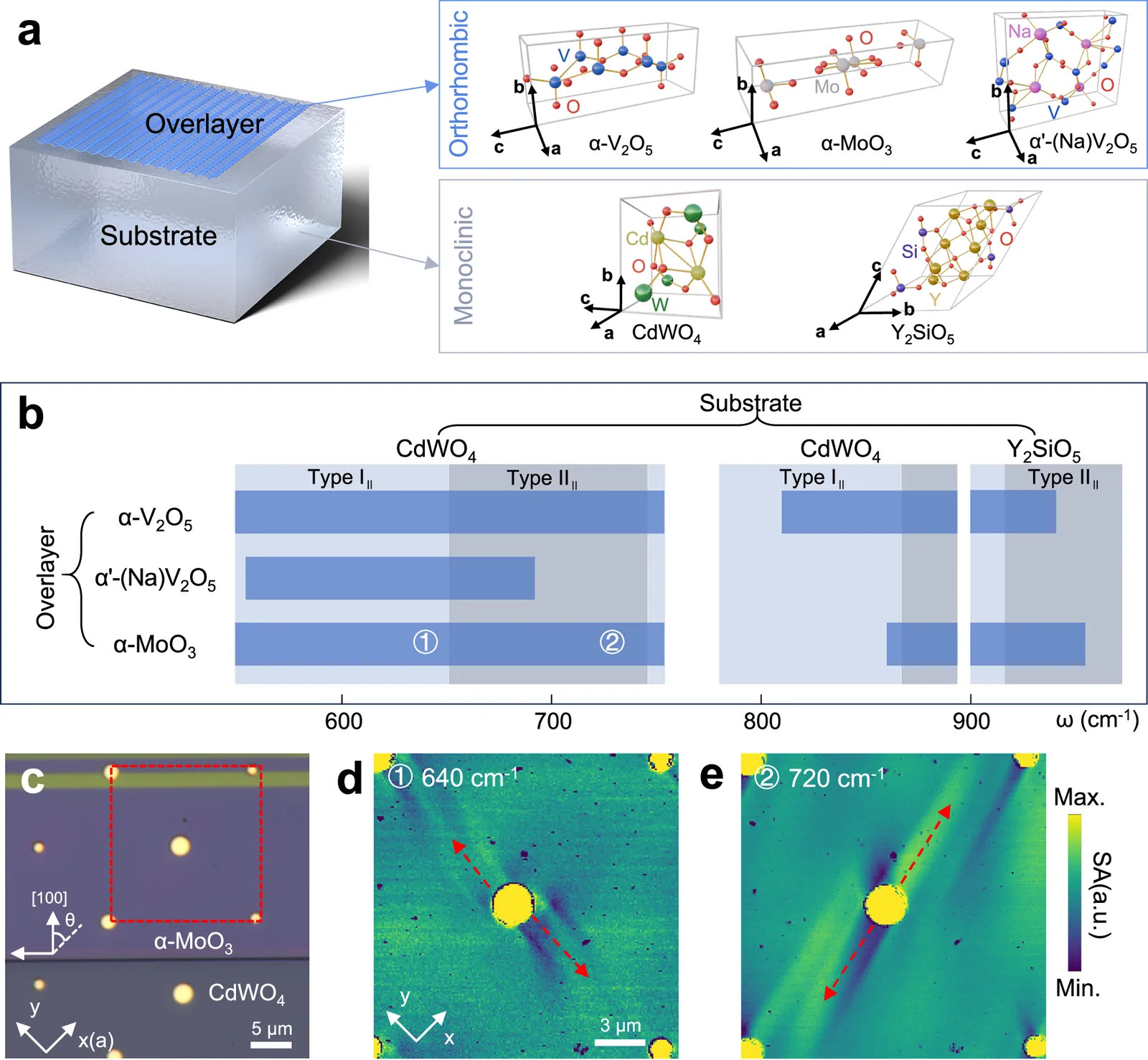
Broadband quasi-1D polaritons extended to heterostructures with different crystal combinations. **a** Schematic of the heterostructure (left) and the crystal structures involved (right). The first row lists the overlayer materials; the second row lists the substrate materials. Different kinds of atoms are marked with different colors. The arrows labeled **a**–**c** indicate the directions of the three basis vectors of the crystal lattice. **b** The in-plane hyperbolic polariton frequency range of the overlayer is compared with the substrate’s type‑I and type‑II in-plane hyperbolic bands (ice blue and light gray). When the overlayer and substrate bands overlap (blue), quasi-1D polaritons emerge; when the substrate’s band type switches, quasi−1D polaritons’ propagation direction switches. **c** Microscopic image of the measured 200-nm-thick α-MoO_3_ flake/CdWO_4_ bulk heterostructure, featuring a central gold disk as the excitation source. The twist angle θ between the [100] axis of these two kinds of material is 45°. **d**,** e** Near-field optical amplitude distribution illustrating the quasi-1D polariton of the heterostructure, corresponding to the cases ①, ② marked in (**b**).

Furthermore, controlling the propagation direction of quasi-1D polaritons requires tuning the frequency of the out-of-plane TO phonons in the substrate material, which places higher demands on substrate design. As shown in Fig. 4a, the examples of cladding materials include α-V_2_O_5_, α‘-(Na)V_2_O_5_^41^, and α-MoO_3_, while the substrate materials include CdWO_4_^42^ and Y_2_SiO_5_^43^. Notably, the substrates are primarily composed of monoclinic crystals, whose low-symmetry characteristics provide abundant out-of-plane TO phonons, facilitating frequency matching during the topological transition of leaky channels from type I_∥_ to type II_∥_.

Following the above design concept of expandable heterostructures, Fig. 4b presents representative examples of frequency band matching for these material pairs (i.e., the shaded overlapping regions), offering deeper insights into the frequency band characteristics of the heterostructures. This facilitates precise tuning of frequency band ranges and flexible manipulation of quasi-1D polariton properties. Additional examples of IFC can be found in Fig. S23. Therefore, this class of material systems, through their diverse heterostructure designs, can cover a relatively broad spectral window in the mid-infrared band—specifically 549–754 cm^–1^ and 815–958 cm^–1^ (corresponding to wavelengths λ of 13.3–18.2 μm and 10.4–12.3 μm, respectively). Furthermore, the overall spectral range covered by several representative material combinations is approximately 7 μm, offering potential for material design and selection within ultra-wideband applications. To verify the generality of our scheme, we used the α-MoO_3_/CdWO_4_ heterostructure as an example to verify the reconfigurable propagation characteristics of quasi-1D polaritons. The microscope image is shown in Fig. 4c. By designing an appropriate twist angle for the heterostructure, a clear ray-like propagation of quasi-1D polaritons is achieved, with frequencies located at 640 cm^–1^ (Fig. 4d), and 720 cm^–1^ (Fig. 4e), respectively. Looking ahead, by engineering heterostructures to precisely control the leaky channels, it is expected that a novel design paradigm can be established, paving the way for the development of high-performance polaritonic devices.

## Discussion

In summary, we establish loss engineering as a novel strategy to transform phonon-driven optical leakage from detrimental loss source into a powerful design tool for phonon polaritons, where out-of-plane optical phonons topologically reconfigure leaky channels. By coupling 2D flake’s hyperbolic waveguide modes to 3D substrate’s leaky channels, we achieve controlled generation and manipulation of quasi-1D polaritons over a broad frequency range. In frequency domain, experimental validation using α-MoO_3_/β-Ga_2_O_3_ heterostructures reveals frequency-dependent rotation and transition of propagation direction, governed by substrate’s out-of-plane phonon. In real space, broadband and wide-angle (>170°) zero-reflection is verified in experiment. Crucially, our mechanism overcomes material-specific limitations, demonstrating universal applicability across a wide range of 2D/3D heterostructures (e.g., α-MoO_3_/CdWO_4_). Therefore, our work redefines the role of loss in polaritonic devices by converting it from a fundamental bottleneck into a functional asset, thereby opening new avenues for developing zero-reflection on-chip polaritonic routing devices and advanced information processors.

## Methods

### Nanofabrication of the samples

The α-MoO_3_ is mechanically exfoliated from a high-quality bulk crystal and then transferred onto a β-Ga_2_O_3_ substrate using a standard dry-transfer technique. Specifically, α-MoO_3_ flakes are picked up and stacked onto the β-Ga_2_O_3_ substrate with a hemispherical polydimethylsiloxane (PDMS) stamp coated in pure water. The twist angle is precisely controlled using a rotary stage with an accuracy of 0.1°. The twist angle of the heterostructure is defined as the anticlockwise rotation angle between the [100] axis of the upper α-MoO_3_ and the a-axis of the lower β-Ga_2_O_3_. Additionally, gold disks were patterned on the α-MoO₃ surface by electron-beam lithography, followed by electron-beam evaporation and lift-off.

### Synthesis of the single-crystalline gold

Single-crystalline gold nanoplates were synthesized on Si/SiO₂ substrates with a 285-nm oxide layer via a modified ethylene glycol reduction. A growth solution containing 1 mM HAuCl₄ and 0.2 vol% water in ethylene glycol was used. Substrates were sequentially sonicated in acetone and ethanol, rinsed with water, and treated with oxygen plasma for 15 min. Two substrates were immersed back-to-back in the solution at 90 °C for 12 h, exposing only one side each. After growth, the downward-facing substrate was retrieved to avoid particle sedimentation, rinsed with ethanol and water, and dried under nitrogen.

### Scanning near-field optical microscopy measurements

For near-field imaging, we utilized a commercial s-SNOM system (Neaspec) coupled with an atomic force microscope (AFM). A platinum-coated AFM probe was vertically oscillated at 270 kHz with a 50 nm amplitude. Illumination was provided by a tunable broadband optical parametric oscillator laser covering 550–4000 cm^–1^. Background suppression was achieved through higher harmonic demodulation (nΩ, n ≥ 2) of the interferometric signals, yielding simultaneous near-field amplitude and phase imaging.

### Full-wave numerical simulations

Numerical simulations were conducted using the finite element method (FEM) implemented in COMSOL Multiphysics 6.2. For near-field experimental simulations, we established a 3D model of the multilayer structure, incorporating a 100-nm-high dipole excitation source at the top surface. To better approximate experimental conditions, we included a gold disk at the structure’s apex, simulating illumination by far-field incident light at a 52° polar angle. Additionally, we employed COMSOL’s eigenmode analysis feature to develop a 2D model of the multilayer configuration for comprehensive investigation.

### Theoretical calculations

The calculations of the IFC are performed using a generalized 4 × 4 transfer matrix formalism. In short, by incorporating the permittivity of the overlayer and substrate, the solution of p-polarized reflection coefficient, *r*_*pp*_ (*q*, *ω*), can be determined. Polariton mode corresponds to the frequencies at which the imaginary parts of *r*_*pp*_(*q*, *ω*) reach the maximum. For a given frequency, the polariton wavevector in the k_x_-k_y_ plane can be extracted as IFC.

## Supplementary information

Supplementary Information

Transparent Peer Review file

## Data Availability

The data that support the findings of this study are available in the Article and the Supplementary Information. Other relevant data are available from the corresponding authors upon request.
