# Supplementary material for: Loss-engineered reconfigurable polaritons
Source: Nat Commun. 2026 Sep 9;17:10658. doi: 10.1038/s41467-026-77412-4 (PMC13654931; doi:10.1038/s41467-026-77412-4)
Supplement: Supplementary file 1 [file 41467_2026_77412_MOESM1_ESM.pdf]

# Supplementary Information for
# "Loss-Engineered Reconfigurable Polaritons"

Hongyi Yuan[1#], Kaijun Feng[1#], Oubo You[1], Yihan Liao[1,2], Yongxin Lan[1], Shenghan Zhou[1], Shu Zhang[3], Yuchuan Xiao[4], Deng Pan[5], Zhiwen Shi[6], Cheng-Wei Qiu[7], Xiangdong Guo[1*], and Qing Dai[1*]

[1] Institute of Information Functional Materials, Shanghai Key Laboratory of Atomic-Level Intelligent Manufacturing of Materials and Devices, School of Materials Science and Engineering, Shanghai Jiao Tong University, Shanghai, China.

[2] Key Laboratory for Micro-Nano Physics and Technology of Hunan Province, State Key Laboratory of Chemo/Biosensing and Chemometrics, College of Materials Science and Engineering, Hunan University, Changsha, Hunan 410082, China.

[3] CAS Key Laboratory of Nanophotonic Materials and Devices, CAS Key Laboratory of Standardization and Measurement for Nanotechnology, National Center for Nanoscience and Technology, Beijing, China.

[4] Suzhou Laboratory, Suzhou, China

[5] State Key Laboratory of Precision Spectroscopy, East China Normal University, Shanghai, China

[6] Key Laboratory of Artificial Structures and Quantum Control (Ministry of Education), School of Physics and Astronomy and Tsung-Dao Lee Institute, Shanghai Jiao Tong University, Shanghai, China

[7] Department of Electrical and Computer Engineering, National University of Singapore, Singapore, Singapore

[#] These authors contributed equally.

**emails*: guoxiangdong@sjtu.edu.cn, and daiqing@sjtu.edu.cn

**Contents:**

This PDF file includes:

Contents: .......... 2
Note 1: Definition of quasi-1D polaritons .......... 3
Note 2: The dielectric functions of related materials .......... 3
Note 3: Calculation of mode analysis .......... 4
Note 4: The leaking process of the hyperbolic mode of the overlayer .......... 4
Note 5: The role of leaky loss .......... 5
Note 6: The effects of loss in the leaky channels .......... 5
Note 7: Types of the hyperbolic isofrequency surface .......... 6
Note 8: IFC analysis of modulation of quasi-1D polaritons .......... 8
Note 9: Analysis of the pruning efficiency .......... 9
Note 10: The effects of loss on the propagation modes .......... 9
Note 11: Simulation of quasi-1D polariton .......... 10
Note 12: The effects of illumination orientation to quasi-1D polaritons .......... 11
Fig. S1: Analysis of real space distribution of quasi-1D polaritons. .......... 12
Fig. S2: Leaking process revealed with mode analysis .......... 13
Fig. S3: The influence of the substrate's thickness .......... 14
Fig. S4: Tuning the coupling strength with dielectric medium interlayer .......... 15
Fig. S5: Mode analysis results of mode in the leaking directions of two frequency bands .......... 16
Fig. S6: Types of in-plane hyperbolic modes of β-$Ga_2O_3$. .......... 17
Fig. S7: The switching mechanism of out-of-plane dielectric function .......... 18
Fig. S8: Quantitative analysis of frequency-dependent rotation of quasi-1D polaritons. .......... 19
Fig. S9: Frequency- and twist-dependent rotation reversal of quasi-1D polaritons .......... 20
Fig. S10: Tailoring of in-plane isotropic modes via the leaky channel mechanism .......... 21
Fig. S11: Experimental results of α-$MoO_3$/β-$Ga_2O_3$ heterostructure with other twist angles . 22
Fig. S12: Calculation of pruning efficiency .......... 23
Fig. S13: Mode analysis results of propagation modes in two frequency bands .......... 24
Fig. S14: Propagation analysis of quasi-1D polaritons generated by a point dipole. .......... 25
Fig. S15: Propagation analysis of quasi-1D polaritons generated by a gold disk .......... 26
Fig. S16: The simulation results for the case of 26° twist angle .......... 27
Fig. S17: AFM figure of the measured sample .......... 28
Fig. S18: The dependence on illumination orientation of quasi-1D polaritons. .......... 29
Fig. S19: Simulation of propagation with different flake thickness. .......... 30
Fig. S20: Interface angle dependence of zero reflection of quasi-1D polaritons .......... 31
Fig. S21: The near-field images of quasi-1D polaritons with gold mirror. .......... 32
Fig. S22: Dual-Band Polariton Rainbow Router .......... 33
Fig. S23: Iso-frequency contours of other material pairs supporting quasi-1D polaritons .......... 34
References: .......... 35

**Note 1: Definition of quasi-1D polaritons**

Here, we discuss the definition of the concept of quasi-one-dimensional (quasi-1D) polaritons from the mode distribution and physical mechanism.

First, from the perspectives of angular divergence and transverse confinement, we performed a quantitative analysis of the $|\mathbf{E}_z|$ field distribution under a representative configuration in Fig. S1 [1]. The concentric field pattern centered at the excitation source shows that, as the propagation distance increases, the peak angular position remains unchanged, whereas the angular full width at half maximum (FWHM) gradually decreases. Meanwhile, the transverse FWHM of the field profile along the propagation direction remains nearly constant. These results confirm that the mode exhibits both high collimation and strong transverse confinement.

Second, from the perspective of the underlying physical mechanism, namely the characteristics of the isofrequency contour, this behavior originates from the fact that the energy flow represented by the Poynting vector is nearly orthogonal to the wavevector across the entire field pattern. In other words, the group velocity is nearly orthogonal to the phase velocity, giving rise to highly directional, ray-like propagation.

In summary, based on this strongly collimated energy transport and the propagation symmetry along a single direction, we formally refer to this excitation mode as the "quasi-1D" polariton.

**Note 2: The dielectric functions of related materials**

The accurate characterization of dielectric functions is essential for predicting polariton behavior. In the mid-infrared region, the optical response of $\alpha$-$MoO_3$ is dominated by phonon absorption rather than electronic transitions. The dielectric function can be described by the Lorentz model[2]:

$$\varepsilon_j = \varepsilon_\infty^j \left( 1 + \frac{{\omega_{\mathrm{LO}}^j}^2 - {\omega_{\mathrm{TO}}^j}^2}{{\omega_{\mathrm{TO}}^j}^2 - \omega^2 - i\omega\Gamma^j} \right), \quad j = x, y, z$$

Here, $\varepsilon_j$ represents the principal components of the permittivity tensor, $\varepsilon_\infty^j$ is the high-frequency dielectric constant, $\omega_{\mathrm{LO}}^j$ and $\omega_{\mathrm{TO}}^j$ are the longitudinal and transverse optical phonon frequencies, respectively, and $\Gamma^j$ is the Lorentzian broadening factor. The crystal axes x, y, and z

correspond to the [100], [001], and [010] crystalline directions of α-$MoO_3$, respectively. The phonon frequencies used for calculating the relative permittivity are taken from references[3].

Similarly, the dielectric functions of β-$Ga_2O_3$[4], $CdWO_4$[5], $Y_2SiO_5$[6], α-$V_2O_5$, and α'-(Na)$V_2O_5$[7], refer to the corresponding references respectively.

**Note 3: Calculation of mode analysis**

The mode analysis is calculated using commercial finite element method software, COMSOL 6.2. To optimize computational efficiency without compromising accuracy, two-dimensional model is employed. The model plane is aligned along the wave vector whose modes are analyzed. The wave vector is determined according to the iso-frequency contour, including the angle and value. The structure is rotated to align the model plane with the wave vector. A numerical port is set on the left boundary and boundary mode analysis is added in the study step. We use mode solver to search modes around the value of normalized wave vector. The structure consists of 20-μm thick air, 200-nm thick α-$MoO_3$ and 40-μm thick β-$Ga_2O_3$. Perfectly matched layer is applied on the right side of the simulation area to absorb the surplus energy.

**Note 4: The leaking process of the hyperbolic mode of the overlayer**

To elucidate the modulation mechanism, Fig. S2 compares the two branches with and without the substrate's angular selective leakage (see method for details). The left panel depicts the IFCs of the hyperbolic waveguide mode of an isolated 2D overlayer and the leaky channel of an isolated 3D substrate, shown with different colors. To analyze the leakage process, the middle panel visualizes the cross-sectional polariton field distribution of the two branches, marked with ① & ② in the left panel. For branch ① within 3D substrate's bulk mode region (green area), the field distribution confirms a leakage-induced loss channel. Wave excited at the left port is guided into the bulk substrate, where it decays exponentially with a slanted wavefront. For branch ② out of the bulk mode region, the mode remains the common propagation for a 2D hyperbolic flake along the wave vector. The *z*-component of the electric field is concentrated at the surface and decays exponentially. Simulation of the real-space electric field distribution (Re($E_z$)), shown in the right panel, reveals that the

heterostructure supports highly directional, collimated energy flow along a 1D path. Compared to common hyperbolic modes, two of the four extension directions (corresponding to branch ①, marked with a grey dotted box) are suppressed because of leaky loss into the substrate, while the remaining two directions (corresponding to branch ②) form quasi-1D polaritons.

**Note 5: The role of leaky loss**

The loss-engineering strategy relies on wave-vector matching, which causes energy from highly compressed phonon polaritons in the capping layer to selectively leak into the substrate's continuum of modes—a process analogous to leakage into air for modes near the free-space light cone (see Fig. S3 for substrate thickness effects). As shown in Fig. S4, this downward leakage can be effectively suppressed by introducing a dielectric interlayer, further validating the mechanism. A clearer ray-like propagation of quasi-1D polaritons (that is, a straighter pattern in the IFC) can be achieved by selecting a twist angle that brings the uncanceled branch close to one of the hyperbolic asymptotes of the base material due to the influence of mode hybridization.

**Note 6: The effects of loss in the leaky channels**

To illustrate the effects of loss on the leaky channels, mode analysis is performed in two bands. The isofrequency contours of β-$Ga_2O_3$ bulk (green) and 200-nm thick α-$MoO_3$ flake (orange) are shown in Fig. S5a (630 $cm^{-1}$) and S5e (680 $cm^{-1}$). The z-component of the electric field of the heterostructure is denoted with the blue box in Fig. S5a. The mode profile in β-$Ga_2O_3$, calculated with the imaginary part of the dielectric function reduced to 10%, is shown in Fig. S5c. Fig. S5e and f (0.5 times the imaginary part of the dielectric function in β-$Ga_2O_3$) show the cases for 680 $cm^{-1}$, which is denoted with the blue box in Fig. S5d. The propagation direction of the leaked wave is different from the case of 630 $cm^{-1}$, which is the result of the sign reversal of the real part of the $\varepsilon_{zz}$. Therefore, the smaller the imaginary part of the dielectric function of the 3D substrate, the more the electromagnetic field leaks into it.

**Note 7: Types of the hyperbolic isofrequency surface**

In homogeneous, source-free, non-magnetic, and anisotropic media, where the dielectric properties are described by the tensor $\boldsymbol{\varepsilon}$, Maxwell's equations simplify to:

$$\begin{cases} \nabla \times \vec{E} = -\mu_0 \dfrac{\partial \vec{H}}{\partial t} \\ \nabla \times \vec{H} = \varepsilon_0 \boldsymbol{\varepsilon} \dfrac{\partial \vec{E}}{\partial t} \\ \nabla \cdot \left(\varepsilon_0 \boldsymbol{\varepsilon} \vec{E}\right) = 0 \\ \nabla \cdot \vec{H} = 0 \end{cases}$$

Eliminating the magnetic field $\vec{\boldsymbol{H}}$ yields the wave equation for the electric field $\vec{\boldsymbol{E}}$ :

$$\nabla^2 \vec{E} + \omega^2 \mu_0 \varepsilon_0 \boldsymbol{\varepsilon} \vec{E} = \nabla\left(\nabla \cdot \vec{E}\right)$$

For biaxial crystals, the dielectric tensor in the principal coordinate system is $\boldsymbol{\varepsilon} = \mathrm{diag}(\varepsilon_x, \varepsilon_y, \varepsilon_z)$. Substituting the electric field in the plane harmonic wave form, $\vec{\boldsymbol{E}}(\vec{\boldsymbol{r}}, \boldsymbol{t}) = \vec{\boldsymbol{E}}_{\boldsymbol{0}} \boldsymbol{e}^{\boldsymbol{i}(\vec{\boldsymbol{k}} \cdot \vec{\boldsymbol{r}} - \boldsymbol{\omega t})}$, into the wave equation and expanding the result gives a system of three-dimensional linear equations:

$$\begin{pmatrix} k_0^2 \varepsilon_x - k_y^2 - k_z^2 & k_x k_y & k_x k_z \\ k_x k_y & k_0^2 \varepsilon_y - k_x^2 - k_z^2 & k_y k_z \\ k_x k_z & k_y k_z & k_0^2 \varepsilon_z - k_x^2 - k_y^2 \end{pmatrix} \begin{pmatrix} E_x \\ E_y \\ E_z \end{pmatrix} = 0$$

where $k_0 = \omega / c$ denotes the wave number in vacuum. For non-trivial solutions to exist, the determinant of the coefficient matrix must vanish:

$$\begin{vmatrix} k_0^2 \varepsilon_x - k_y^2 - k_z^2 & k_x k_y & k_x k_z \\ k_x k_y & k_0^2 \varepsilon_y - k_x^2 - k_z^2 & k_y k_z \\ k_x k_z & k_y k_z & k_0^2 \varepsilon_z - k_x^2 - k_y^2 \end{vmatrix} = 0$$

Expanding this determinant yields the three-dimensional isofrequency surface (IFS) equation[3]:

$$\begin{gathered} \left(\varepsilon_x k_x^2 + \varepsilon_y k_y^2 + \varepsilon_z k_z^2\right)\left(k_x^2 + k_y^2 + k_z^2\right) + k_0^4 \varepsilon_x \varepsilon_y \varepsilon_z \\ -k_0^2\left[\varepsilon_x\left(\varepsilon_y + \varepsilon_z\right) k_x^2 + \varepsilon_y (\varepsilon_x + \varepsilon_z) k_y^2 + \varepsilon_z\left(\varepsilon_x + \varepsilon_y\right) k_z^2\right] = 0 \end{gathered}$$

This fourth-order equation in $k_x, k_y, k_z$ factors into two quadratic surfaces in momentum space, corresponding to electromagnetic waves with two distinct polarization modes: ordinary (o) and extraordinary (e) light.

For the fundamental case of hyperbolic modes, consider a uniaxial crystal. For the sake of simplicity, the dielectric functions considered here are all real. Here, the dielectric tensor satisfies $\varepsilon_x = \varepsilon_y \neq \varepsilon_z$ (the crystal's optic axis is parallel to the z-axis), and then the wavevector surface equation reduces to:

$$\left(k_x^2 + k_y^2 + k_z^2 - \varepsilon_x k_0^2\right)\left[\varepsilon_x\left(k_x^2 + k_y^2\right) + \varepsilon_z(k_z^2 - \varepsilon_x k_0^2\ )\ \right] = 0$$

Thus, we obtain an equation of a hyperboloid:

$$\varepsilon_z^{-1}\left(k_x^2 + k_y^2\right) + \varepsilon_x^{-1}k_z^2 = k_0^2$$

The hyperboloid is single-sheeted if $\varepsilon_z > 0,\ \varepsilon_x, \varepsilon_y < 0$ (type II) and two-sheeted if $\varepsilon_z < 0$, $\varepsilon_x, \varepsilon_y > 0$ (type I). In this case, it is referred to as an out-of-plane hyperbolic mode, since the x-y plane is isotropic, as the case of hexagonal boron nitride (hBN)[8].

For systems with lower symmetry, the dielectric function becomes more complex, resulting in different shapes of the hyperboloid. Passler et al. rigorously solve Maxwell's equations in monoclinic systems[9], so that we can draw the IFS of β-$Ga_2O_3$ in lossless case initially (Fig. S6), in which the imaginary part of each term in the dielectric tensor is neglected. After neglecting the influence of the isotropic mode, we can still divide the hyperbolic surface into type I and type II based on the number of sheets. When the dielectric function has off-diagonal terms in monoclinic systems, we can eliminate the optical axis dispersion by frequency-dispersive rotation, thereby making the off-diagonal terms contain only the imaginary part. To describe the rotation of the optical axes within the monoclinic plane, Passler et al. diagonalized the real part of the permittivity tensor at various frequencies by rotating the monoclinic plane using a frequency-dependent angle defined as[9]:

$$\gamma(\omega) = \frac{1}{2}\tan^{-1}\left(\frac{2\mathrm{Re}(\varepsilon_{xy}(\omega))}{\mathrm{Re}(\varepsilon_{xx}(\omega)) - \mathrm{Re}(\varepsilon_{yy}(\omega))}\right)$$

The rotation is performed via a rotation matrix $R(\theta)$, given by:

$$R(\theta) = \begin{bmatrix} \cos\theta & \sin\theta & 0 \\ -\sin\theta & \cos\theta & 0 \\ 0 & 0 & 1 \end{bmatrix}$$

The rotated coordinate system, denoted as $[mnz]$, is obtained through the transformation:

$$\boldsymbol{\varepsilon}_{[mnz]} = R(-\theta)\,\boldsymbol{\varepsilon}_{[xyz]}\,R(\theta)$$

with $\theta = \gamma(\omega)$. In this rotated frame, the real part of $\varepsilon_{mn}$ becomes zero. At this point, the off-diagonal terms only have a shear effect on the IFS and do not change its type. Furthermore, the imaginary part in the diagonal terms will cause the boundary of the IFS to become blurred, but it will still not change its type. Within the rotated framework, the type of polariton propagation in β-$Ga_2O_3$ can be unambiguously identified based on the real part of the diagonalized permittivity: in-plane hyperbolic type I (two-sheeted hyperboloid with one negative element) if $Re(\varepsilon_m) \times Re(\varepsilon_n) < 0$, $Re(\varepsilon_z) > 0$, in-plane hyperbolic type II if $Re(\varepsilon_m) \times Re(\varepsilon_n) < 0$, $Re(\varepsilon_z) < 0$ (one-sheeted hyperboloid with two negative elements), where $\varepsilon_m$ and $\varepsilon_n$ are in-plane diagonal terms[10].

**Note 8: IFC analysis of modulation of quasi-1D polaritons**

The calculations of the isofrequency contours (IFCs) were performed using a generalized 4 × 4 transfer matrix formalism[9]. In short, this formalism allows for the calculation of reflection and transmission coefficients across any number of stratified media with arbitrary dielectric tensors, thereby accounting for the anisotropy of the samples.

The theoretical calculation of IFC is in line with the experimental data in Fig. 2 of the main text and provides a more detailed description of the propagation characteristics of quasi-1D polariton. As shown in the first column of Fig. S9a (θ = -26°), the mode rotates anticlockwise from 600 $cm^{-1}$ to 659 $cm^{-1}$ along the increase of frequency while it rotates conversely from 659 $cm^{-1}$ to 690 $cm^{-1}$. The difference of rotation direction in these two frequency ranges is because the selected branches of modes of α-$MoO_3$ are different while the propagation angle of α-$MoO_3$ thin layer is sensitive to the frequency. In the frequency range of 600 $cm^{-1}$ to 690 $cm^{-1}$, the propagation angle of α-$MoO_3$ thin layer decreases as the frequency increases, making the propagation directions of the four branches shifts bidirectionally in other words. As a result, the rotation direction can also be reversed by changing the twist angle θ in the same frequency range, through which the different branches are selected. The IFCs of the cases of θ=-26° and θ=35° of the same frequencies are shown on the left and right part of Fig. S9a. The rotation direction along the increase of the frequency is marked in the figure.

## Note 9: Analysis of the pruning efficiency

To evaluate the pruning efficiency, we perform an integral of the angular distribution intensity I(θ) in *k*-space and compare the integrals of the quasi-1D polaritons (the brightest angular sector) and the eliminated branch (the second brightest angular sector) to get a figure of merit (FOM) for the measurement of pruning efficiency [11]. Specifically, we first define the angular distribution intensity I(θ). The total intensity within a small angular sector centered at θ with an angular width $\Delta\theta = 0.25°$ is expressed as

$$I(\theta)= \int_{\theta-\Delta\theta/2}^{\theta+\Delta\theta/2} \int_{1.5k_0}^{10k_0} I(r,\theta')r \, dr \, d\theta'.$$

Accordingly, the figure of merit (FOM) for pruning efficiency is defined as $1-I(\theta_2)/I(\theta_1)$ where $I(\theta_1)$ and $I(\theta_2)$ denote the largest and second-largest values of $I(\theta)$, respectively. Fig. S12 shows the influence of the substrate's intrinsic loss on the pruning efficiency. The loss factor is obtained by multiplying the imaginary part of the dielectric function in each direction of β-$Ga_2O_3$ by the corresponding coefficient simultaneously. The closer the FOM is to 100%, the more effective the suppression becomes. As shown in the Fig. S12c, reducing the intrinsic loss of β-$Ga_2O_3$ to 50% of its original value increases the FOM from a baseline of 70% to 80%, and further reducing it to 10% boosts the FOM to 90%. In brief, pruning efficiency can be enhanced by reducing intrinsic losses (i.e., low phonon absorption) to ensure efficient coupling.

## Note 10: The effects of loss on the propagation modes

To illustrate how loss affects the propagation mode in our modulation mechanism, mode analysis is performed here. Because of the phonon frequency of the β-$Ga_2O_3$ substrate, the study of the mode should be divided into two cases. β-$Ga_2O_3$ works as a normal dielectric material in the case of lower frequency band, while as a phonon polariton material in the case of higher frequency band. Mode analysis results of the case of lower frequency band are shown in Fig. S13a-d. The isofrequency contour of β-$Ga_2O_3$ bulk (green) and 200-nm thick α-$MoO_3$ flake (orange) is shown in Fig. S13a. As discussed in the manuscript, the branches of α-$MoO_3$, which are not covered by the isofrequency contour of β-$Ga_2O_3$, propagate on the surface of materials. Fig. S13b shows the z-component of electric field distribution of the fundamental TM mode of α-$MoO_3$, which is denoted with the blue box in Fig.

S13a. By placing the β-$Ga_2O_3$ substrate beneath the α-$MoO_3$, the mode profile of the heterostructure can be calculated, which is shown in Fig. S13c. The introduction of the β-$Ga_2O_3$ substrate increases the compression ratio of the mode from 29.5 to 39.5. The quality factor is decreased from 15.07 to 7.57. Reducing the loss of the substrate can enhance the quality factor to 10.60, while maintaining the compression factor. The mode profile is shown in Fig. S13d. In the mode analysis calculation in Fig. S13b-d, the imaginary part of the dielectric function of the α-$MoO_3$ is multiplied by 0.1 to see the propagation feature more clearly.

In the case of higher frequency band, mode analysis results are presented in Fig. S13 e-h (normal loss of α-$MoO_3$ is used). The excitation frequency is 680 $cm^{-1}$. The switch of the direction of the isofrequency contour of β-$Ga_2O_3$ leads to the selection of modes of α-$MoO_3$. The z-component of electric field distribution of the fundamental hyperbolic polariton mode of α-$MoO_3$ is shown in Fig. S13f, which is denoted with the blue box in Fig. S13e. The compression ratio of the mode is 5.7 and the quality factor is 15.90. The propagation mode of the heterostructure is shown in Fig. S13g. From the mode profile, the fundamental TM mode is converted into image polariton mode[12], which is caused by the high reflectivity of the substrate. In this frequency band, the sign of the Re($\varepsilon_{zz}$) becomes negative. The β-$Ga_2O_3$ substrate works as a phonon polariton material. The profile of the image mode is similar to the second-order hyperbolic polariton mode of α-$MoO_3$[12]. The compression ratio is 33.3 and the quality factor is 13.38. By decreasing the loss of the substrate, the mode has a longer propagation distance. The mode profile is shown in Fig. S13h. The compression ratio and quality factor are 33.4 and 20.44, respectively. It is worth noting that, both quality factor and compression ratio surpass the ones of the mode of freestanding α-$MoO_3$, which shows the potential of our mechanism to enhance the original mode by increasing the quality of the substrate.

**Note 11: Simulation of quasi-1D polariton**

The propagation of quasi-1D polariton is simulated with commercial finite element method software, COMSOL 6.2. The β-$Ga_2O_3$ is set as 10 μm to simulate infinite-thickness. The simulation area is 14 μm × 14 μm. A gold cylinder with diameter of 2.5 μm and height of 100 nm is placed in the center, on top of α-$MoO_3$. To simulate the excitation condition in practical experiment, a periodic port

with elevation angle of 52° and azimuth angle of 45° is set on the top boundary of the simulation area. The simulation results of twist angles of 26° is shown in Fig. S16. The excitation frequencies are set the same as the experiment results shown in Fig. 2. The propagation direction and rotation direction correspond well with experiment.

**Note 12: The effects of illumination orientation to quasi-1D polaritons**

The direction of incident light indeed affects the propagation characteristics and field strength of the quasi-1D polaritons, mainly by changing their excitation efficiency, but does not impact their quasi-1D features. Specifically, the incident light direction determines the excitation efficiency of polaritons through the adjustment of polarization. Previous studies[11] have shown that incident light on the gold disk can be regarded as a dipole polarized in the plane of incidence, and modes perpendicular to the polarization direction are most efficiently excited. Our experimental results are consistent with this observation.

In the α-$MoO_3$/β-$Ga_2O_3$ heterostructure, we systematically measured and simulated the behavior of quasi-1D polaritons at 680 $cm^{-1}$ under different incident light directions. As illustrated in Fig. S18, when the incident light direction was rotated by 45° increments, the propagation length and field distribution of the quasi-1D polaritons changed correspondingly: when the incident light was almost perpendicular to the polariton direction (Fig. S18a and S18e), the propagation length was longer and the field distribution clearer; in contrast, when the light was nearly parallel (Fig. S18c and S18h), the propagation length was reduced and the field intensity weakened. In summary, quasi-1D polaritons can be excited at all incident angles, although the excitation efficiency varies. Their propagation properties and anisotropy primarily originate from the heterostructure itself, and they show good robustness against changes in incident direction.

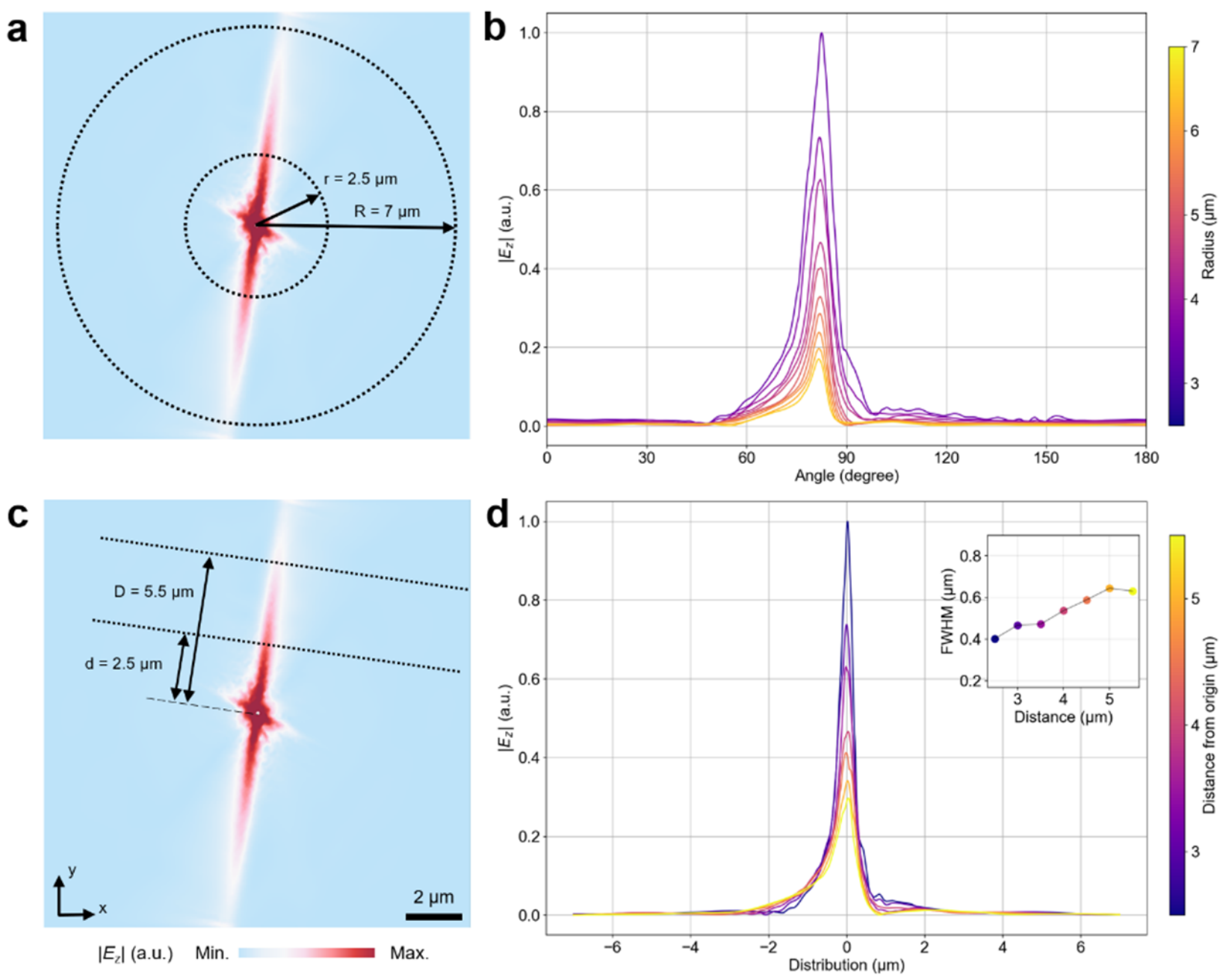


**Fig. S1: Analysis of real space distribution of quasi-1D polaritons.**

**a**, **c**, Two types of propagation analysis of electric field component |$\mathbf{E}_z$| of 630 $cm^{-1}$ generated by a point dipole of a 400 nm α-$MoO_3$/ bulk β-$Ga_2O_3$ heterostructure from simulations. The twist angle is 26°. **b**, Profiles of the simulations along circular cuts (black dotted circles in **a**) from 2.5-7 μm far from the dipole source. **d**, Profiles of the transverse field along the propagation direction. The data were extracted from a group of black dotted lines shown in **c**, which are located at a distance of 2.5-5.5 μm from the dipole source. The propagation direction is defined by the peak angular positions identified in panel **b**. The FWHM of the transverse field profile of different distance from the dipole source are displayed in the illustration in corresponding colors.

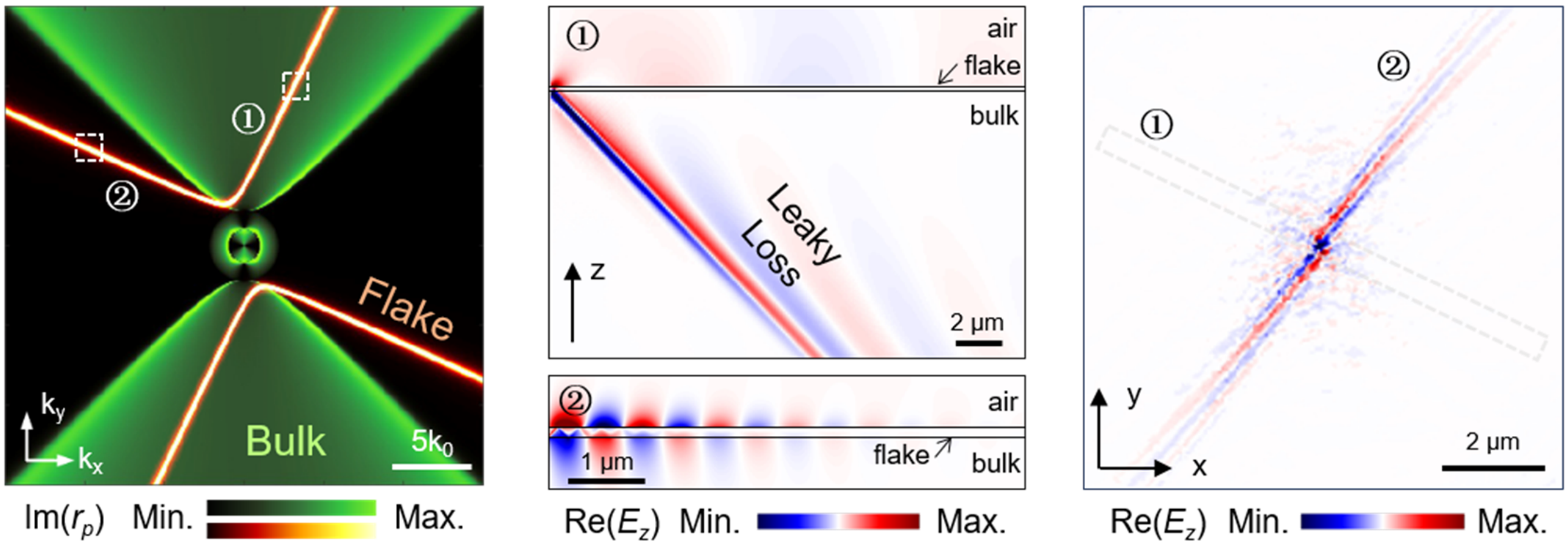


**Fig. S2: Leaking process revealed with mode analysis**

Formation mechanism of quasi-1D polaritons. Left panel: IFCs of the isolated 2D flake (orange) and 3D bulk (green). Middle panel: the mode analysis results of branches ① and ②. The specific analysis positions are marked with white dashed boxes in the left panel. Right panel: the resulting simulated Re($E_z$) field of the quasi-1D polariton, where branch ① disappears because of leaking into the bulk, compared with freestanding 2D flake's hyperbolic mode.

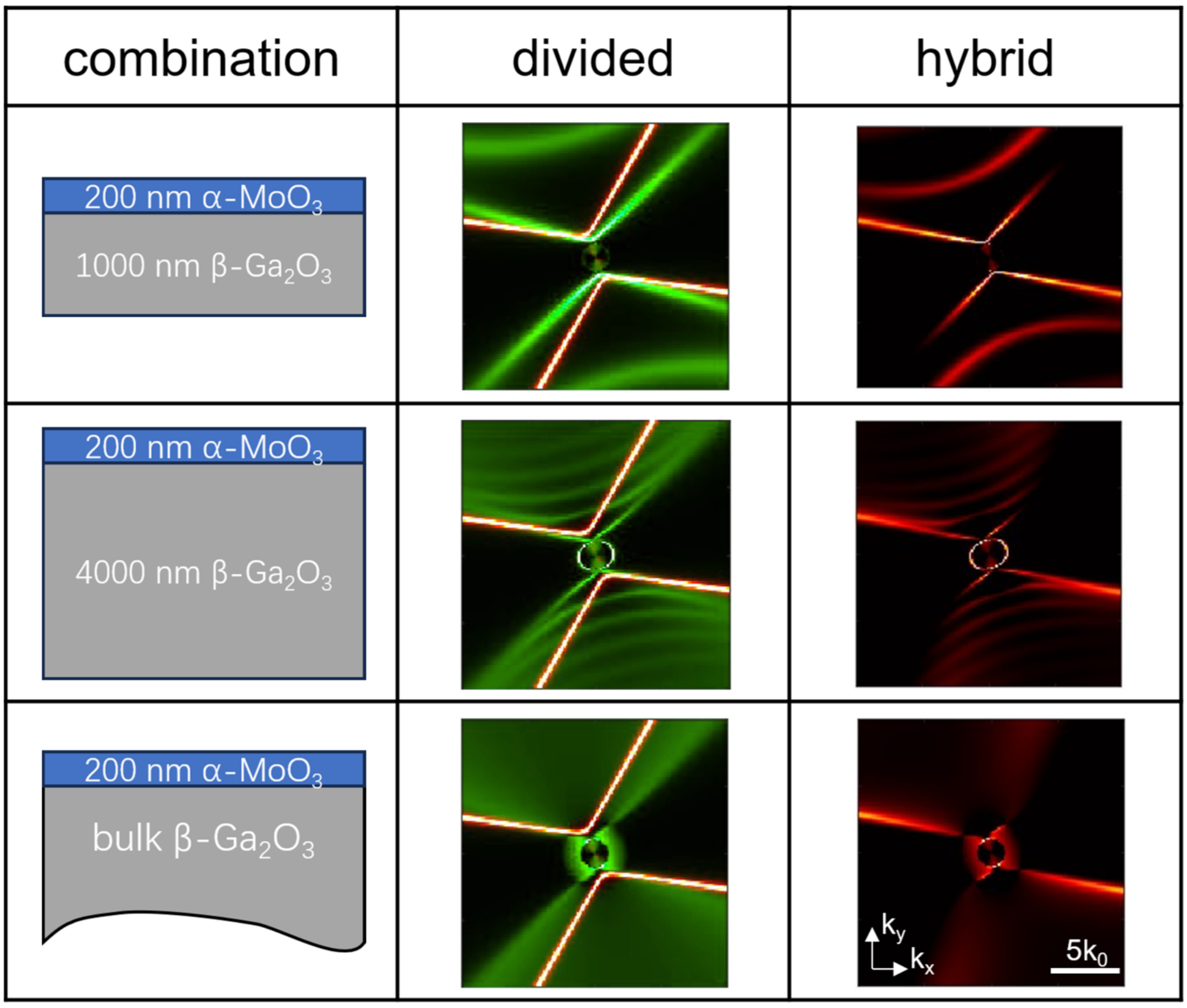


**Fig. S3: The influence of the substrate's thickness**

The dependence of α-$MoO_3$'s hyperbolic mode coupling and suppression on β-$Ga_2O_3$ substrate thickness underscores the necessity of a 3D substrate. The middle column presents the divided isofrequency contour of β-$Ga_2O_3$ bulk (green) and α-$MoO_3$ flake (orange) while the right column presents the hybrid isofrequency contour. With increasing thickness, β-$Ga_2O_3$'s hyperbolic higher-order modes densify into a continuum, leading to continuous coupling with α-$MoO_3$'s modes. Experimentally, a millimeter-thick substrate acts as an effective infinite-thickness medium, as the polariton is completely absorbed via leaky channels within the substrate, never reaching the bottom interface. The twist angle here is 26°.

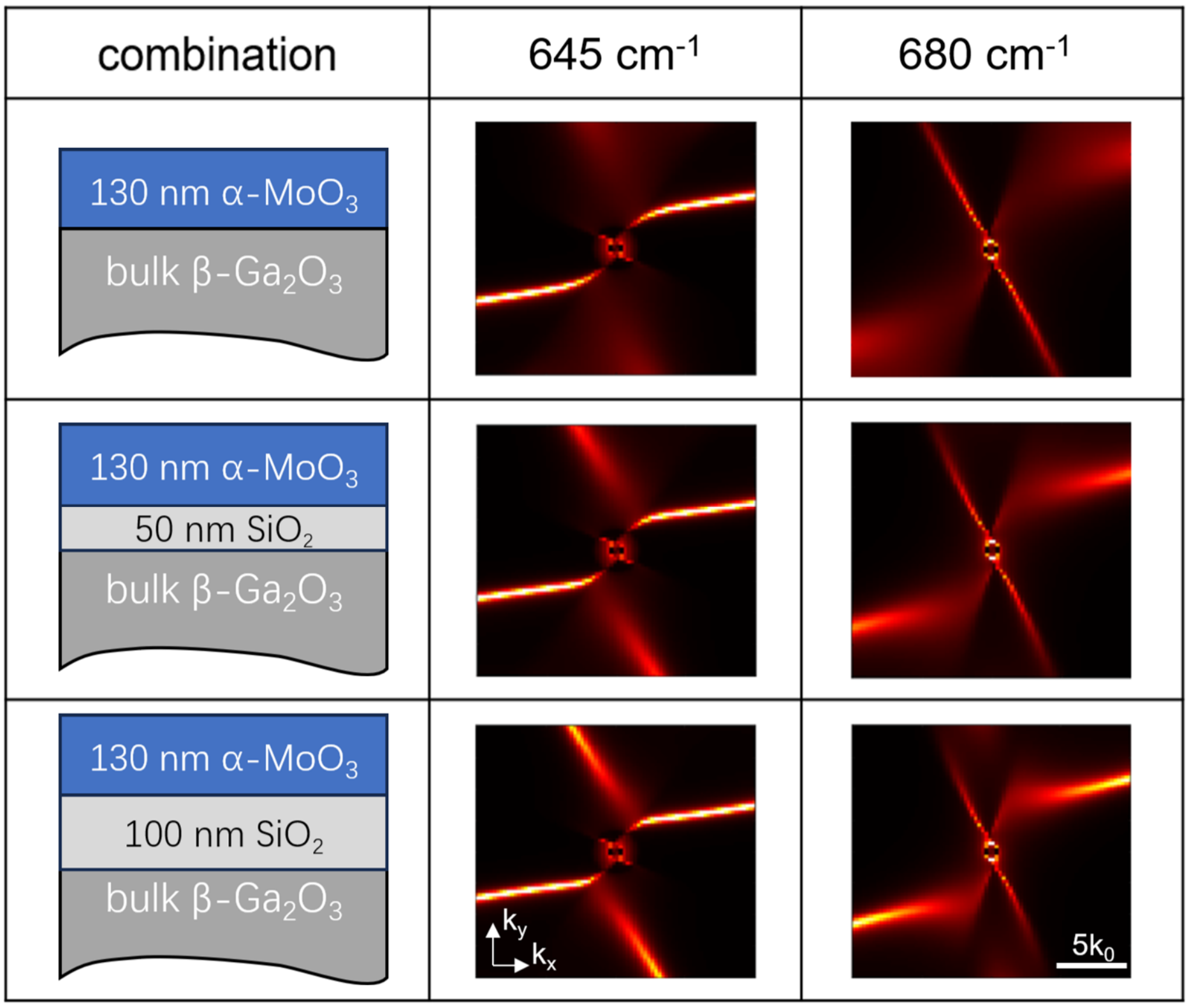


**Fig. S4: Tuning the coupling strength with dielectric medium interlayer**

The transition from 1D polaritons to hyperbolic modes is shown as the distance between the upper layer and substrate increases. In two different wavelength bands, as the thickness of the dielectric interlayer increases, the modes of the upper α-$MoO_3$ are less coupled to the substrate, thus maintaining the original hyperbolic mode more. The twist angle here is -26°.

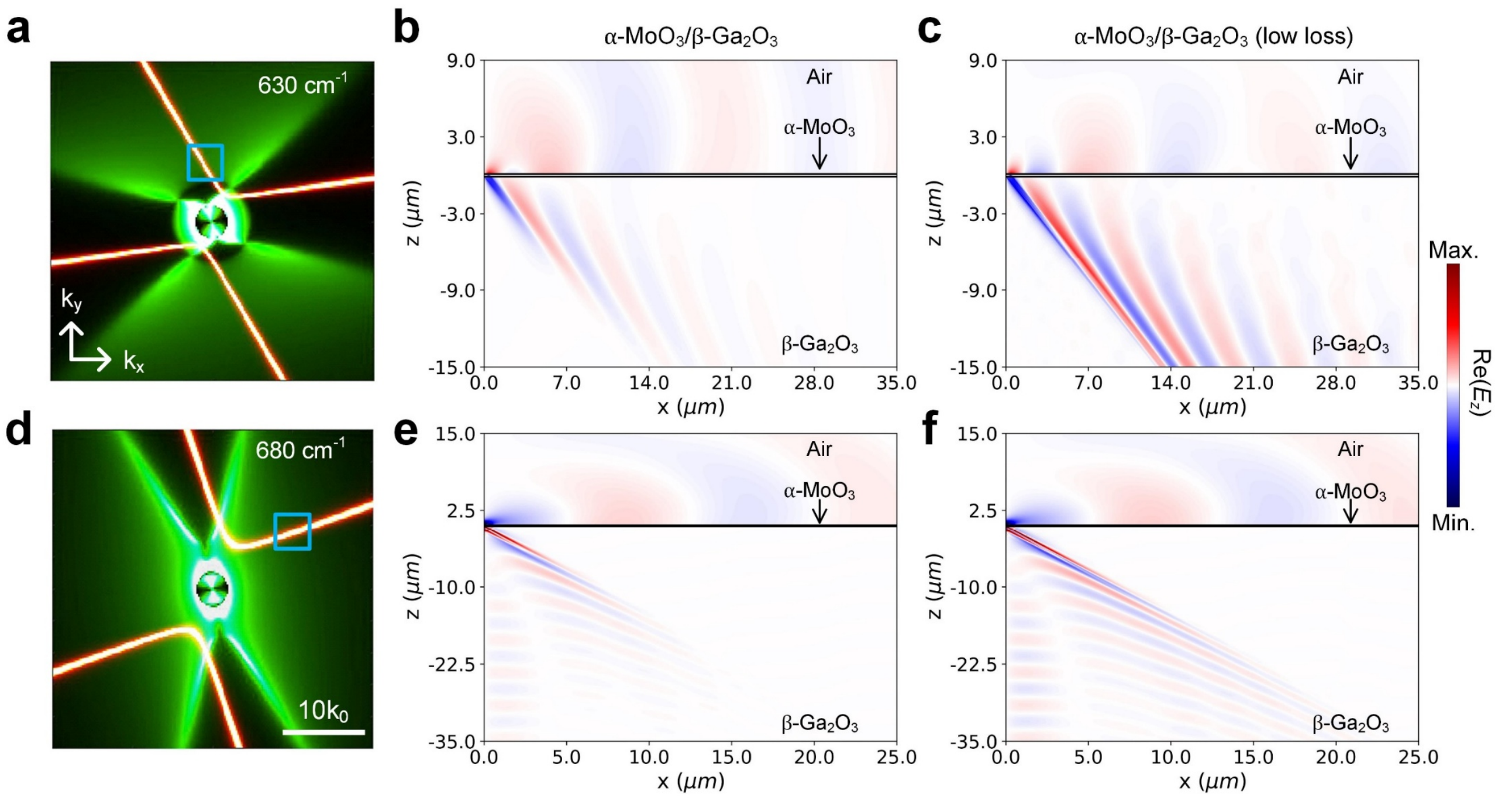


**Fig. S5: Mode analysis results of mode in the leaking directions of two frequency bands**

**a** and **d**, the isofrequency contour of β-$Ga_2O_3$ bulk (green) and 200-nm thick α-$MoO_3$ flake (orange) for 630 $cm^{-1}$ and 680 $cm^{-1}$, respectively. **b**, the z-component of the electric field of the heterostructure, denoted with the blue box in a. The β-$Ga_2O_3$ substrate has normal loss ($\varepsilon_{xx}$=1.6542+0.19985i, $\varepsilon_{yy}$=-5.4726+0.95184i, $\varepsilon_{zz}$=6.15+0.33194i, $\varepsilon_{xy}$=1.9445-0.19517i). **c**, the mode profile of the mode with 0.1 time of loss in β-$Ga_2O_3$ ($\varepsilon_{xx}$=1.6542+0.019985i, $\varepsilon_{yy}$=-5.4726+0.095184i, $\varepsilon_{zz}$=6.15+0.033194i, $\varepsilon_{xy}$=1.9445-0.019517i). **e** and **f**, the mode profile of the heterostructure of 680 $cm^{-1}$, with normal ($\varepsilon_{xx}$=8.5965+0.33336i, $\varepsilon_{yy}$=-1.1628+0.30793i, $\varepsilon_{zz}$=-9.6817+0.74983i, $\varepsilon_{xy}$=1.6747-0.027947i) and low loss ($\varepsilon_{xx}$= 8.5965+ 0.16668i, $\varepsilon_{yy}$=-1.1628+ 0.153965i, $\varepsilon_{zz}$=-9.6817+ 0.374915i, $\varepsilon_{xy}$=1.6747-0.0139735i), respectively.

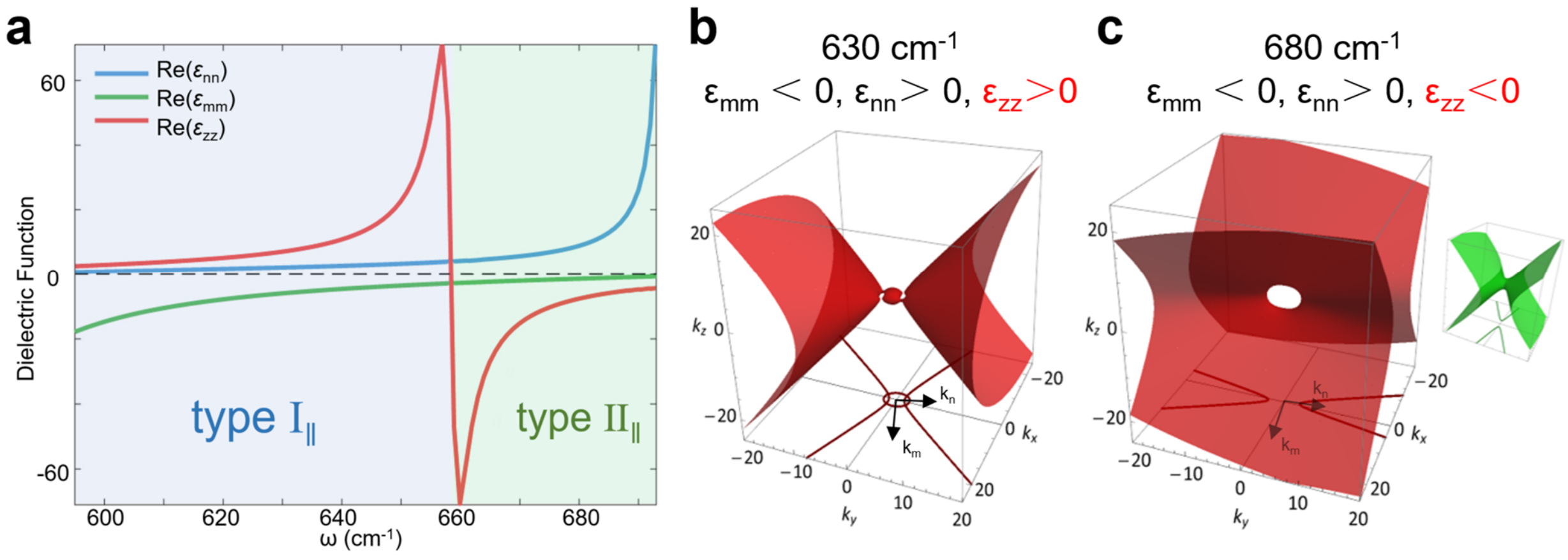


**Fig. S6: Types of in-plane hyperbolic modes of β-$Ga_2O_3$.**

**a**, Dielectric function of β-$Ga_2O_3$ in the frequency-dispersive rotated coordinate system. There's a sign reverse of Re($\varepsilon_{zz}$) at the out-of-plane phonon frequency 659 $cm^{-1}$, resulting in a change of types of its in-plane hyperbolic modes. **b**, **c**, IFS of type I and type II in-plane hyperbolic modes of β-$Ga_2O_3$ in lossless case. The contour lines at $k_z = 0$ are plotted as solid lines at the bottom, meanwhile the dispersive coordinate axes after rotation around the $k_z$ axis are labeled with $k_m$ and $k_n$, which correspond to the symmetry axes of the hyperbola. The inset in c provides another perspective to facilitate the observation of the single-sheet hyperbolic surface.

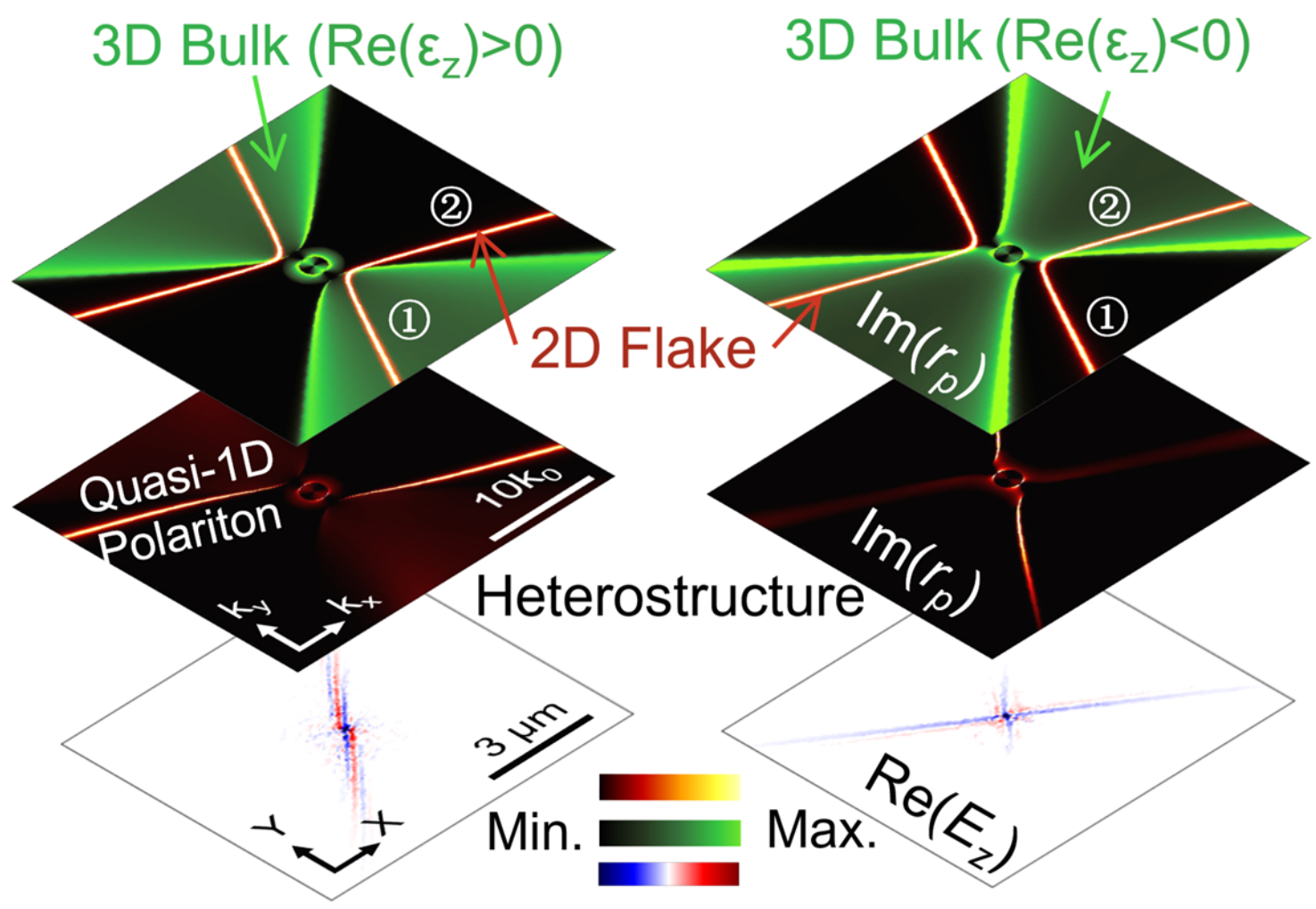


**Fig. S7: The switching mechanism of out-of-plane dielectric function**

Panels show: (top) IFCs of the isolated 2D flake (orange) and 3D bulk (green), (middle) IFCs of their heterostructure, and (bottom) the resulting simulated Re($E_z$) field of the quasi-1D polariton. The difference between the left and right column is the sign of Re($\varepsilon_z$), while keeping Re($\varepsilon_x$)·Re($\varepsilon_y$)<0. Branches ① and ② are marked out to compare the cases of leaky loss and retainment. The dielectric functions of overlayer: $\varepsilon_{x1}$ = 5+0.1i, $\varepsilon_{y1}$ = -5+0.1i, $\varepsilon_{z1}$ = 5+0.1i; substrate: $\varepsilon_{x2}$ = 5+0.1i, $\varepsilon_{y2}$ = -5+0.1i, $\varepsilon_{z2}$ = -5+0.1i. The twist angle between the overlayer and the substrate is 20°. The thickness of the 2D flake is 400 nm.

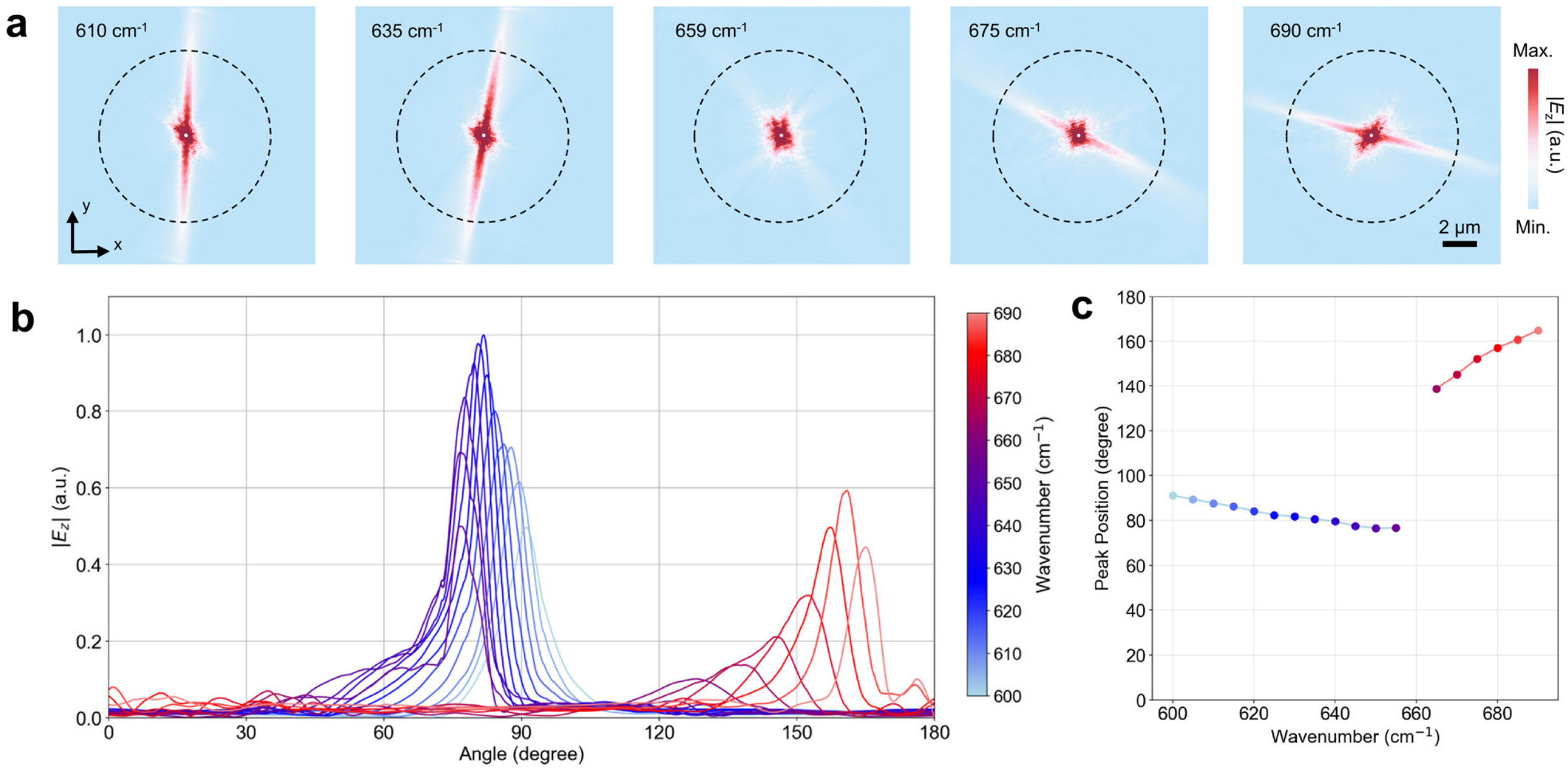


**Fig. S8: Quantitative analysis of frequency-dependent rotation of quasi-1D polaritons.**

**a**, Electric field component $|\mathbf{E}_z|$ at different frequencies generated by a point dipole of a 400 nm-thick α-$MoO_3$ film / bulk β-$Ga_2O_3$ heterostructure from simulations. The twist angle is 26°. **b**, Simulation profiles along circular cuts at 5 μm far from the dipole source (dashed line in panel **a**), with colors denoting cases of distinct wavenumbers. **c**, The extracted peak positions in **b** of different wavenumbers (except for the case of 659 $cm^{-1}$) shows opposite rotation directions on both sides of 659 $cm^{-1}$.

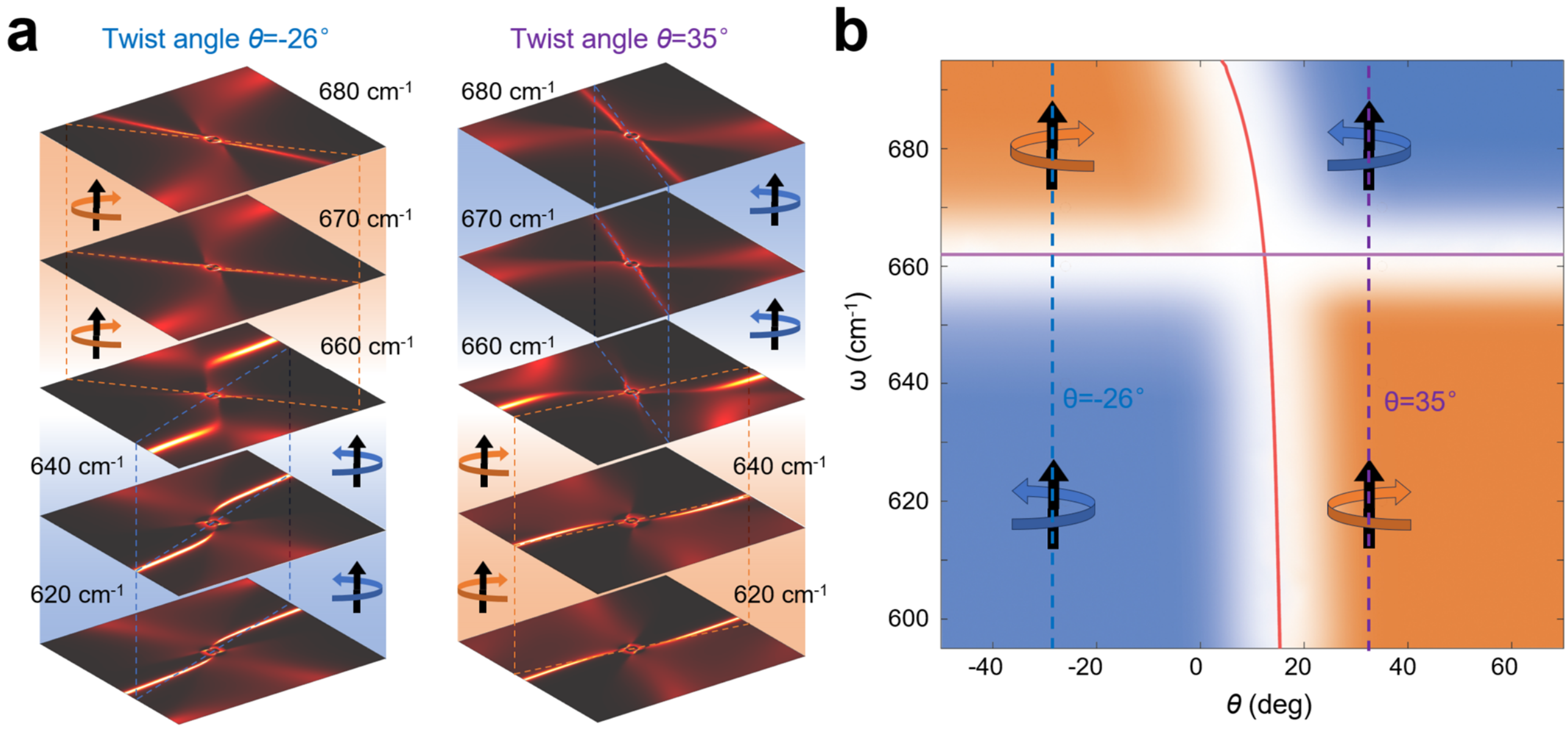


**Fig. S9: Frequency- and twist-dependent rotation reversal of quasi-1D polaritons**

**a**, The rotation of quasi-1D polaritons along the increase of frequency. It shows reciprocal switching of rotation directions at different twist angles. **b**, Rotation direction of the quasi-1D polaritons along the frequency increase varying with twist angle. At a given twist angle, orange color represents rotating clockwise and blue color represents rotating anticlockwise along the frequency increase. The red solid line represents the rotation angle of hyperbola axis of β-$Ga_2O_3$ with changing frequency. The purple solid line represents the frequency of an out-of-plane TO phonon modes. The two twist angles in **a**. are showed with blue and purple dash lines.

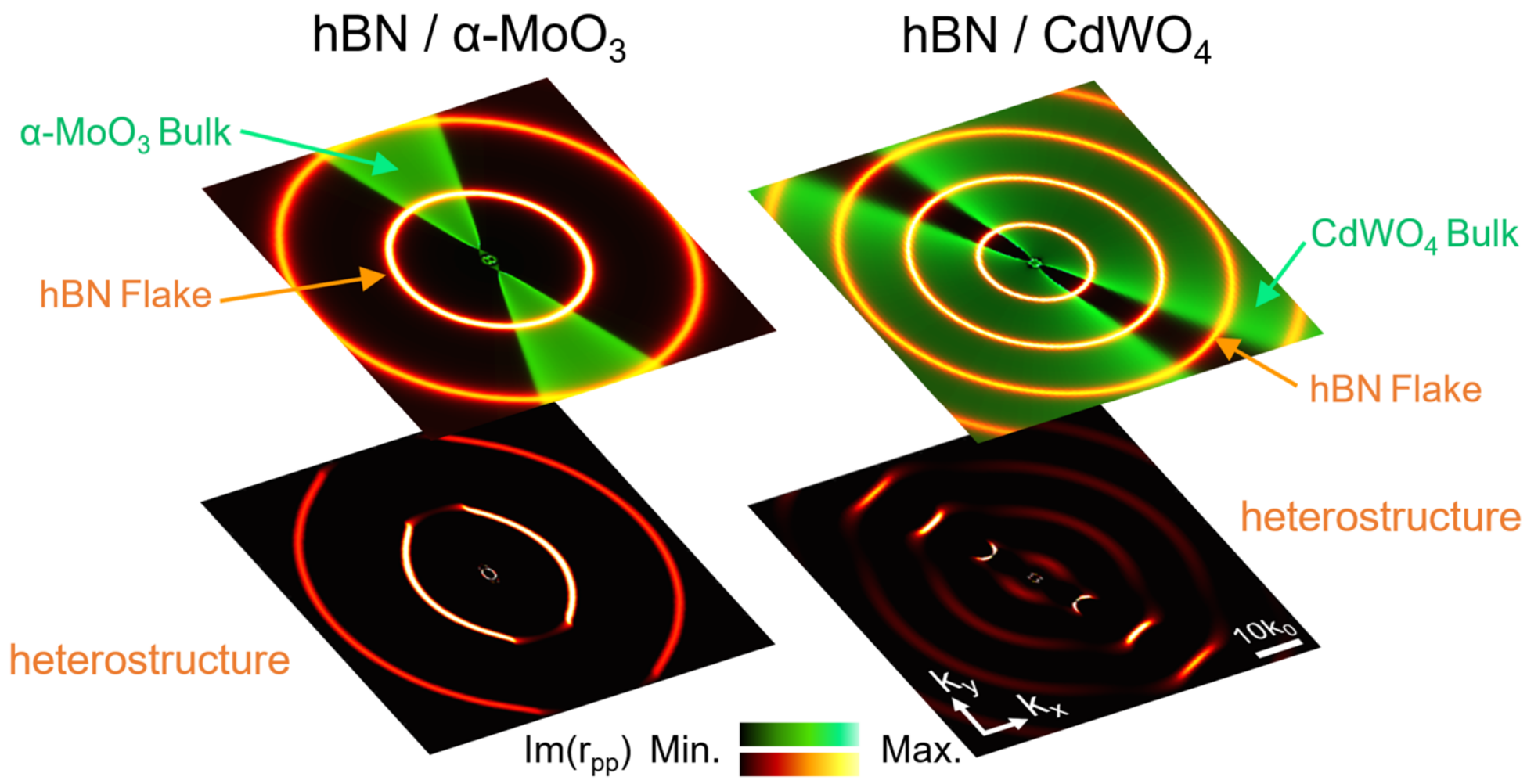


**Fig. S10: Tailoring of in-plane isotropic modes via the leaky channel mechanism**

Top panel: IFCs of the isolated flake (orange) and bulk (green). Bottom panel: IFCs of their heterostructure, where part of the circles disappears because of leaking into the bulk, compared with freestanding isolated hBN flake. Left: 200-nm-thick hBN flake stacked on bulk α-$MoO_3$ at 770 $cm^{-1}$. Right: 400-nm-thick hBN flake stacked on bulk $CdWO_4$ at 795 $cm^{-1}$. The universality of this mechanism is demonstrated by varying flake thickness, excitation frequency, and substrate material.

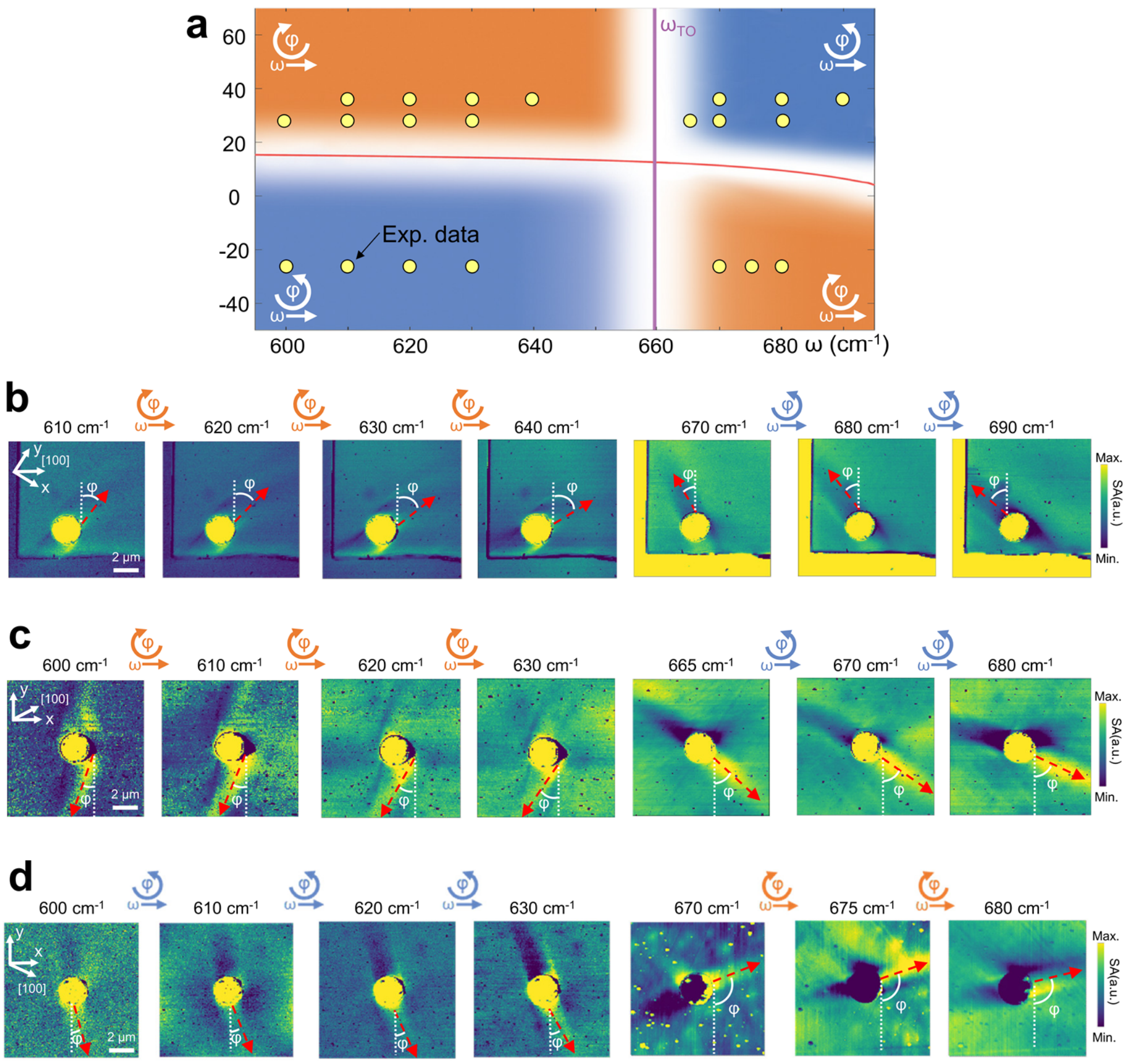


**Fig. S11: Experimental results of α-$MoO_3$/β-$Ga_2O_3$ heterostructure with other twist angles**

**a**, Mapping diagram of frequency-dependent rotation varying with twist angle and frequency. The vertical axis of this plot gives the range of twist angle that supports quasi-1D polariton in α-$MoO_3$/β-$Ga_2O_3$ heterostructure. The experimental result points are marked in the diagram. **b,** Measured results for the case of 33°, corresponding to the first row of yellow dots in panel **a**. The gold disk is near the boundary. For excitation frequency higher than the phonon frequency (659 $cm^{-1}$), the β-$Ga_2O_3$ substrate has high reflection because of the negative sign of $\varepsilon_{zz}$. **c, d,** Cases of 26° and -26°, corresponding to the second and third row of yellow dots in panel **a**, respectively. The thickness of α-$MoO_3$ flake is 85 nm in **b**, 100 nm in **c** and 80 nm in **d**.

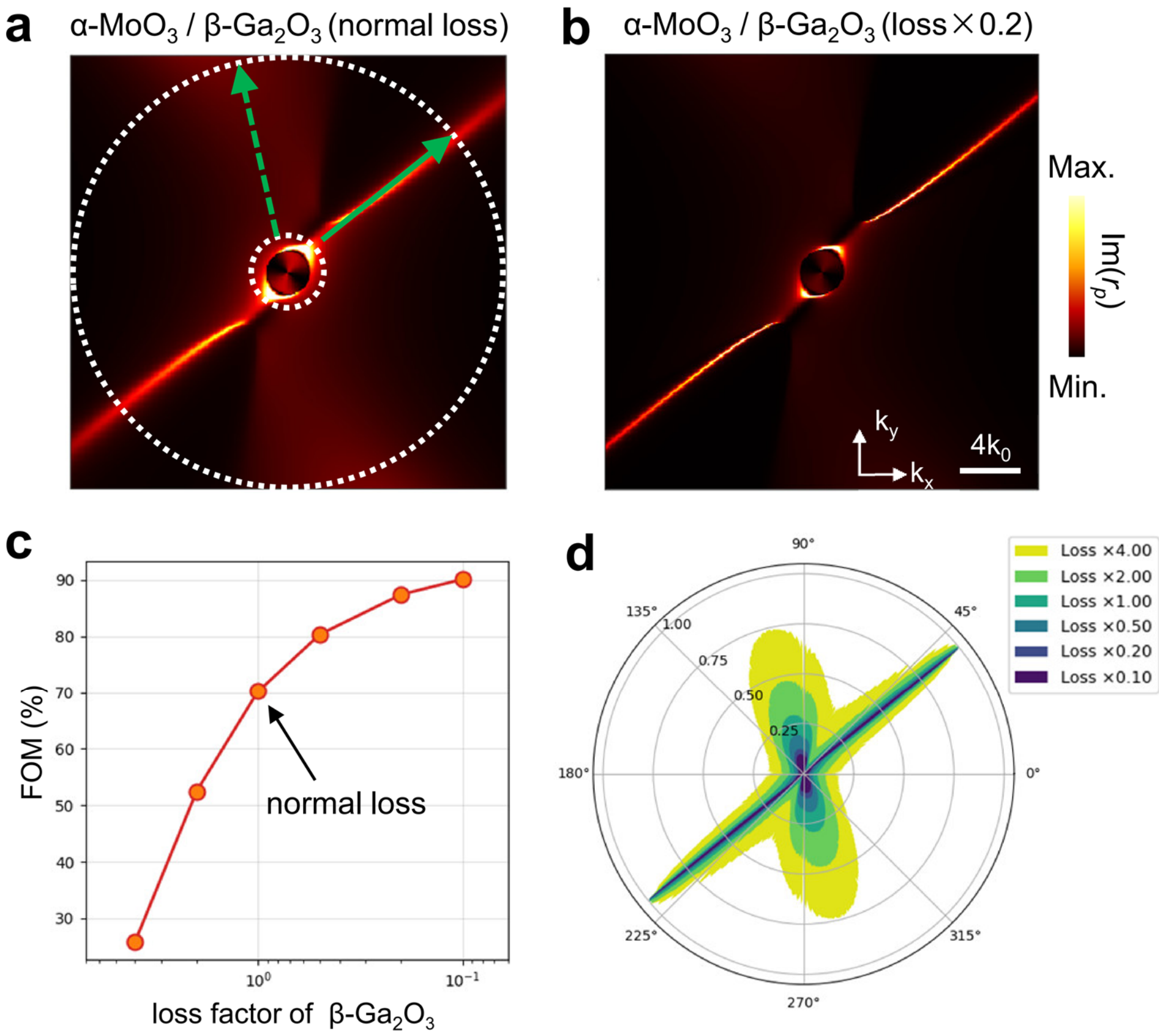


**Fig. S12: Calculation of pruning efficiency.**

**a, b,** IFCs of heterostructure of 200 nm-thick α-$MoO_3$ / bulk β-$Ga_2O_3$ with normal loss (**a**) and with 0.2 times of loss (**b**). **c**, FOM of pruning efficiency as a function of the loss factor in logarithmic scale. The direction of the largest (the second largest) value of I(θ) is indicated by a green solid (dashed) arrow in **a**. The loss factor of 1 indicates the normal condition. **d**, Polar overlay of the normalized angular integral profiles for all six loss multipliers. Each curve is individually normalized to its maximum.

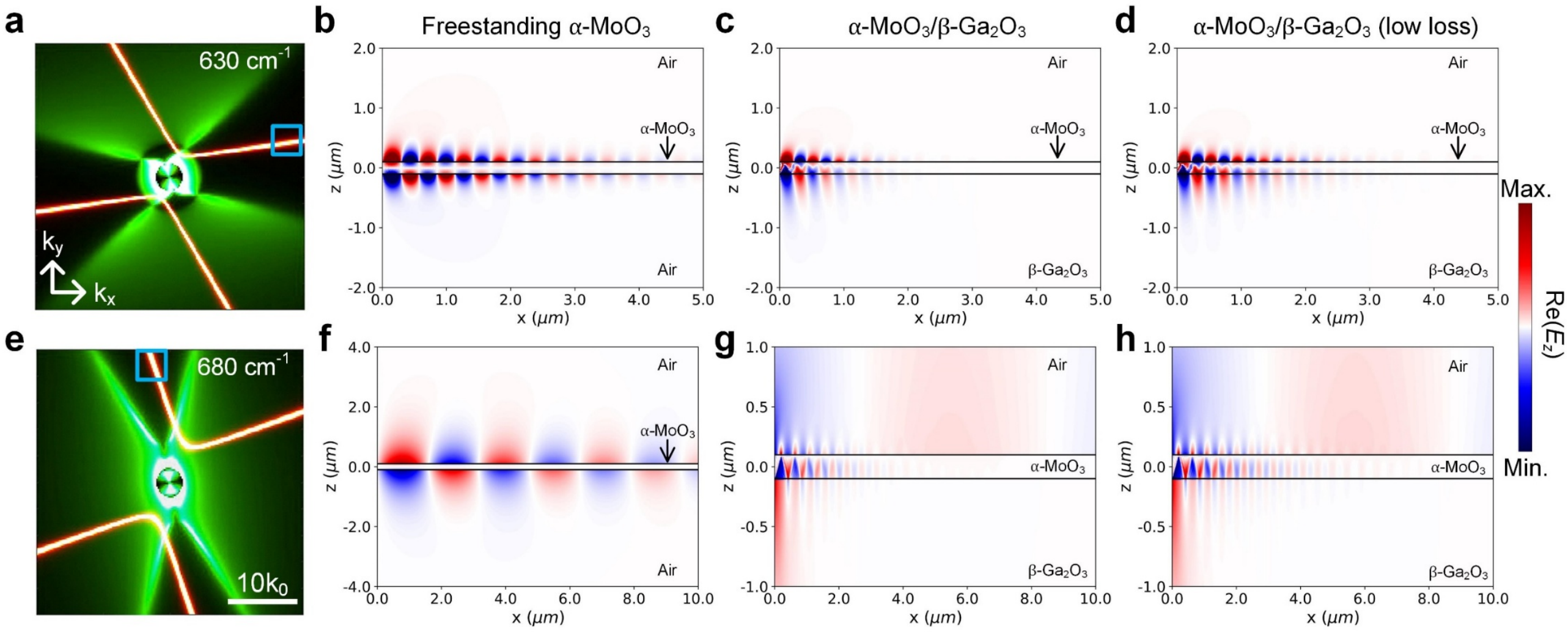


**Fig. S13: Mode analysis results of propagation modes in two frequency bands**

**a** and **e**, The isofrequency contour of β-$Ga_2O_3$ bulk (green) and 200-nm thick α-$MoO_3$ flake (orange) of different frequencies. **b** and **f**, the z-component of electric field distribution of the fundamental TM mode of α-$MoO_3$, which is denoted with the blue boxes in **a** and **e**. **c**, the field distribution of the propagation mode of the heterostructure. **d**, by decreasing the loss of the substrate, the mode has a longer propagation distance. **g**, the propagation mode of the heterostructure. The fundamental TM mode is compressed into image polariton mode, which is caused by the high reflectivity of the substrate. **h**, by decreasing the loss of the substrate, the mode has a longer propagation distance. To simulate the low-loss β-$Ga_2O_3$ substrate in our calculations, the imaginary part of its dielectric function was reduced to 10%.

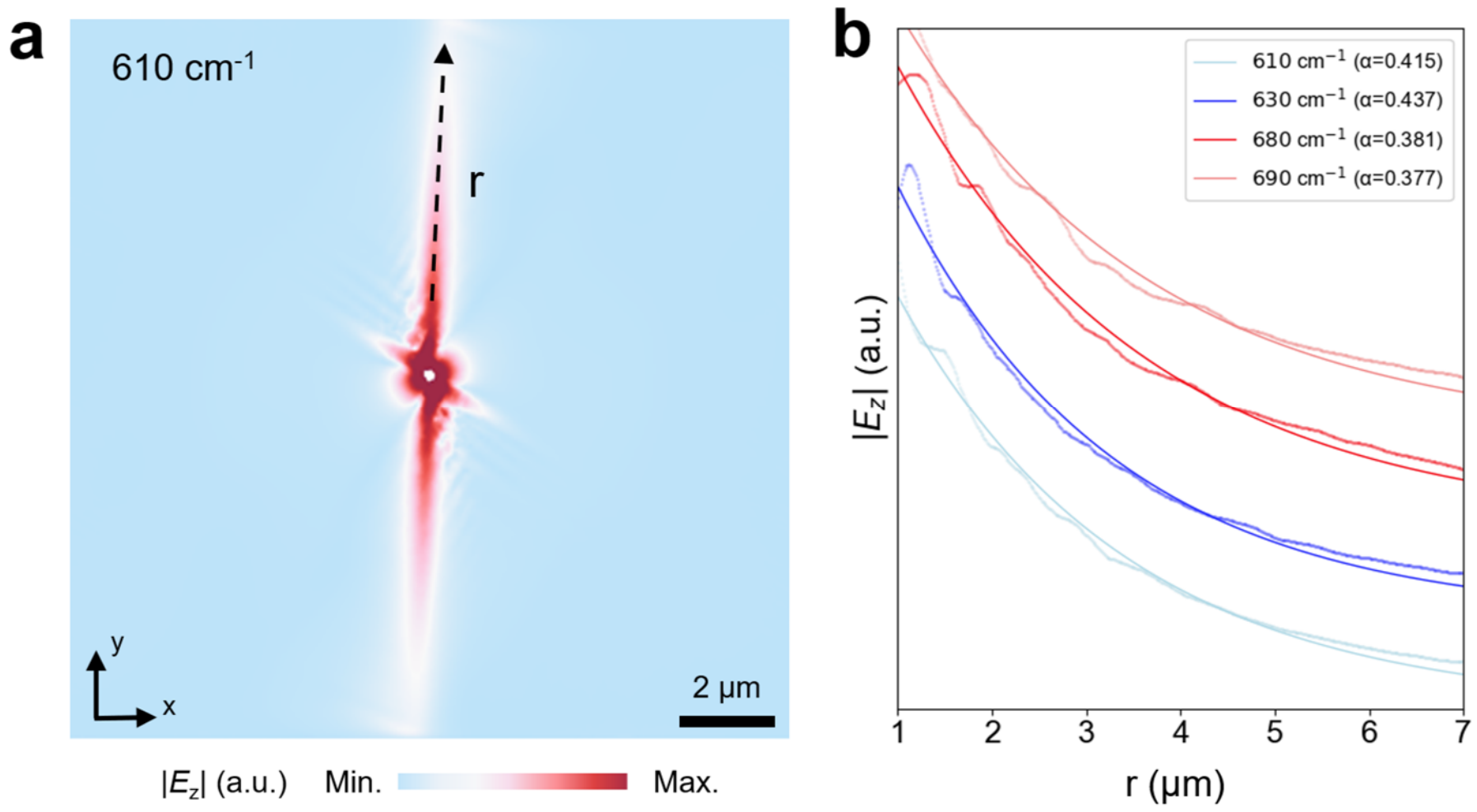


**Fig. S14: Propagation analysis of quasi-1D polaritons generated by a point dipole.**

**a**, Electric field component $|\mathbf{E}_z|$ of 610 $cm^{-1}$ generated by a point dipole of a 400 nm-thick α-$MoO_3$ film / bulk β-$Ga_2O_3$ heterostructure from simulations. The twist angle is 26°. **b**, Simulation profiles (scattered points) along the wavenumber-dependent propagation directions shown for distances of 1–7 μm from the dipole source (dashed line in panel **a** illustrates one example), with colors denoting cases of distinct wavenumbers. Different colored solid lines represent the curves fitted according to an exponential function $e^{-\alpha r}$. The attenuation coefficients α of different frequencies are displayed in the legend, and r represents the distance from the excitation source [1]. The calculated propagation length (i.e., 1/α) corresponding to the electric field amplitude decay to 1/*e* ranges from 2.3 to 2.7 μm.

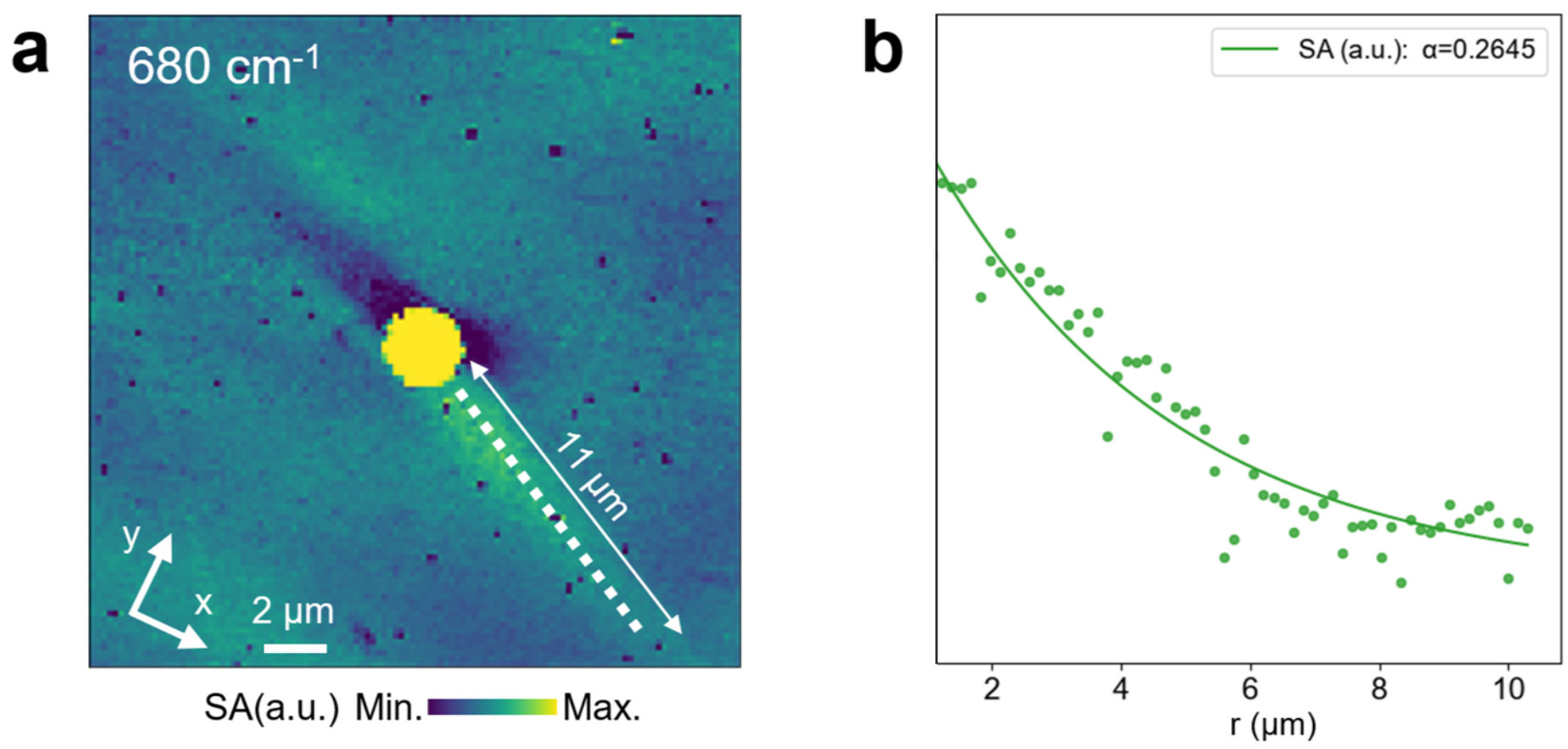


**Fig. S15: Propagation analysis of quasi-1D polaritons generated by a gold disk.**

**a,** near-field images of 680 $cm^{-1}$ generated by a gold disk of a 100 nm-thick α-$MoO_3$ film / bulk β-$Ga_2O_3$ heterostructure. The twist angle is 26°. **b**, experimental profiles (scattered points) along dashed line in panel **a**. Solid line represents the curve fitted according to $e^{-\alpha r}$. The attenuation coefficients α are displayed in the legend, and r represents the distance from the excitation source. We calculated propagation length (i.e., 1/α) which is ~3.8 μm. Additionally, near-field experimental image reveals that the quasi-1D polaritons form ray-like (non-diffracting) patterns extending over a distance up to 11 μm, as shown in **a** (following the definition of polariton characteristic length in reference [13]).

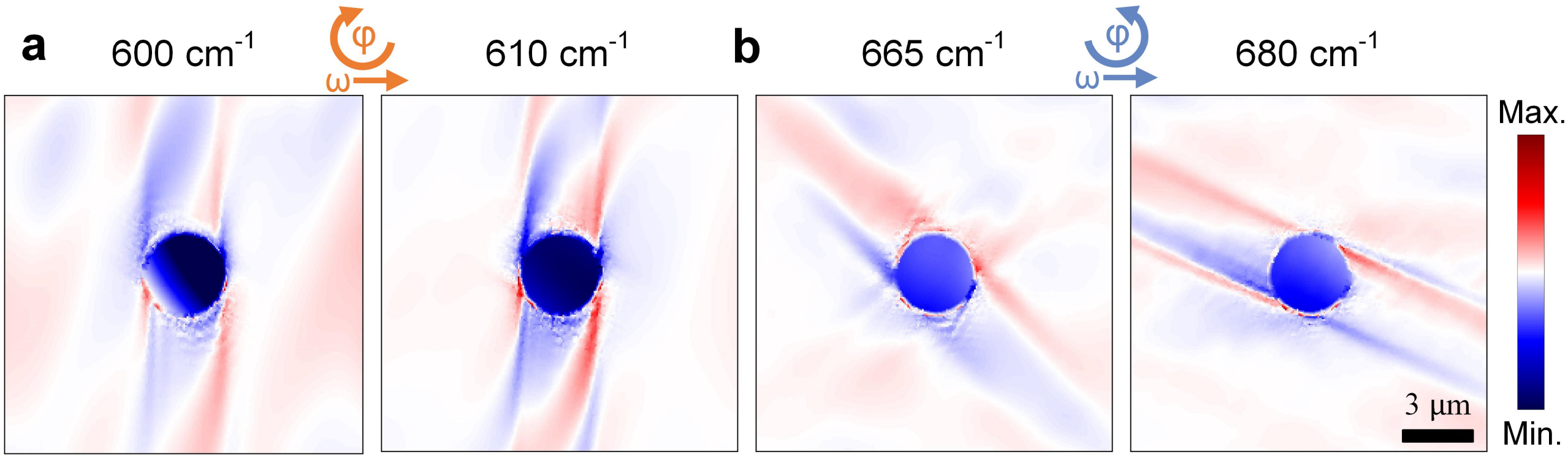


**Fig. S16: The simulation results for the case of 26° twist angle**

The simulation results of real part of the z-component of electric field of heterostructure consisting of 100 nm α-$MoO_3$ and 10 μm β-$Ga_2O_3$. The rotation direction of the mode corresponds well with the experiment.

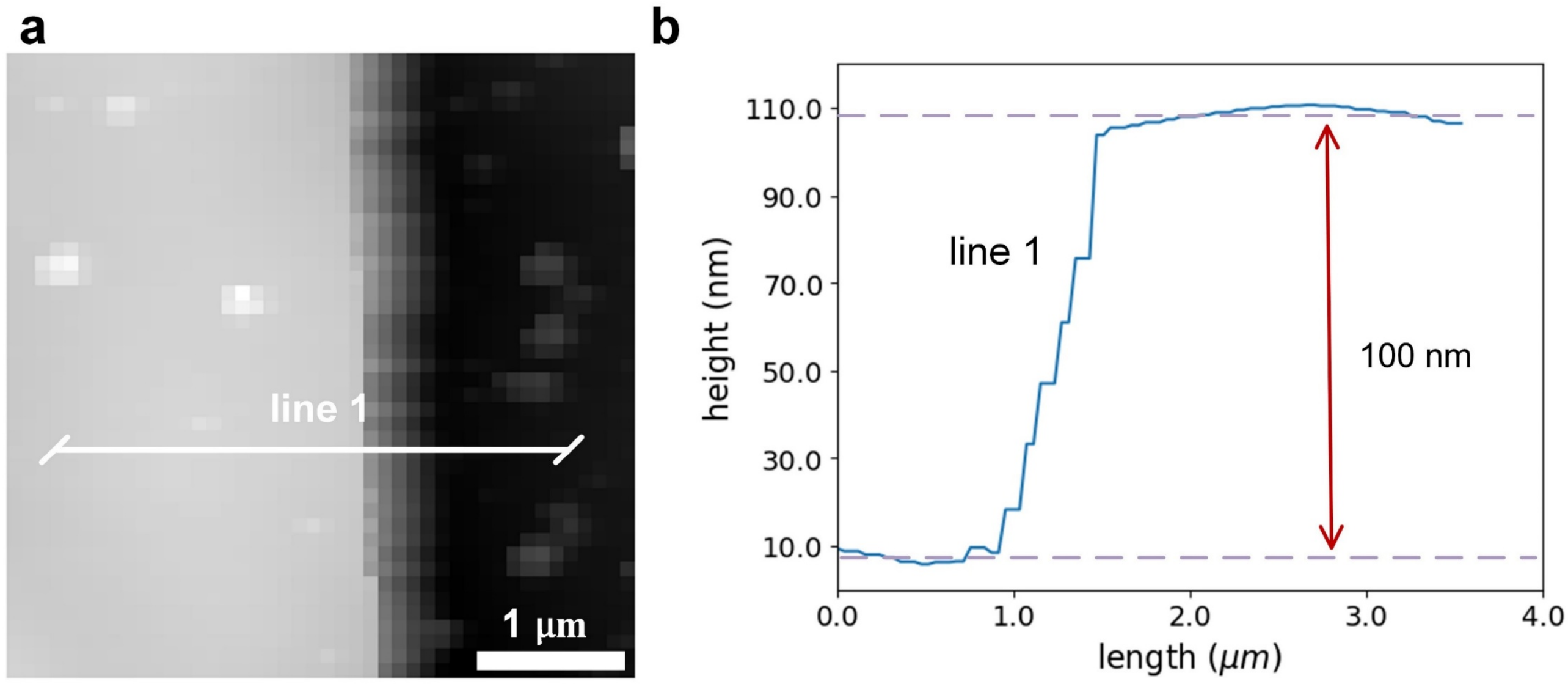


**Fig. S17: AFM figure of the measured sample**

**a,** The AFM figure of the measured sample with twist angle of 26°. **b,** the height measurement along the line in panel **a**. The height of the sample is about 100 nm.

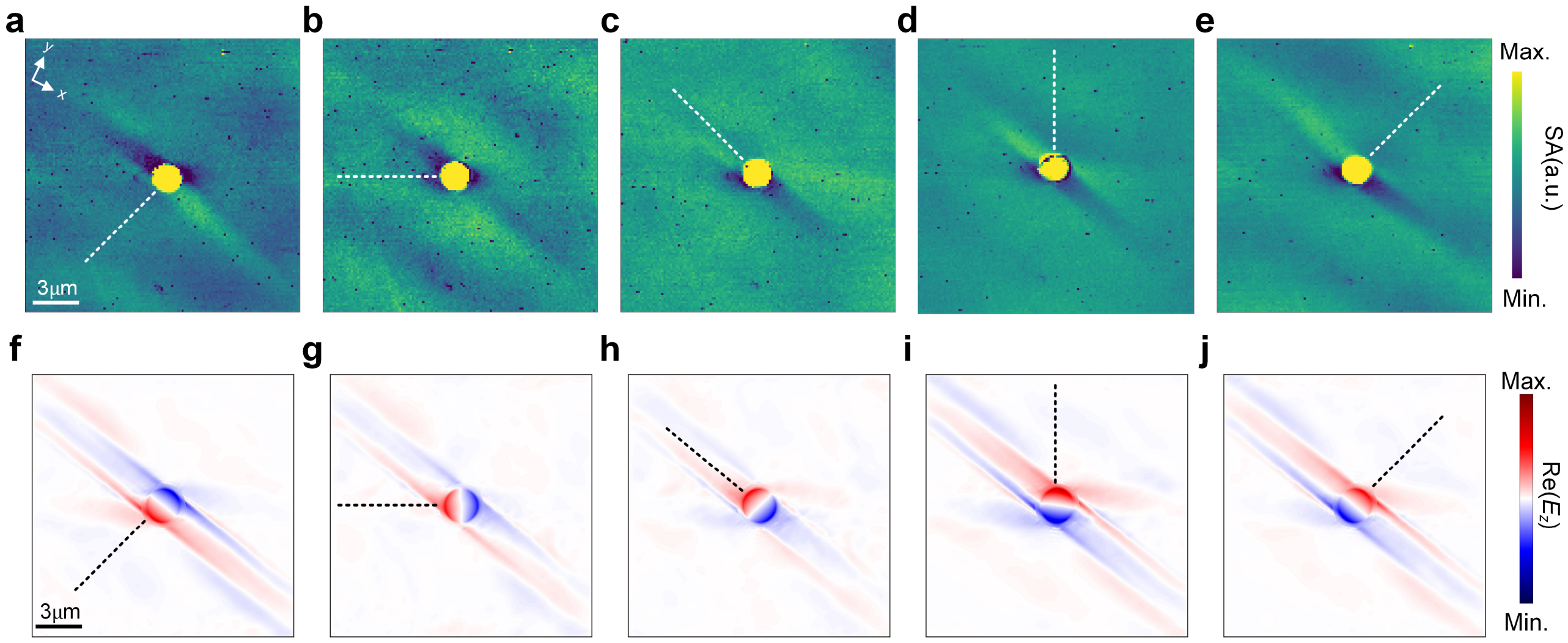


**Fig. S18: The dependence on illumination orientation of quasi-1D polaritons.**

**a-e,** Experimental near-field images of quasi-1D polaritons, excited by a 2.5 μm gold disk at the frequency of 680 $cm^{-1}$. The white dashed line illustrates the illumination direction of the input light, which rotates in 45-degree steps. The thickness of α-$MoO_3$ is 100 nm and the twist angle is 26°. **f-j**, Simulation of quasi-1D polaritons propagation with conditions according to (**a**–**e**).

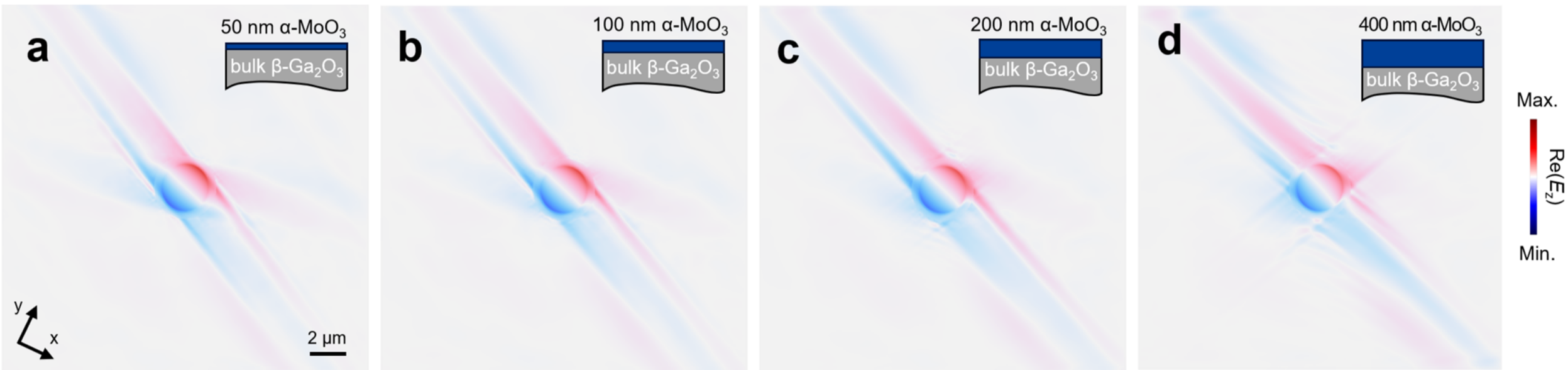


**Fig. S19: Simulation of propagation with different flake thickness.**

The real part of z-component of electric field distribution of the heterostructure with the α-$MoO_3$ flake thickness of 50 (**a**), 100 (**b**), 200 (**c**), and 400 (**d**) nm. The frequency is 680 $cm^{-1}$. Overall, the propagation length of quasi-1D polaritons exhibit robustness with respect to the thickness of the flake varied from 50 to 400 nm.

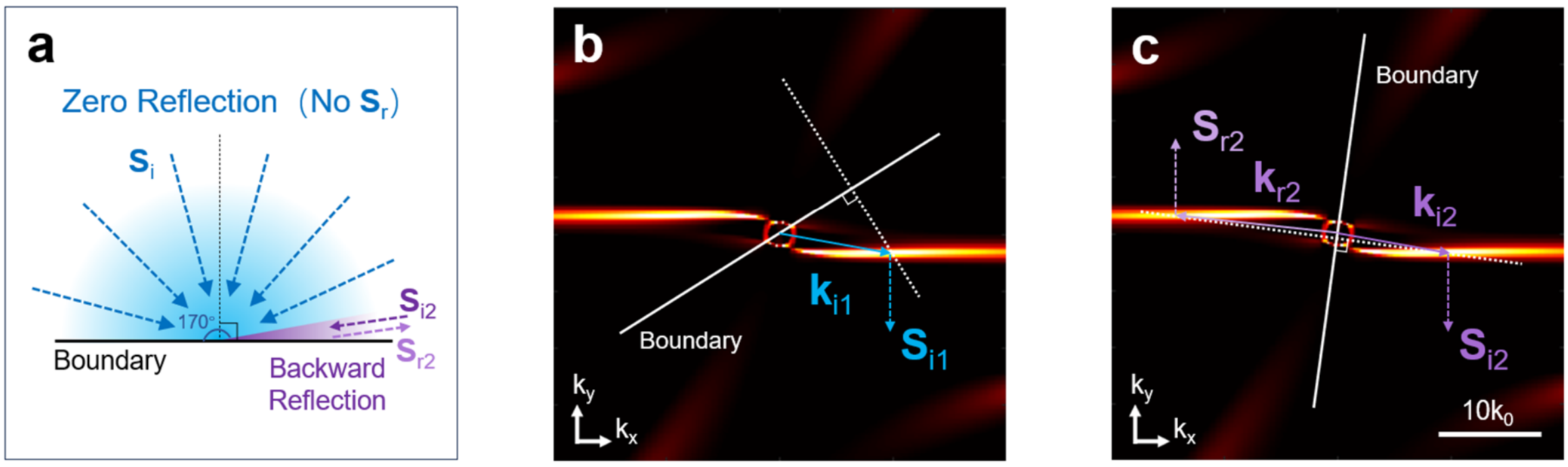


**Fig. S20: Interface angle dependence of zero reflection of quasi-1D polaritons**

**a**, Schematic of interface angle dependence of zero reflection in real space. **b**, Theoretical analysis of zero reflection of polaritons in k-space at 600 cm$^{-1}$. **c**, Theoretical analysis of backward reflection at 600 cm$^{-1}$. Based on the phase-matching requirement, the incident wavevector $k_{i2}$ and reflect wavevector $k_{r2}$ are marked. Their associated Poynting vector $S_{i2}$ and $S_{r2}$ are antiparallel.

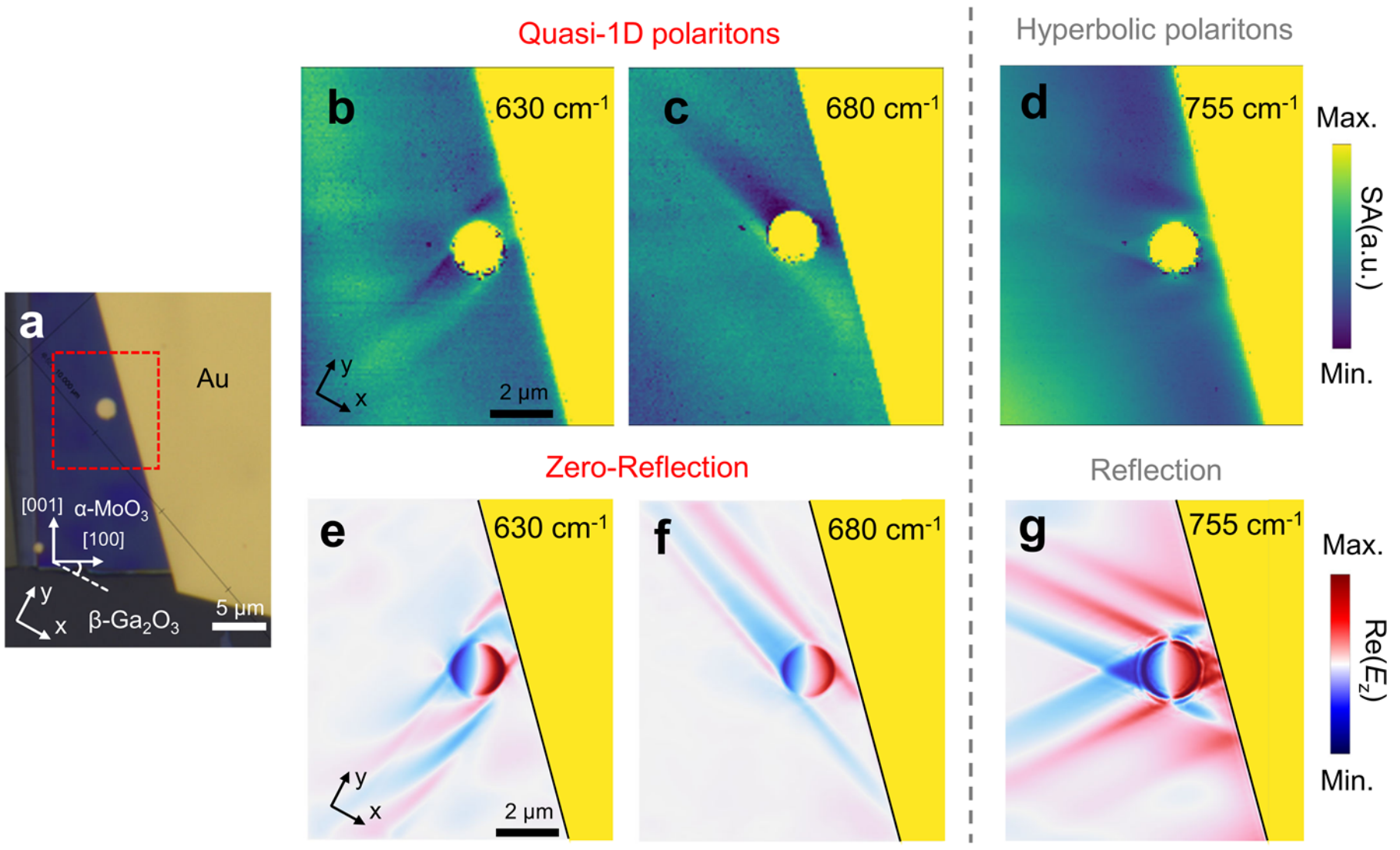


**Fig. S21: The near-field images of quasi-1D polaritons with gold mirror.**

**a,** Microscopic image of the measured α-$MoO_3$ flake/β-$Ga_2O_3$ bulk heterostructure with a twist angle θ of 26°, featuring a central gold disc as the excitation source. A single crystal of gold was placed beside the gold disc as a mirror. The thickness of the thinner α-$MoO_3$ is about 120 nm. The twist angle is 26°. **b-d**, The experimental results of ultra-broad-angle zero-reflection of quasi-1D polaritons **(b,c)** and reflection of hyperbolic polaritons of the heterostructure (measured in the red dashed box in **a**). **e-g**, Simulation corresponding to **b-d**. By tuning the operating frequency to 755 $cm^{-1}$, where the permittivities along the three principal axes of β-$Ga_2O_3$ are all positive (i.e., behaving as an ordinary dielectric), clear reflection of hyperbolic polaritons of α-$MoO_3$ was observed (**d, g**).

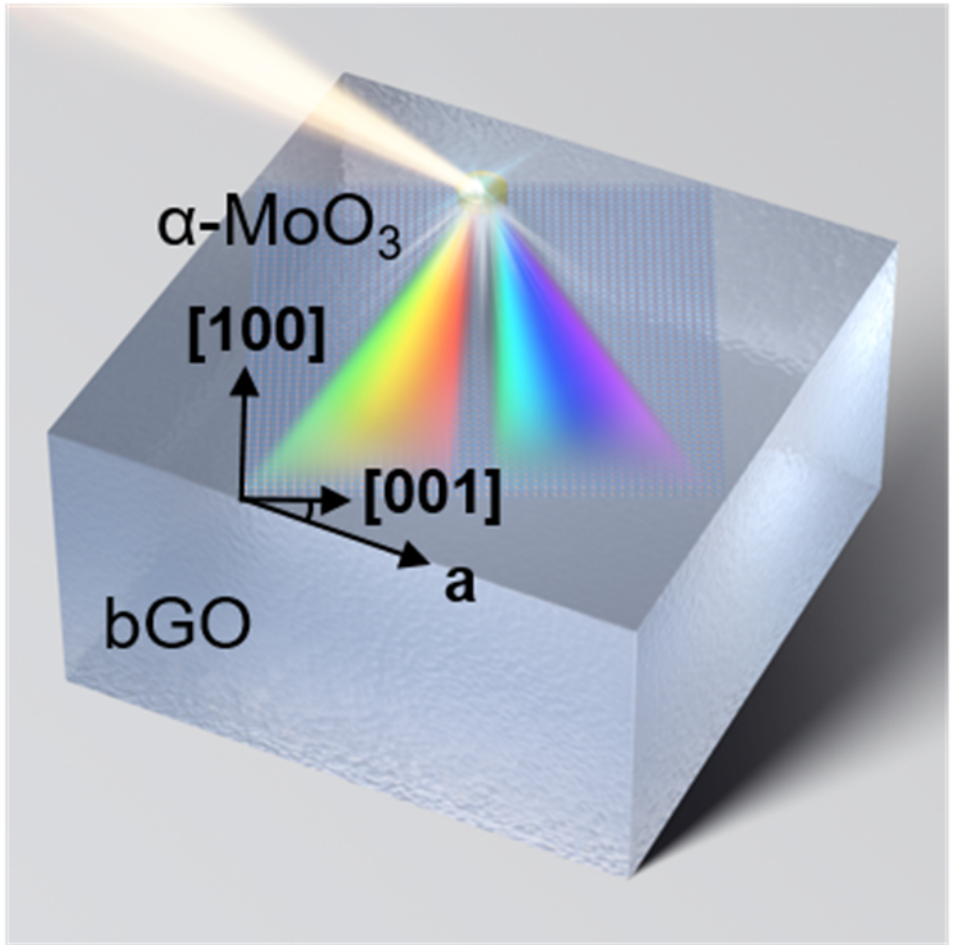


**Fig. S22: Dual-Band Polariton Rainbow Router**

Schematic of dual-band polaritonic rainbow routing, illustrating how quasi-1D polaritons are directed to different locations depending on frequency. Distinct colors represent different frequencies; each rainbow corresponds to a separate frequency band.

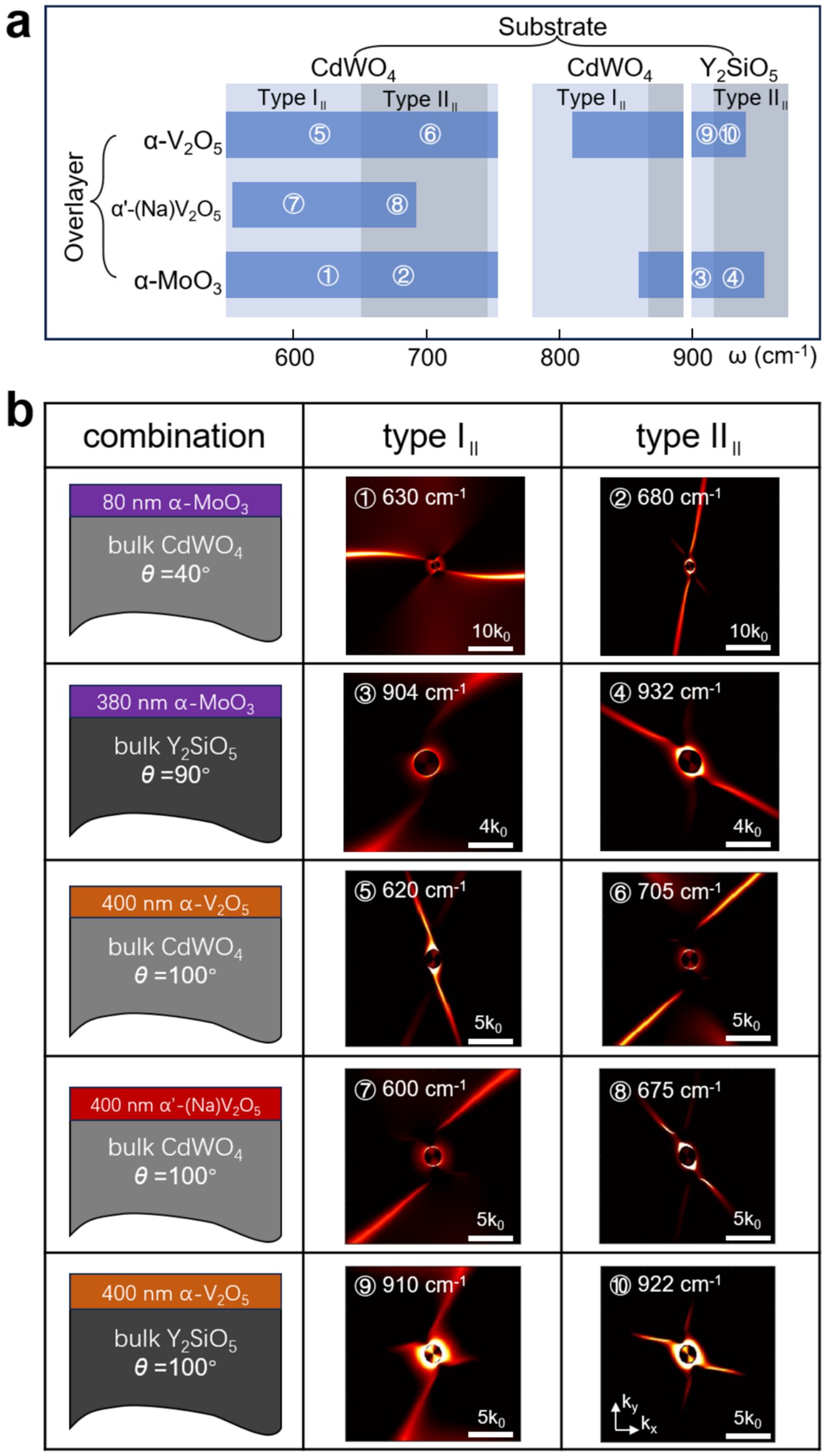


**Fig. S23: Iso-frequency contours of other material pairs supporting quasi-1D polaritons**

The examples in **b** demonstrate the switch of quasi-1D polariton due to the type-I-to-II change of the substrate's in-plane hyperbolic polariton mode. They further illustrate the 1D polariton switching situations of other material combinations mentioned in **a** by corresponding circled number, demonstrating the wide-bandwidth tunability of our mechanism. The twist angles θ and wave numbers for each case have been marked in the figure.

**References:**


1. Álvarez-Cuervo, J. *et al.* Unidirectional ray polaritons in twisted asymmetric stacks. *Nat Commun* **15**, 9042 (2024).
2. Dai, S. *et al.* Tunable Phonon Polaritons in Atomically Thin van der Waals Crystals of Boron Nitride. *Science* **343**, 1125–1129 (2014).
3. Zheng, Z. *et al.* A mid-infrared biaxial hyperbolic van der Waals crystal. *Sci. Adv.* **5**, eaav8690 (2019).
4. Schubert, M. *et al.* Anisotropy, phonon modes, and free charge carrier parameters in monoclinic β -gallium oxide single crystals. *Phys. Rev. B* **93**, 125209 (2016).
5. Hu, G. *et al.* Real-space nanoimaging of hyperbolic shear polaritons in a monoclinic crystal. *Nat. Nanotechnol.* **18**, 64–70 (2023).
6. Zheng, C. *et al.* Hyperbolic-to-hyperbolic transition at exceptional Reststrahlen point in rare-earth oxyorthosilicates. *Nat Commun* **15**, 7047 (2024).
7. Taboada-Gutiérrez, J. *et al.* Broad spectral tuning of ultra-low-loss polaritons in a van der Waals crystal by intercalation. *Nature Materials* **19**, 964–968 (2020).
8. Caldwell, J. D. *et al.* Sub-diffractional volume-confined polaritons in the natural hyperbolic material hexagonal boron nitride. *Nat Commun* **5**, 5221 (2014).
9. Passler, N. C. & Paarmann, A. Generalized 4 × 4 matrix formalism for light propagation in anisotropic stratified media: study of surface phonon polaritons in polar dielectric heterostructures. *J. Opt. Soc. Am. B* **34**, 2128 (2017).
10. Passler, N. C. *et al.* Hyperbolic shear polaritons in low-symmetry crystals. *Nature* **602**, 595–600 (2022).
11. Matson, J. *et al.* Controlling the propagation asymmetry of hyperbolic shear polaritons in beta-gallium oxide. *Nat Commun* **14**, 5240 (2023).
12. Menabde, S. G. *et al.* Near-field probing of image phonon-polaritons in hexagonal boron nitride on gold crystals. *Sci. Adv.* **8**, eabn0627 (2022).
13. Ma, W. *et al.* Ghost hyperbolic surface polaritons in bulk anisotropic crystals. *Nature* **596**, 362–366 (2021).
