# Supplementary material for: Loss-engineered reconfigurable polaritons
Source: Nat Commun. 2026 Sep 9;17:10658. doi: 10.1038/s41467-026-77412-4 (PMC13654931; doi:10.1038/s41467-026-77412-4)
Supplement: Supplementary file 2 [file 41467_2026_77412_MOESM2_ESM.pdf]

nature portfolio

Peer Review File

Loss-Engineered Reconfigurable Polaritons

Corresponding Author: Professor Qing Dai

This file contains all reviewer reports in order by version, followed by all author rebuttals in order by version.

Version 0:

Reviewer comments:

Reviewer #1

(Remarks to the Author)
This study proposes a novel mechanism for manipulating phonon polaritons by harnessing "leaky loss". By introducing out-of-plane phonon-driven leakage channels in heterostructures such as two-dimensional α-MoO_3 and three-dimensional β-Ga_2O_3, the authors achieve topological reconstruction of quasi-one-dimensional polaritons, frequency-dependent directional rotation, and wide-angle reflectionless transmission. The research concept is innovative, and the combination of experimental and theoretical approaches is relatively robust, demonstrating a new class of polariton control strategies. The overall quality of the work is acceptable, yet it can be further improved in terms of mechanistic elucidation, data presentation, and universality validation. I recommend that the manuscript be accepted after proper revisions.

1. Lines 53–56: The classification of optical losses is ambiguous. The statement "optical losses mainly consist of intrinsic losses caused by material phonon and radiative losses resulting from leakage into external media" requires clarification. (1) Do optical losses not include scattering losses? (2) Are intrinsic losses entirely attributed to material phonons? Does "material phonon" specifically refer to phonon absorption? The term "material phonon" is also vague. (3) Can radiative losses be regarded as extrinsic losses in contrast to intrinsic losses? The authors should clarify this classification with appropriate literature support or specify the research context for rigor.

2. The term "quasi-1D" is introduced without a clear definition. The authors should provide a more specific explanation, for example, in terms of the angular divergence of propagation directions or the curvature of the isofrequency contour.

3. Lines 82–85: The authors introduce "loss engineering" as a core innovation and use terms such as "phonon-switch leaky loss mechanism" without a prior definition. I recommend briefly defining "loss engineering" and its design principles at the beginning of the Results section to improve readability and logical flow.

4. Regarding the universality of loss engineering: The manuscript claims a universal mechanism. Does this mechanism rely solely on monoclinic crystals? Is it dependent on the specific crystal direction? For non-monoclinic crystals, could a similar quasi-1D polariton behavior be achieved through interface cutting (e.g., in $CaCO_3$)? The authors should discuss the scope and limitations of the proposed strategy.

5. Influence of incident light direction: previous studies reported that varying the incident light direction significantly affects polariton propagation and field intensity in β-Ga_2O_3. Does the quasi-1D polariton in β-Ga_2O_3 proposed in this work exhibit similar sensitivity? This should be addressed.

Additional technical comments:

1. Abbreviations such as "2D," "1D," and "vdW" are used inconsistently throughout the text (e.g., Lines 60–79). Please perform a full check and unify their usage.
2. The reason for selecting a twist angle of 26° is not explained. A brief justification should be added.
3. Figure 1a: The crystal structures shown are not clearly identified. If they are not essential to the main message, consider removing them. Additionally, the label "quasi-1D polariton" appears; adding a corresponding "hyperbolic polariton" label may help illustrate the transition from hyperbolic to quasi-1D behavior.
4. Figure 1b: The gray "×" marks are not explained. Please add a description in the caption.
5. Figures 3b and 3c: The annotations for k and S in momentum space are clearly indicated. If corresponding real-space images with Fourier transforms can be obtained, please add annotations for consistency.

6. Figure 4a: Different atoms (e.g., W and Mo) should preferably be distinguished using distinct colors. The labeled arrows a, b, and c should also be explained in the caption.
7. Figure 4c and broadband coverage: The text claims $\Delta\lambda \sim 7$ μm, but Figure 4c shows two separated bands (10.4–12.3 μm and 13.3–18.2 μm) with a gap. The authors should clarify whether "broadband" refers to the total coverage range or a single continuous band.

Reviewer #2

(Remarks to the Author)
The manuscript by Hongyi Yuan et al. proposes a loss-engineering strategy that harnesses optical leakage loss as a degree of freedom for modulation, aiming to achieve reconfigurable control of quasi-1D polaritons and ultra-broad-angle reflectionless transmission in 2D/3D heterostructures. The approach is interesting.
However, the experimental data currently presented in the manuscript seems do not sufficiently substantiate the claimed ultra-broad-angle reflectionless transmission and the universality of the heterostructure platform. Furthermore, the interpretation of the leakage mode mechanism lacks sufficient detail. Therefore, I cannot recommend publication of this manuscript in its present form, although the results may become suitable for Nature Communications after the following issues are clarified and improved.
1. What is the propagation length of quasi-1D polaritons at different frequencies, and how is the loss quantitatively evaluated? Furthermore, what are the respective influences of substrate loss and flake thickness on the propagation length?
2. The frequency-dependent rotation of quasi-1D polaritons is only qualitatively shown. A quantitative analysis (e.g., rotation angle vs. frequency) is needed. Is this rotation linear or nonlinear, and does it arise from intrinsic material dispersion or heterostructure coupling? More importantly, does the substrate material itself independently influence the rotation angle beyond serving as a leaky channel?
3. What is the pruning efficiency of the leakage mechanism—can specific hyperbolic branches be completely suppressed, and under what physical conditions? Additionally, what is the polariton propagation behavior exactly at the critical frequency of 659 cm^{-1}, where the topological transition occurs? Does the quasi-1D character persist at this singular point, or does the mode exhibit fundamentally different behavior?
4. The near-field images at step boundaries (Fig. 3d and Fig. S15) show relatively low signal-to-noise ratio, which may affect the reliability of the claimed zero-reflection observation. The authors should discuss the origin of this noise and whether it limits the angular or frequency range over which zero reflection can be confidently asserted. Additionally, I wonder whether the effect remains equally valid when polaritons propagate from the thicker region to the thinner region compared to the opposite direction, or if asymmetry in the step geometry introduces directional dependence.
5. The near-field image in Fig. 4b (α-MoO_3/$CdWO_4$ heterostructure) exhibits noticeably lower signal-to-noise ratio compared to Fig. 2b, particularly in the right panel where the propagation pattern appears more characteristic of hyperbolic spreading rather than well-confined quasi-1D rays. The authors should explain the reason.

Version 1:

Reviewer comments:

Reviewer #1

(Remarks to the Author)
All the previous concerns have been fully addressed and the revised manuscript has improved significantly in terms of both scientific rigor and data presentation. I recommend acceptance now.

Reviewer #2

(Remarks to the Author)
The authors have addressed most of my technical concerns, and the manuscript has been substantially improved. Therefore, I would recommend the manuscript to be published on Nature Communications.

Open Access This Peer Review File is licensed under a Creative Commons Attribution 4.0 International License, which permits use, sharing, adaptation, distribution and reproduction in any medium or format, as long as you give appropriate credit to the original author(s) and the source, provide a link to the Creative Commons license, and indicate if changes were made.
In cases where reviewers are anonymous, credit should be given to 'Anonymous Referee' and the source.
The images or other third party material in this Peer Review File are included in the article's Creative Commons license, unless indicated otherwise in a credit line to the material. If material is not included in the article's Creative Commons license and your intended use is not permitted by statutory regulation or exceeds the permitted use, you will need to obtain permission directly from the copyright holder.
To view a copy of this license, visit https://creativecommons.org/licenses/by/4.0/

RESPONSE TO REVIEWERS' COMMENTS

We thank the reviewers for the constructive and insightful comments on our manuscript. These suggestions have been invaluable in helping us further improve the quality and clarity of our work. We have carefully addressed all points raised by the reviewers and provide a detailed point-by-point response below. We believe the revised manuscript has been substantially strengthened as a result. Alongside this resubmission, we have enclosed the revised manuscript and supplementary information.

Best regards,

The authors

****

Our point-by-point response

Reviewer #1

Comment: This study proposes a novel mechanism for manipulating phonon polaritons by harnessing "leaky loss". By introducing out-of-plane phonon-driven leakage channels in heterostructures such as two-dimensional α-MoO_3 and three-dimensional β-Ga_2O_3, the authors achieve topological reconstruction of quasi-one-dimensional polaritons, frequency-dependent directional rotation, and wide-angle reflectionless transmission. The research concept is innovative, and the combination of experimental and theoretical approaches is relatively robust, demonstrating a new class of polariton control strategies. The overall quality of the work is acceptable, yet it can be further improved in terms of mechanistic elucidation, data presentation, and universality validation. I recommend that the manuscript be accepted after proper revisions.

Reply: We sincerely thank the reviewer for the thorough and constructive evaluation of our manuscript, and for recognizing the novelty of our work and its publication potential. We have carefully addressed all comments and revised the manuscript accordingly. Detailed point-by-point responses are provided below.

1. Lines 53–56: The classification of optical losses is ambiguous. The statement “optical losses mainly consist of intrinsic losses caused by material phonon and radiative losses resulting from leakage into external media” requires clarification. (1) Do optical losses not include scattering losses? (2) Are intrinsic losses entirely attributed to material phonons? Does “material phonon” specifically refer to phonon absorption? The term “material phonon” is also vague. (3) Can radiative losses be regarded as extrinsic losses in contrast to intrinsic losses? The authors should clarify this classification with appropriate literature support or specify the research context for rigor.

Reply: We thank the Reviewer for this insightful comment regarding the classification of optical losses. The optical loss of phonon polaritons propagating in high-quality thin films can generally be divided into two categories: intrinsic material loss (i.e., phonon absorption) and extrinsic loss (Ref. *Nat. Commun.* 2020, 11, 3649). We previously referred to extrinsic loss collectively as radiative loss, which may cause ambiguity. In practice, extrinsic loss can be further divided into two main components: scattering loss due to the radiation of polariton electromagnetic energy into air, and leakage loss due to radiative coupling of polariton electromagnetic energy into the substrate. In response to the three questions raised by the reviewers, which concern (1) the presence of scattering loss, (2) the revision of "material phonon" to "phonon absorption," and (3) the clarification that radiative loss is an extrinsic loss, we have carefully addressed each point.

Changes made in the revised manuscript and supplementary information:

We thank the reviewer for this question. To improve clarity and accuracy in our manuscript, we have made the following changes in the revised manuscript, as marked in blue:

In lines 52-59 on Page 2:

Text before revision:

"While reduced crystal symmetry broadens the available polariton types and operating bands, performance in functional device designs has long been limited by optical losses.

Generally, optical losses mainly consist of intrinsic losses caused by material phonon and radiative losses resulting from leakage into external media, which are considered as the principal bottleneck hindering the practical realization of phonon polariton-based devices."

Text after revision:

"While reduced crystal symmetry broadens the available polariton types and operating bands, performance in functional device designs has long been limited by optical losses, which are considered as the principal bottleneck hindering the practical realization of phonon polariton-based devices. Generally, the optical loss of phonon polaritons propagating in high-quality thin films comprises two categories: intrinsic material loss (mainly phonon absorption[20]) and extrinsic loss[21]. The latter mainly consists of two components: scattering loss from radiation of polariton energy into the ambient, and leakage loss from radiative coupling into the substrate"

In lines 419-422 on Page 14:

"20. Zhong, G. *et al.* Low-temperature hyperbolic phonon polaritonics with suppressed phononic scattering. *Science Advances* **12**, eaef2802 (2026).

21. Lee, I.-H. *et al.* Image polaritons in boron nitride for extreme polariton confinement with low losses. *Nat Commun* **11**, 3649 (2020)."

2. The term "quasi-1D" is introduced without a clear definition. The authors should provide a more specific explanation, for example, in terms of the angular divergence of propagation directions or the curvature of the isofrequency contour.

Reply: We thank the reviewer for this valuable suggestion. In the revised manuscript, we have provided a clearer definition of the concept of "quasi-1D" as follows.

First, from the perspectives of angular divergence and transverse confinement, we performed a quantitative analysis of the $|\mathbf{E}_z|$ field distribution under a representative configuration in Fig. R1 (Ref. *Nat. Commun.*, 2024, 15, 9042). The concentric field pattern centered at the excitation source shows that, as the propagation length increases, the peak angular position remains unchanged, whereas the angular full width at half maximum (FWHM) gradually decreases. Meanwhile, the transverse FWHM of the field

profile along the propagation direction remains nearly constant. These results confirm that the mode exhibits both high collimation and strong transverse confinement.

Second, from the perspective of the underlying physical mechanism, namely the characteristics of the isofrequency contour, this behavior originates from the fact that the energy flow represented by the Poynting vector is nearly orthogonal to the wavevector across the entire field pattern. In other words, the group velocity is nearly orthogonal to the phase velocity, giving rise to highly directional, ray-like propagation.

In summary, based on this strongly collimated energy transport and the propagation symmetry along a single direction, we formally refer to this excitation mode as the "quasi-1D" polariton.

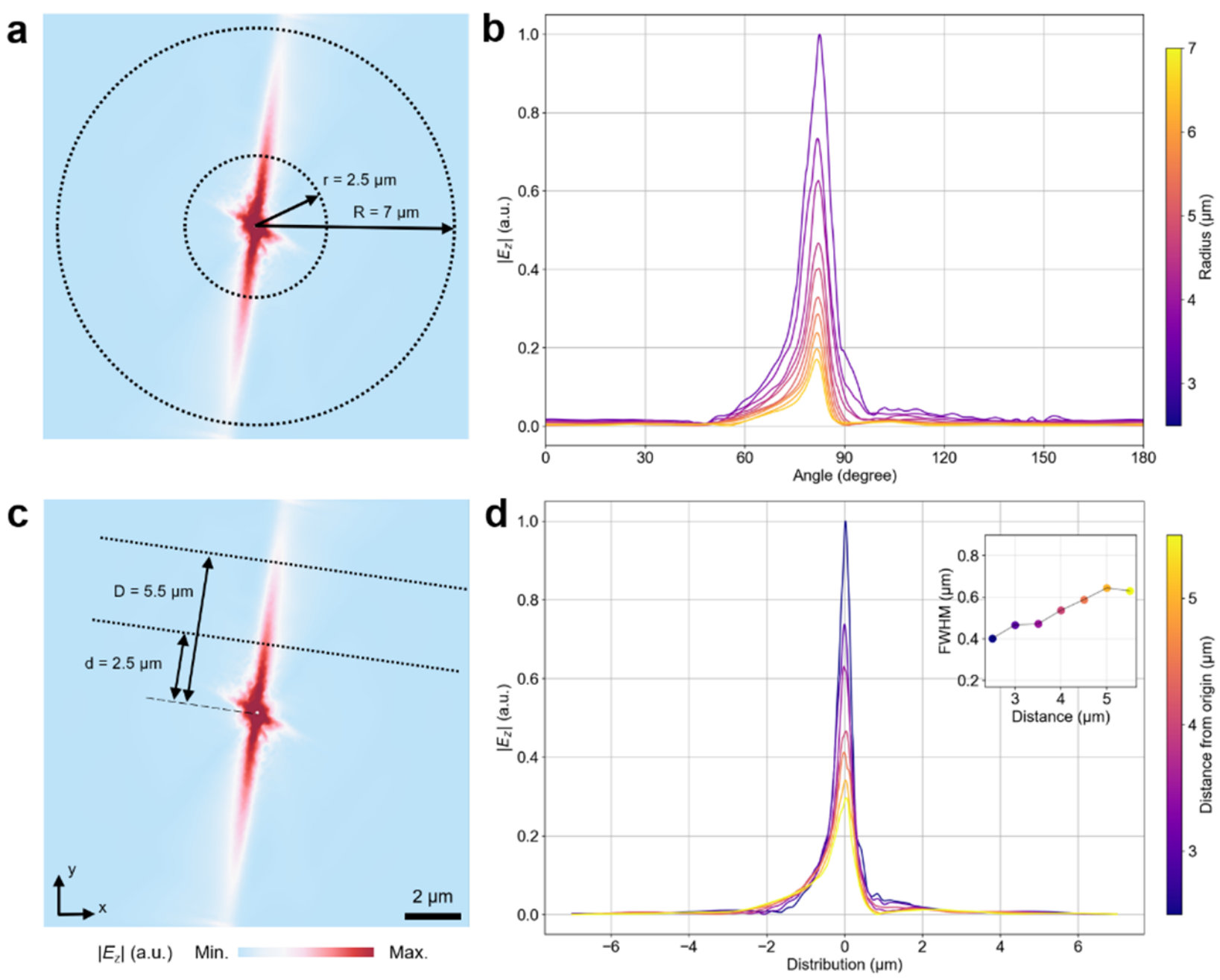

Fig. R1 Analysis of real space distribution of quasi-1D polaritons. a, **c**, Two types of propagation analysis of electric field component |**E**$_z$| of 630 cm^{-1} generated by a point dipole of a 400 nm α-MoO_3/ bulk β-Ga_2O_3 heterostructure from simulation. The twist angle is 26°. **b**, Profiles of the simulations along circular cuts (black dotted circles in **a**) from 2.5-7 μm far from the dipole source. **d**, Profiles of the transverse field along the propagation direction. The data were extracted from a group of black dotted lines shown in **c**, which are located at a distance of 2.5-5.5 μm from the dipole source. The propagation direction defined by the peak angular positions identified in **b**. The FWHM of the transverse field profile of different distance from the dipole source are displayed in the

illustration in corresponding colors.

Changes made in the revised manuscript and supplementary information:

We thank the reviewer for this question, in response to which we have made the following changes in lines 88-89 on Page 3 of the revised manuscript, as marked in blue:

"...quasi-1D polaritons (see Supplementary Note 1 and Fig. S1 for its definition)."

We have also added Note 1 and Fig. R1 as Fig. S1 in the revised supplementary information, as marked in blue:

"**Note 1: Definition of quasi-1D polaritons**

Here, we discuss the definition of the concept of quasi-one-dimensional (quasi-1D) polaritons from the mode distribution and physical mechanism.

First, from the perspectives of angular divergence and transverse confinement, we performed a quantitative analysis of the $|\mathbf{E}_z|$ field distribution under a representative configuration in Fig. S1 [1]. The concentric field pattern centered at the excitation source shows that, as the propagation length increases, the peak angular position remains unchanged, whereas the angular full width at half maximum (FWHM) gradually decreases. Meanwhile, the transverse FWHM of the field profile along the propagation direction remains nearly constant. These results confirm that the mode exhibits both high collimation and strong transverse confinement.

Second, from the perspective of the underlying physical mechanism, namely the characteristics of the isofrequency contour, this behavior originates from the fact that the energy flow represented by the Poynting vector is nearly orthogonal to the wavevector across the entire field pattern. In other words, the group velocity is nearly orthogonal to the phase velocity, giving rise to highly directional, ray-like propagation.

In summary, based on this strongly collimated energy transport and the propagation symmetry along a single direction, we formally refer to this excitation mode as the "quasi-1D" polariton."

" **Fig. S1: Analysis of real space distribution of quasi-1D polaritons.**

a, **c**, Two types of propagation analysis of electric field component $|\mathbf{E}_z|$ of 630 cm^{-1}

generated by a point dipole of a 400 nm α-MoO_3/ bulk β-Ga_2O_3 heterostructure from simulations. The twist angle is 26°. **b**, Profiles of the simulations along circular cuts (black dotted circles in **a**) from 2.5-7 μm far from the dipole source. **d**, Profiles of the transverse field along the propagation direction. The data were extracted from a group of black dotted lines shown in **c**, which are located at a distance of 2.5-5.5 μm from the dipole source. The propagation direction is defined by the peak angular positions identified in panel **b**. The FWHM of the transverse field profile of different distance from the dipole source are displayed in the illustration in corresponding colors."

3. Lines 82–85: The authors introduce "loss engineering" as a core innovation and use terms such as "phonon-switch leaky loss mechanism" without a prior definition. I recommend briefly defining "loss engineering" and its design principles at the beginning of the Results section to improve readability and logical flow.
Reply: We thank the reviewer for this suggestion. We have added a clear definition of "loss engineering" and its design principles in the revised manuscript. In our work, loss engineering refers to leveraging optical losses to tailor the isofrequency contours, near-field distributions, and mode characteristics of polaritons, thereby turning loss into an effective tool for polariton modulation (Ref. *Nat. Commun.* 2024 15, 2696 and Ref. *Nature* 2022, 602, 595). Optical losses generally comprise intrinsic material absorption losses, optical scattering losses, and leakage losses. Conventionally, optical losses have been regarded as a detrimental factor in polariton propagation and have rarely been exploited as a means of functional modulation. To address this limitation, we introduce in the present work a phonon-induced, switchable leakage-loss mechanism as a tunable loss-engineering platform: a bulk substrate provides a continuous wavevector-matching condition that triggers angle-selective radiative leakage of phonon polaritons from the upper layer into the substrate, thereby enabling the on-demand conversion of mirror-symmetric hyperbolic polaritons into quasi-1D propagating states.

Changes made in the revised manuscript and supplementary information:

We thank the reviewer for this question. To improve clarity and accuracy in our manuscript, we have made the following changes in lines 84-89 on Page 3 of the revised manuscript, as marked in blue:

Text before revision:

"Reconfiguring polariton mechanisms via phonon-switched leaky loss

To demonstrate how the proposed phonon-switch leaky loss mechanism sculpts hyperbolic waveguide modes into switchable quasi-1D polaritons, Fig. 1a presents two perpendicular cross-sections illustrating the differences in propagation directions of the hyperbolic polariton waveguide mode. "

Text after revision:

"Reconfiguring polariton mechanisms via phonon-switched leaky loss

Loss engineering refers to leveraging optical losses to tailor the isofrequency contours, near-field distributions, and mode characteristics of polaritons, thereby turning loss into an effective tool for polariton modulation. Building upon this concept, we introduce a phonon-switch leaky loss mechanism that sculpts hyperbolic waveguide modes into switchable quasi-1D polaritons (see Supplementary Note 1 and Fig. S1 for its definition). To explain the proposed phonon-switch leaky loss mechanism, Fig. 1a presents two perpendicular cross-sections illustrating the differences in propagation directions of the hyperbolic polariton waveguide mode."

4. Regarding the universality of loss engineering: The manuscript claims a universal mechanism. Does this mechanism rely solely on monoclinic crystals? Is it dependent on the specific crystal direction? For non-monoclinic crystals, could a similar quasi-1D polariton behavior be achieved through interface cutting (e.g., in $CaCO_3$)? The authors should discuss the scope and limitations of the proposed strategy.

Reply: We thank the reviewer for this question. Following the suggestion, we have added a discussion of the applicability and limitations of this strategy in the revised manuscript.

This mechanism is not limited to monoclinic crystals. The core conditions for the formation of quasi-one-dimensional polaritons are that both the 2D layered film and the

3D bulk substrate support in-plane hyperbolic isofrequency contours within the same frequency range and that continuous wavevector matching is achieved over a suitable twist-angle range, thereby producing angle-selective leakage loss. Anisotropic materials of various symmetries, including orthorhombic, trigonal, and monoclinic systems, can all satisfy these conditions. Taking calcite ($CaCO_3$), a trigonal crystal mentioned by the reviewer, as an example, it can serve as a bulk substrate supporting quasi-1D polaritons when paired with a film possessing a matching hyperbolic frequency band, regardless of whether an interface cut is employed. As shown in Fig. R2, we performed theoretical simulations using 3D $CaCO_3$ with different crystallographic cuts as the substrate, topped by a 2D van der Waals material with artificially prescribed hyperbolic dielectric parameters; all configurations exhibit quasi-1D polariton characteristics.

Regarding limitations, this strategy requires constructing a heterostructure in which the two-dimensional layered material and the three-dimensional substrate share a common hyperbolic frequency window. Identifying suitable material combinations is challenging, primarily because the available frequency bands are dictated by the discrete TO–LO phonon resonance frequencies intrinsic to each crystal. As noted in the manuscript, monoclinic crystals, owing to their reduced symmetry, possess richer phonon modes and consequently a broader frequency range available for matching, making them particularly favorable but not the only candidates. We have expanded the discussion in the revised manuscript to address the scope of application and limitations of the proposed strategy.

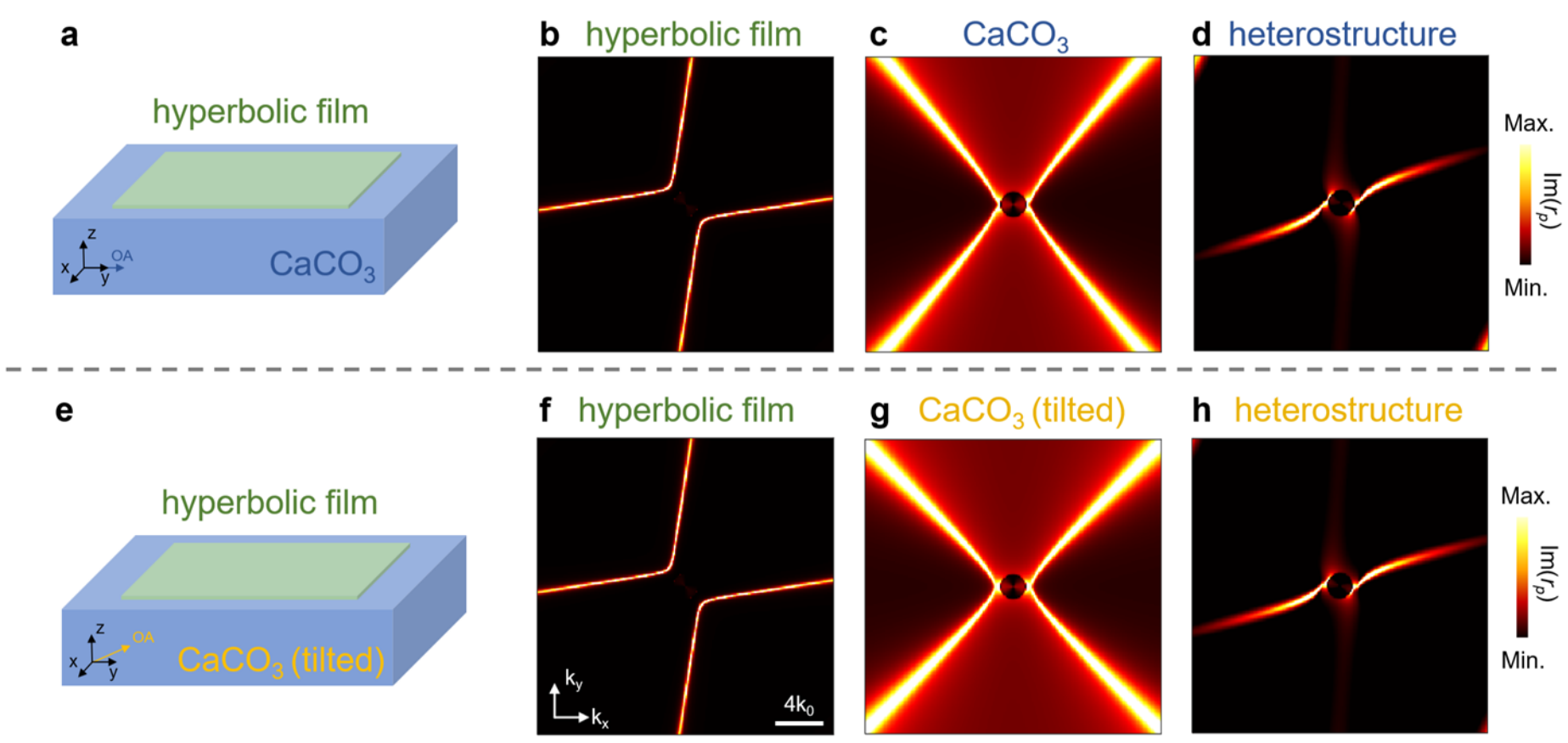

Fig. R2 Theoretical simulations with $CaCO_3$. a, e, Schematic diagram of two heterostructures. The optical axis (OA) orientation differs between the two rows: in panels (**a**)–(**d**), the calcite OA lies parallel to the (100) surface (0° tilt), whereas in panels (**e**)–(**h**), the calcite OA is tilted at 23.3° relative to the (100) surface. **b-d, f-h**, IFCs of (**b**, **f**) a 200 nm-thick artificial hyperbolic film (ε_x = 8.1455+0.0404i, ε_y = -14.5217 + 0.4485i, ε_z = 2.8262 + 0.0011i, twist angle = 45°), (**c**, **g**) bulk calcite ($\varepsilon_{//}$ = 2.34, $\varepsilon_{\perp}$ = 3.74 + 0.55i, which is the permittivity of calcite at ω = 1470 cm^{-1} (Ref. *Nature* 2021, 596, 362)), and (**d**, **h**) their heterostructure. The permittivity of the artificial hyperbolic film is taken from the permittivity of α-MoO_3 at ω = 640 cm^{-1}.

Changes made in the revised manuscript and supplementary information:

We thank the reviewer for this question, in response to which we have made the following changes in the revised manuscript, as marked in blue:

In lines 245-249 on Page 9:

"Theoretical calculations reveal that quasi-1D polaritons can be formed when the hyperbolic waveguide modes in the thin cladding layer overlap with the hyperbolic bulk modes served as leaky channels in the bulk substrate. Accordingly, materials capable of supporting in-plane hyperbolic bulk polaritons across diverse crystal systems, such as monoclinic β-Ga_2O_3 or trigonal $CaCO_3$, may all be regarded as viable candidates."

In lines 251-258 on Page 9:

"However, this mechanism is limited by the frequency response matching between the cladding and the substrate. If a frequency-matching material combination cannot be found, this mode cannot be realized. For example, when employing $CaCO_3$ with

hyperbolic bulk modes as the leakage substrate, it is currently very difficult to find a suitable cladding material that both matches the frequency and supports hyperbolic waveguide modes. Consequently, we are unable to conduct relevant experiments based on the $CaCO_3$ material system.

Furthermore, controlling the propagation......"

5. Influence of incident light direction: previous studies reported that varying the incident light direction significantly affects polariton propagation and field intensity in β-Ga_2O_3. Does the quasi-1D polariton in β-Ga_2O_3 proposed in this work exhibit similar sensitivity? This should be addressed.

Reply: Thanks for the reviewer's valuable question. The direction of incident light indeed affects the propagation characteristics and field strength of the quasi-1D polaritons, mainly by changing their excitation efficiency, but does not impact their quasi-1D features. Specifically, the incident light direction determines the excitation efficiency of polaritons through the adjustment of polarization. Previous studies (Ref. *Nat. Commun.* 2023, 14, 5240) have shown that incident light on the gold disc can be regarded as a dipole polarized in the plane of incidence, and modes perpendicular to the polarization direction are most efficiently excited. Our experimental results are consistent with this observation.

In the α-MoO_3/β-Ga_2O_3 heterostructure, we systematically measured and simulated the behavior of quasi-1D polaritons at 680 cm^{-1} under different incident light directions. As illustrated in Fig. R3, when the incident light direction was rotated by 45° increments, the propagation length and field distribution of the quasi-1D polaritons changed correspondingly: when the incident light was almost perpendicular to the polariton direction (Fig. R3a and R3e), the propagation length was longer and the field distribution clearer; in contrast, when the light was nearly parallel (Fig. R3c and R3h), the propagation length was reduced and the field intensity weakened. In summary, quasi-1D polaritons can be excited at all incident angles, although the excitation efficiency varies. Their directional propagation and anisotropy primarily originate from the heterostructure itself, and they show good robustness against changes in incident

direction.

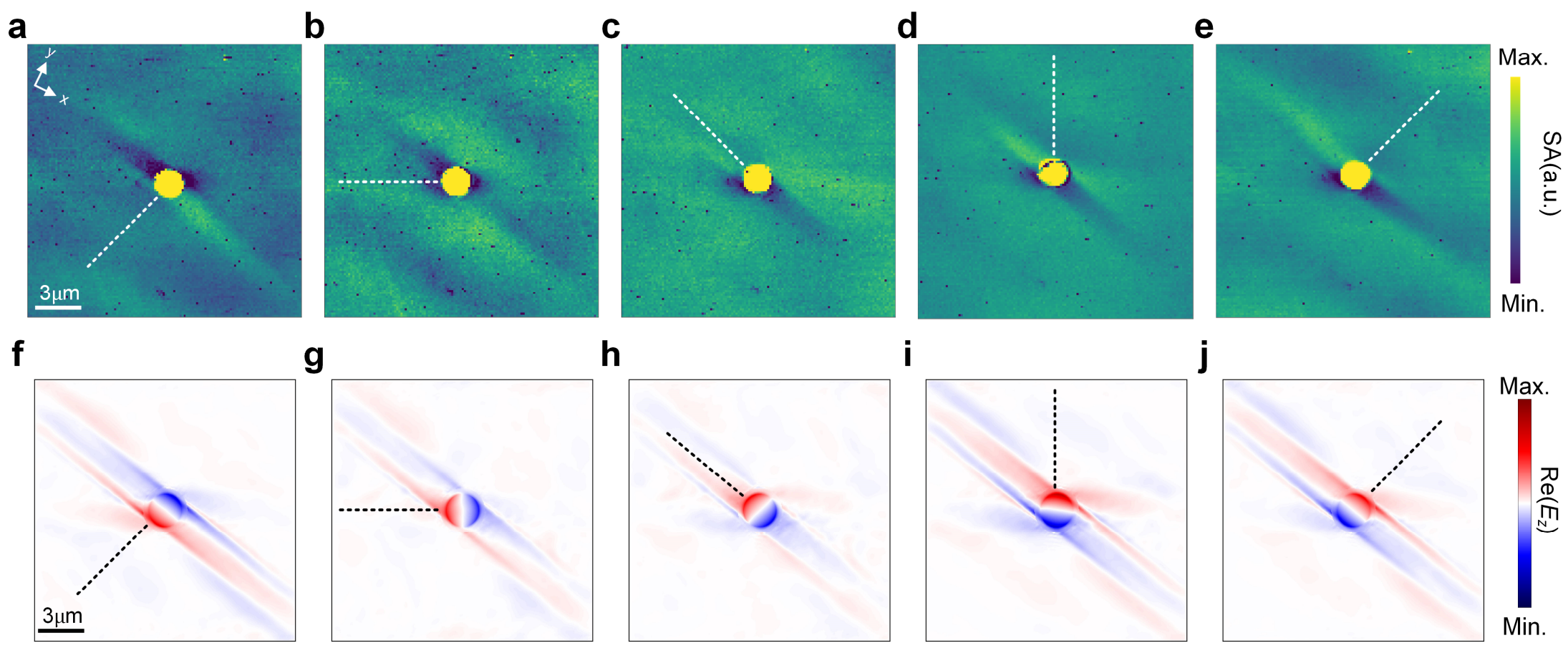

Fig. R3 The dependence on illumination orientation of quasi-1D polaritons of α-MoO_3/β-Ga_2O_3 heterostructure. **a-e,** Experimental near-field images of quasi-1D polaritons, excited by a 2.5 μm gold disk at the frequency of 680 cm^{-1}. The white dashed line illustrates the illumination direction of the input light, which rotates in 45-degree steps. The thickness of α-MoO_3 is 100 nm and the twist angle is 26°. **f-j**, Simulation of quasi-1D polaritons propagation with conditions according to (**a**–**e**).

Changes made in the revised manuscript and supplementary information:

We thank the reviewer for this question, in response to which we have made the following changes in lines 193-195 on Page 7 of the revised manuscript, as marked in blue:

"Further studies demonstrate that the propagation characteristics of these quasi-1D polaritons are robust against variations in both the incident direction of the excitation light (Supplementary Note 12 and Fig. S19) and the α-MoO_3 flake thickness (Fig. S20)"

We have also added Note 12 and Fig. R3 as Fig. S19 in the revised supplementary information, as marked in blue:

"Note 12: The effects of illumination orientation to quasi-1D polaritons

The direction of incident light indeed affects the propagation characteristics and field strength of the quasi-1D polaritons, mainly by changing their excitation efficiency, but does not impact their quasi-1D features. Specifically, the incident light direction determines the excitation efficiency of polaritons through the adjustment of polarization.

Previous studies [11] have shown that incident light on the gold disk can be regarded as a dipole polarized in the plane of incidence, and modes perpendicular to the polarization direction are most efficiently excited. Our experimental results are consistent with this observation.

In the α-MoO_3/β-Ga_2O_3 heterostructure, we systematically measured and simulated the behavior of quasi-1D polaritons at 680 cm^{-1} under different incident light directions. As illustrated in Fig. S19, when the incident light direction was rotated by 45° increments, the propagation length and field distribution of the quasi-1D polaritons changed correspondingly: when the incident light was almost perpendicular to the polariton direction (Fig. S19a and S19e), the propagation length was longer and the field distribution clearer; in contrast, when the light was nearly parallel (Fig. S19c and S19h), the propagation length was reduced and the field intensity weakened. In summary, quasi-1D polaritons can be excited at all incident angles, although the excitation efficiency varies. Their topological properties and anisotropy primarily originate from the heterostructure itself, and they show good robustness against changes in incident direction."

"Fig. S19: The dependence on illumination orientation of quasi-1D polaritons.

a-e, Experimental near-field images of quasi-1D polaritons, excited by a 2.5 μm gold disk at the frequency of 680 cm^{-1}. The white dashed line illustrates the illumination direction of the input light, which rotates in 45-degree steps. The thickness of α-MoO_3 is 100 nm and the twist angle is 26°. **f-j**, Simulation of quasi-1D polaritons propagation with conditions according to (**a**–**e**)."

6. Abbreviations such as "2D," "1D," and "vdW" are used inconsistently throughout the text (e.g., Lines 60–79). Please perform a full check and unify their usage.

Reply: We thank the reviewer for this suggestion. We have conducted a comprehensive review of all the relevant abbreviations, ensuring the uniformity and accuracy of the entire text.

Changes made in the revised manuscript and supplementary information:

We thank the reviewer for this question, in response to which we have made the following changes in the revised manuscript, as marked in blue:

In line 48 on Page 2: add "(2D)"

In lines 65 and 67 on Page 2: " two-dimensional (2D)" is changed into "2D"

In line 77 on Page 3: "van der Waals" is changed into "van der Waals (vdW)"

In line 127 on Page 4: "van der Waals (vdW)" is changed into "vdW"

7. The reason for selecting a twist angle of 26° is not explained. A brief justification should be added.

Reply: We thank the reviewer for this suggestion. The quasi-1D polaritons in α-MoO_3/β-Ga_2O_3 can form over a broad twist-angle range (approximately −50° to 70°), which is shown in Fig. R4a (Fig. 2c in the manuscript). To verify our strategy within the feasible twist-angle range, we selected a sample with twist angle of 26° in the manuscript. Additional samples with twist angle of 33° and -26° were fabricated and measured, as shown in Fig. R4b and d. The experimental results are marked with yellow dots in Fig. R4a. The frequency-dependent rotation feature of the experimental results corresponds well with the prediction in Fig. R4a. For the cases of 33° and 26°, when the excitation frequency increases from 600 cm^{-1} to 630 cm^{-1}, the quasi-1D polariton rotates anticlockwise. When the excitation frequency increases from 670 cm^{-1} to 680 cm^{-1}, the quasi-1D polariton rotates clockwise. The frequency-dependent rotation of the case of -26° is opposite to the former two cases. The experimental results show the robustness of twist angle.

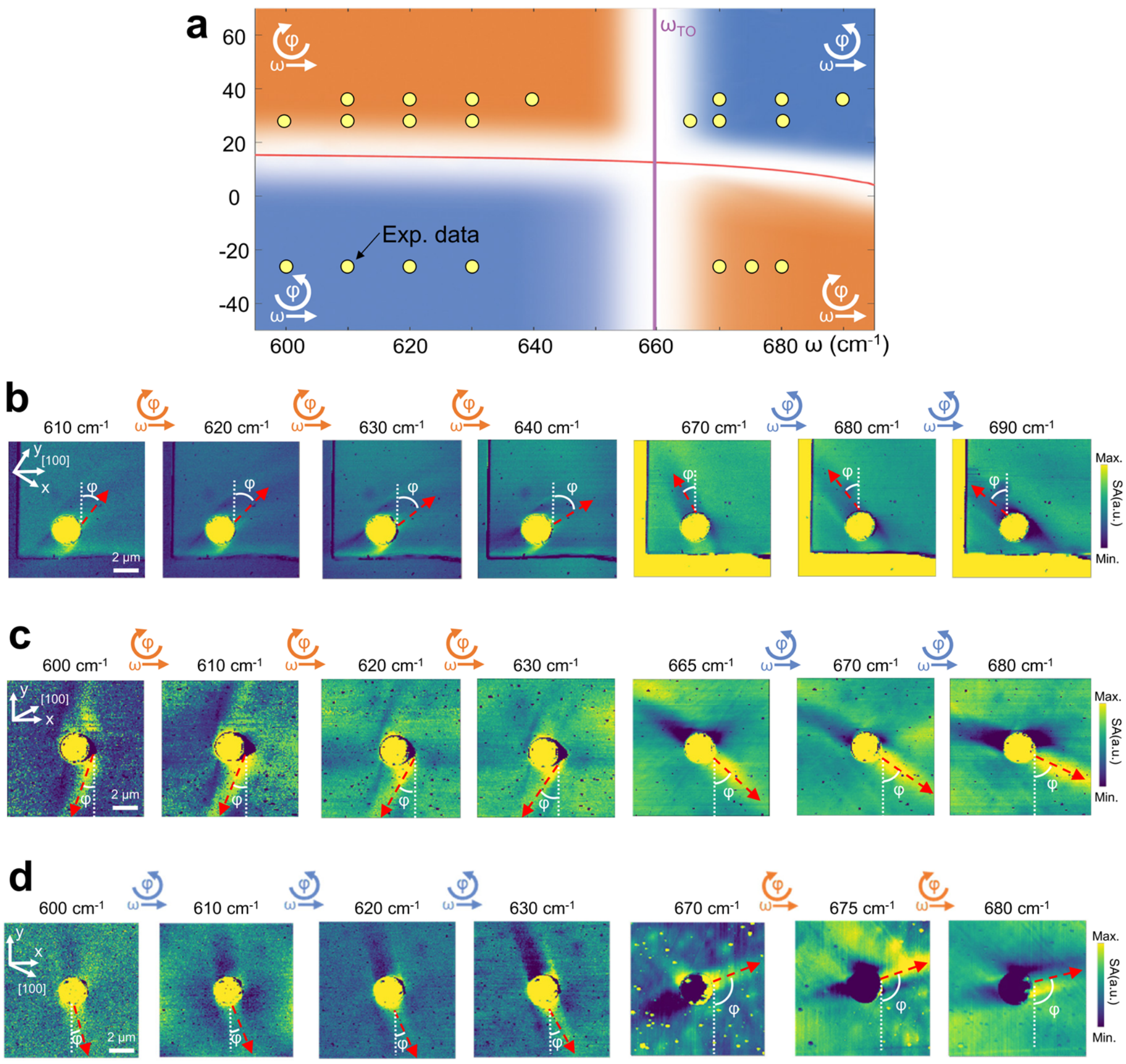

Fig. R4 a, Mapping diagram of frequency-dependent rotation varying with twist angle and frequency. The vertical axis of this plot gives the range of twist angle that supports quasi-1D polariton in α-MoO_3/β-Ga_2O_3 heterostructure. The experimental result points are marked in the diagram. **b,** Measured results for the case of 33°, corresponding to the first row of yellow dots in panel **a**. The gold disk is near the boundary. For excitation frequency higher than the phonon frequency (659 cm^{-1}), the β-Ga_2O_3 substrate has high reflection because of the negative sign of ε_{zz}. **c, d,** Cases of 26° and -26°, corresponding to the second and third row of yellow dots in panel **a**, respectively. The thickness of α-MoO_3 flake is 85 nm in **b**, 100 nm in **c** and 80 nm in **d**.

Changes made in the revised manuscript and supplementary information:

We thank the reviewer for this question, in response to which we have made the following changes in lines 153-158 on Page 5 and 6 of the revised manuscript, as marked in blue:

Text before revision:

"As an example, we fabricated and measured a heterostructure sample with a twist angle of 26°, which is shown in Fig. 2a".

Text after revision:

" The in-plane hyperbolic mode's opening angle of β-Ga_2O_3 offers a broad angular range for leaky channels, enabling quasi-1D polariton to form across a wide range of twist angles. As a representative experimental realization within this regime without losing generality, we fabricated and measured a heterostructure sample with a twist angle of 26° (Fig. 2a). Additional samples with other twist angles yield consistent results (Fig. S12)."

We have also added Fig. R4 as Fig. S12 in the revised supplementary information, as marked in blue:

"Fig. S12: Experimental results of α-MoO_3/β-Ga_2O_3 heterostructure with other twist angles

a, Mapping diagram of frequency-dependent rotation varying with twist angle and frequency. The vertical axis of this plot gives the range of twist angle that supports quasi-1D polariton in α-MoO_3/β-Ga_2O_3 heterostructure. The experimental result points are marked in the diagram. **b,** Measured results for the case of 33°, corresponding to the first row of yellow dots in panel **a**. The gold disk is near the boundary. For excitation frequency higher than the phonon frequency (659 cm^{-1}), the β-Ga_2O_3 substrate has high reflection because of the negative sign of ε_{zz}. **c, d,** Cases of 26° and -26°, corresponding to the second and third row of yellow dots in panel **a**, respectively. The thickness of α-MoO_3 flake is 85 nm in **b**, 100 nm in **c** and 80 nm in **d**."

8. Figure 1a: The crystal structures shown are not clearly identified. If they are not essential to the main message, consider removing them. Additionally, the label "quasi-1D polariton" appears; adding a corresponding "hyperbolic polariton" label may help illustrate the transition from hyperbolic to quasi-1D behavior.

Reply: We thank the reviewer for this suggestion. In the revised manuscript, we have removed the crystal structures. We have also added the "hyperbolic polariton" label to explicitly illustrate the transition to quasi-1D behavior. These updates are reflected in

the revised Figure 1a (Fig. R5).

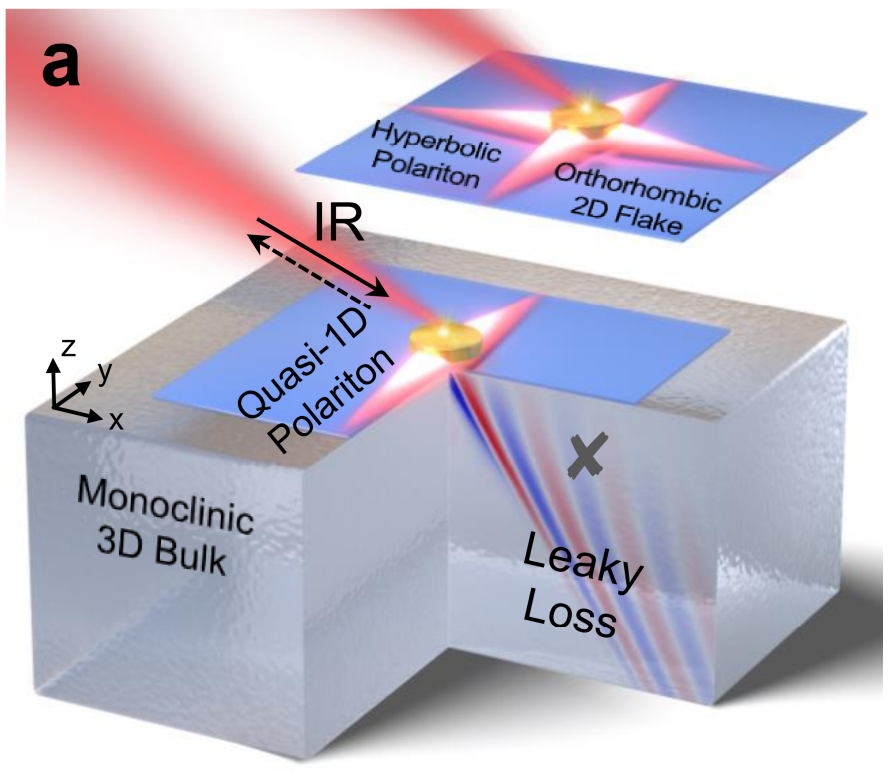

Fig. R5 The revised Figure 1a in our revised manuscript.

Changes made in the revised manuscript and supplementary information:

We thank the reviewer for this question, in response to which we have replaced Fig. 1a with Fig. R5 in the revised manuscript.

9. Figure 1b: The gray "×" marks are not explained. Please add a description in the caption.

Reply: We thank the reviewer for this suggestion. The light gray "×" marks indicate that a part of the hyperbola has been suppressed due to leaky loss into the substrate (corresponding to the dark gray "×" marks in **a**). We have changed the caption accordingly.

Changes made in the revised manuscript and supplementary information:

We thank the reviewer for this question, in response to which we have made the following changes in lines 473-475 on Page 16 of the revised manuscript, as marked in blue:

"The light gray "×" marks indicate that a part of the hyperbola has been suppressed due to leaky loss into the substrate (corresponding to the dark gray "×" marks in **a**)."

10. Figures 3b and 3c: The annotations for k and S in momentum space are clearly indicated. If corresponding real-space images with Fourier transforms can be obtained,

please add annotations for consistency.

Reply: We thank the reviewer for this valuable suggestion. We have already marked the corresponding k and S in corresponding k-space analysis, schematic diagram, real-space images and simulation images in the updated Figure 3 for consistency (Ref. *Sci. Adv.* 2022, 8, eabp8486).

It is worth noting that we redesigned the experimental setup by implementing a gold reflective structure to further demonstrate the ultra-broad-angle zero-reflection phenomenon. Specifically, a single-crystalline gold flake was positioned adjacent to the gold disk to serve as a mirror on the α-MoO_3/β-Ga_2O_3 heterostructure, providing a more robust and well-defined reflective boundary compared to a physical step edge.

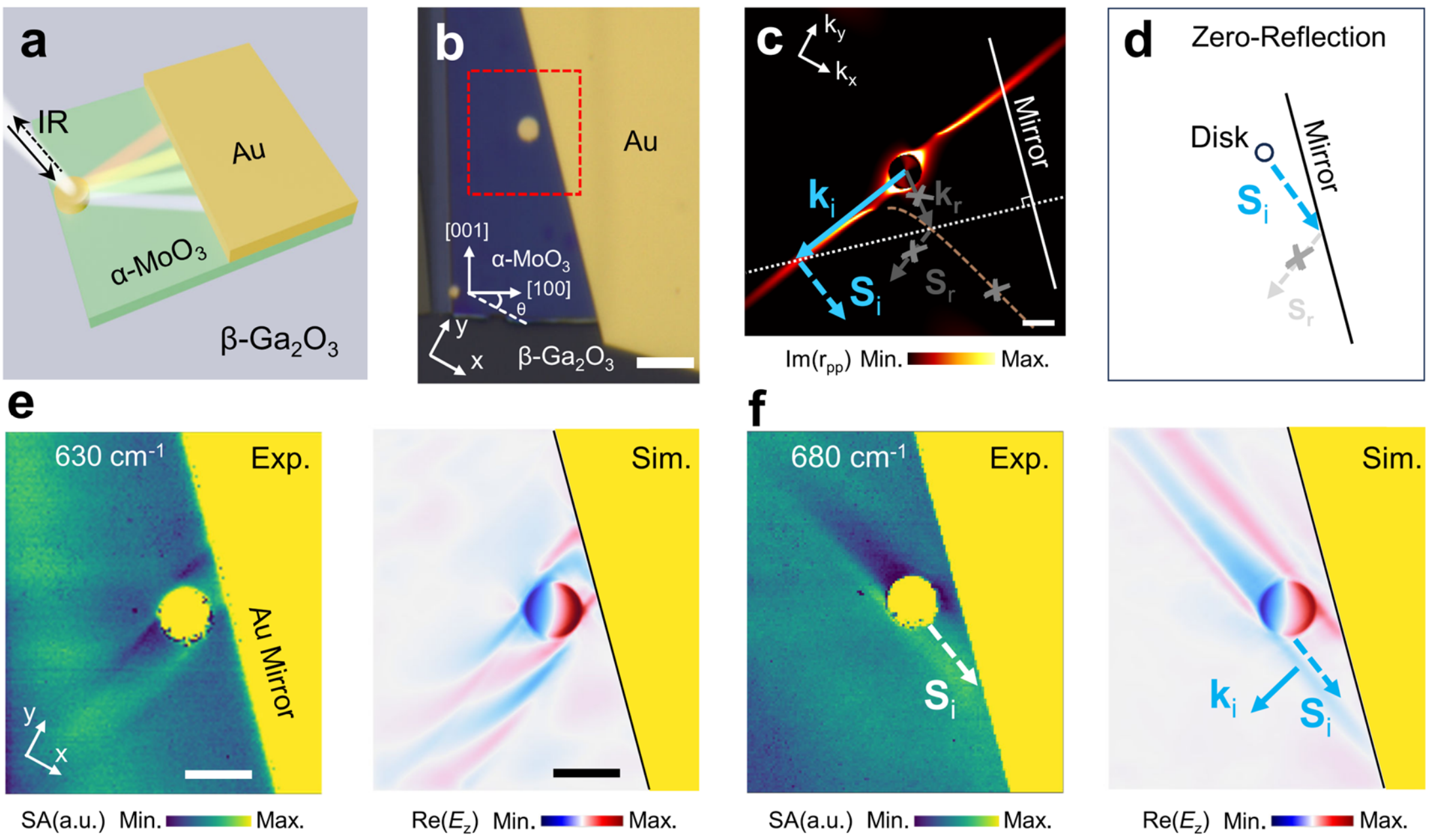

Fig. R6 The revised Fig. 3 in our revised manuscript. Zero reflection of quasi-1D polaritons. **a,** Schematic of wide-band zero-reflection of quasi-1D polariton. Quasi-1D polaritons of different frequencies (indicated by different colors) excited by input light through a gold disk propagates to the gold mirror without reflection. **b,** Microscopic image of the measured α-MoO_3 flake/β-Ga_2O_3 bulk heterostructure with a twist angle θ of 26°, featuring a central gold disc as the excitation source. A single crystal of gold was placed beside the gold disc as a mirror. The thickness of the thinner α-MoO_3 is about 120 nm. The twist angle is 26°. The scale bar in **b** represents 5 µm. **c,** The theoretical analysis in momentum space of zero reflection of quasi-1D polariton at the mirror. The blue solid and dashed arrows show the incident wave vector and corresponding Poynting vector. The gray

solid and dashed vectors show the reflective wave vector and Poynting vector that would occur if it were a hyperbolic situation (brown dashed curve), which are eliminated by the leakage mechanism. Subscripts "i" and "r" stand for incidence and reflection. The scale bar in **c** represents $2k_0$. **d**, The simplified real space schematic of zero-reflection transmission corresponding to the case in **c**. **e-f**, The experimental (left) and simulation (right) results of zero-reflection of quasi-1D polaritons of the heterostructure (measured in the red dashed box in b) for excitation frequency of 630 cm^{-1} (**e**) and 680 cm^{-1} (**f**). The quasi-1D polaritons propagate towards the boundary with no reflection, which is verified by the simulation results. The scale bar in **e** represents 2 μm.

Changes made in the revised manuscript and supplementary information:

We thank the reviewer for this question, in response to which we have updated Fig. 3 with Fig. R6 in the revised manuscript and made the following changes in the revised manuscript, as marked in blue:

In lines 225-229 on Page 8:

"The wideband zero reflection is verified in experiment with s-SNOM using the sample in Fig. 3b. The experimental results are shown in the left panels of Fig. 3e and f. Cases of 630 and 680 cm^{-1} are presented for demonstration. Simulation results are performed using the measured geometry parameters and correspond well with the experiment results, shown in the right panels of Fig. 3e and f."

In lines 501-515 on Page 18:

Text before revision:

"**a**, Schematic of wide-band zero-reflection of quasi-1D polariton. Two bricks with different colors represent different thickness. Quasi-1D polaritons with different frequencies excited by input light through a gold disk propagates cross the step without reflection. **b**, The theoretical analysis in momentum space of zero reflection of quasi-1D polariton at the boundary. The blue solid and dashed arrows show the incident wave vector and corresponding Poynting vector. The gray solid and dashed vectors show the reflective wave vector and Poynting vector, which are forbidden by the IFC. **c**, The simplified real space schematic of zero-reflection transmission corresponding to the case in **b**. Subscripts "i", "r" and "t" stand for incidence, reflection and transmission. **d**,

The experimental and simulation results of zero-reflection in α-MoO_3/β-Ga_2O_3, with a step height of 60 nm, measured with AFM (white dashed line). The thickness of the thinner α-MoO_3 is about 120 nm. The twist angle is 26°. The presented frequencies are 630 cm^{-1} and 680 cm^{-1}. The quasi-1D polariton crosses the boundary with no reflection, which is verified by the simulation results on the right side."

Text after revision:

" **a,** Schematic of wide-band zero-reflection of quasi-1D polaritons whose frequencies are indicated by different colors. **b,** Microscopic image of the measured sample. The twist angle is 26°. A single crystal gold plate is used as a mirror. The thickness of the α-MoO_3 is 120 nm. The scale bar is 5 μm. **c**, The theoretical analysis of zero reflection. The blue solid and dashed arrows show the incident wave vector and corresponding Poynting vector. The gray solid and dashed vectors show the reflective wave vector and Poynting vector that would occur if it was a hyperbolic situation (brown dashed curve), which are eliminated by the leakage mechanism. Subscripts "i" and "r" stand for incidence and reflection. The scale bar is $2k_0$. **d**, Simplified real space schematic of zero-reflection of the case in **c**. **e-f**, The experimental (left) and simulation (right) results of zero-reflection of quasi-1D polaritons of the heterostructure (measured in the red dashed box in b) for excitation frequency of 630 cm^{-1} (**e**) and 680 cm^{-1} (**f**). The quasi-1D polaritons propagate towards the boundary with no reflection, which is verified by the simulation results. The scale bar is 2 μm."

11. Figure 4a: Different atoms (e.g., W and Mo) should preferably be distinguished using distinct colors. The labeled arrows a, b, and c should also be explained in the caption.

Reply: We thank the reviewer for this helpful suggestion. In the revised Figure 4a, we have used distinct colors to differentiate different atoms. We have also updated the figure caption to specify that the arrows labeled a, b, and c indicate the directions of the three basis vectors of the crystal lattice. The modified figure is shown in Fig. R7.

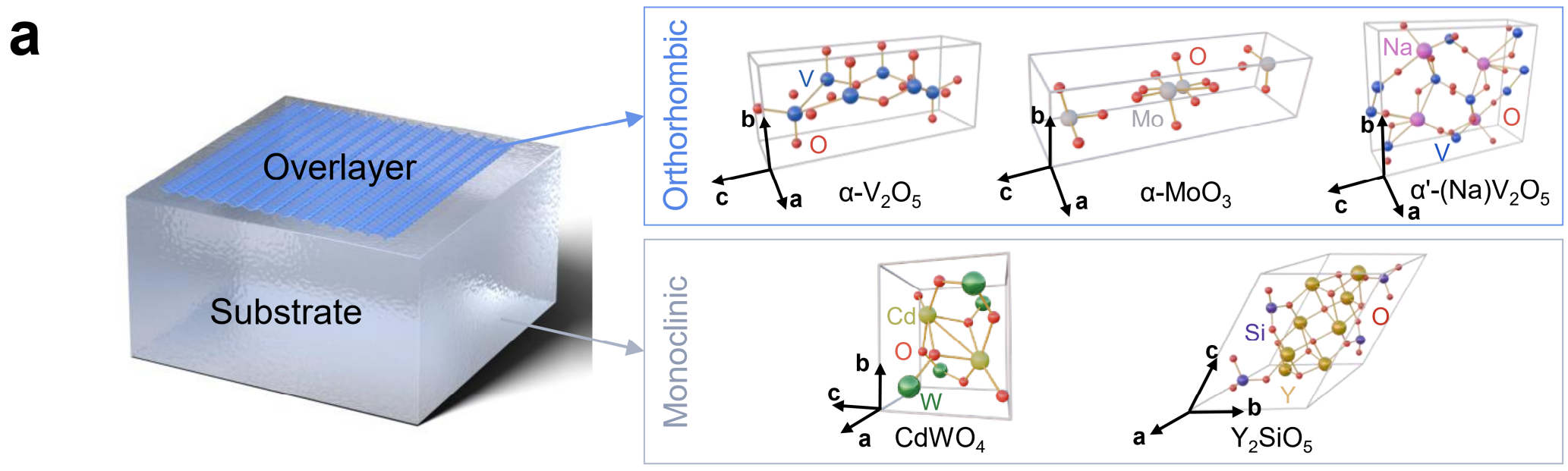

Fig. R7 The revised Fig. 4a in our revised manuscript.

Changes made in the revised manuscript and supplementary information:

We thank the reviewer for this question, in response to which we have replaced Fig. 4a with Fig. R7 in the revised manuscript and made the following changes in lines 524-526 on Page 19 of the revised manuscript, as marked in blue:

"Different kinds of atoms are marked with different colors. The arrows labeled a, b, and c indicate the directions of the three basis vectors of the crystal lattice."

12. Figure 4c and broadband coverage: The text claims $\Delta\lambda \sim 7$ μm, but Figure 4c shows two separated bands (10.4–12.3 μm and 13.3–18.2 μm) with a gap. The authors should clarify whether "broadband" refers to the total coverage range or a single continuous band.

Reply: Thank you for the reviewer's suggestion. The $\Delta\lambda \sim 7$ μm reported in Figure 4c refers to the overall spectral range covered by several representative material combinations, rather than a continuous band for a single heterostructure. The two separate bands shown in the figure (10.4–12.3 μm and 13.3–18.2 μm) correspond to different material combinations, respectively. We have clarified the definition of "broadband" in the revised manuscript to avoid any misunderstanding. Furthermore, the generality of this strategy means that by choosing different combinations of 2D vdW films and 3D bulk substrates, quasi-1D polaritons can be realized across an even broader range of frequencies. Therefore, the spectral range shown in the figure is provided as an example and does not represent the upper limit achievable with this approach.

Changes made in the revised manuscript and supplementary information:

We thank the reviewer for this question, in response to which we have made the following changes in lines 274-276 on Page 10 of the revised manuscript:

Text before revision:

"their bandwidth can cover Δλ~"

Text after revision:

"the overall spectral range covered by several representative material combinations exceeds 7 μm"

Reviewer #2

Comment: The manuscript by Hongyi Yuan et al. proposes a loss-engineering strategy that harnesses optical leakage loss as a degree of freedom for modulation, aiming to achieve reconfigurable control of quasi-1D polaritons and ultra-broad-angle reflectionless transmission in 2D/3D heterostructures. The approach is interesting.

Reply: We thank the reviewer for the positive assessment and constructive suggestions, which have helped us further improve the revised manuscript. Below we address the reviewer's comments point by point.

However, the experimental data currently presented in the manuscript seems do not sufficiently substantiate the claimed ultra-broad-angle reflectionless transmission and the universality of the heterostructure platform. Furthermore, the interpretation of the leakage mode mechanism lacks sufficient detail. Therefore, I cannot recommend publication of this manuscript in its present form, although the results may become suitable for Nature Communications after the following issues are clarified and improved.

Reply: Thank you for the reviewer’s suggestions. In response to the two points raised, we have made the following changes in the revised manuscript:

(1) Further experiments demonstrating ultra-broad-angle reflectionless transmission:

To further demonstrate the ultra-broad-angle zero-reflection phenomenon, we

redesigned the experimental setup by implementing a gold reflective structure. Specifically, a single-crystalline gold flake was positioned adjacent to the gold disk to serve as a mirror on the α-MoO_3/β-Ga_2O_3 heterostructure, providing a more robust and well-defined reflective boundary compared to a physical step edge (updated in Figure 3 of the revised main text). Experimentally, we observed unambiguous zero reflection of quasi-1D polaritons (Fig. R8b, c). As contrast, by tuning the operating frequency to 755 cm^{-1}, where the permittivities along the three principal axes of β-Ga_2O_3 are all positive (i.e., behaving as an ordinary dielectric), clear reflection of hyperbolic polaritons of α-MoO_3 was observed (Fig. R8d). The zero-reflection phenomenon of quasi-1D polariton is not limited to gold mirrors, it can also be observed in boundary of α-MoO_3/air at the boundary of the α-MoO_3 flake (Fig. R9). These results of this experiment are in excellent agreement with our theoretical predictions, thereby validating the robustness of the ultra-broad-angle zero-reflection effect.

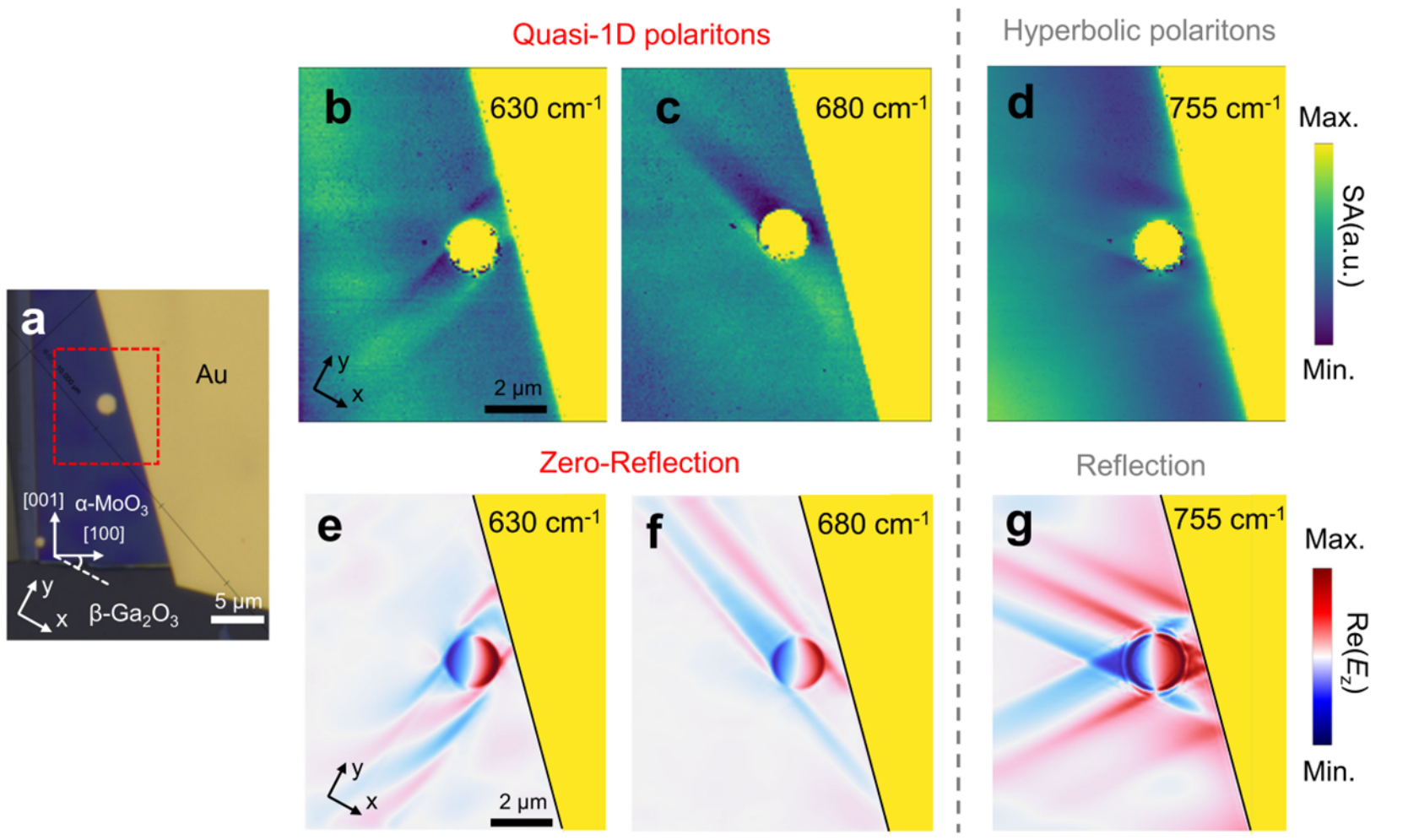

Fig. R8 The near-field images of quasi-1D polaritons with gold mirror. a, Microscopic image of the measured α-MoO_3 flake/β-Ga_2O_3 bulk heterostructure with a twist angle θ of 26°, featuring a central gold disc as the excitation source. A single crystal of gold was placed beside the gold disc as a mirror. The thickness of the thinner α-MoO_3 is about 120 nm. The twist angle is 26°. **b-d**, The experimental results of ultra-broad-angle zero-reflection of quasi-1D polaritons **(b,c)** and reflection of hyperbolic polaritons of the heterostructure (measured in the red dashed box in **a**). **e-g**, Simulation corresponding to **b-d**.

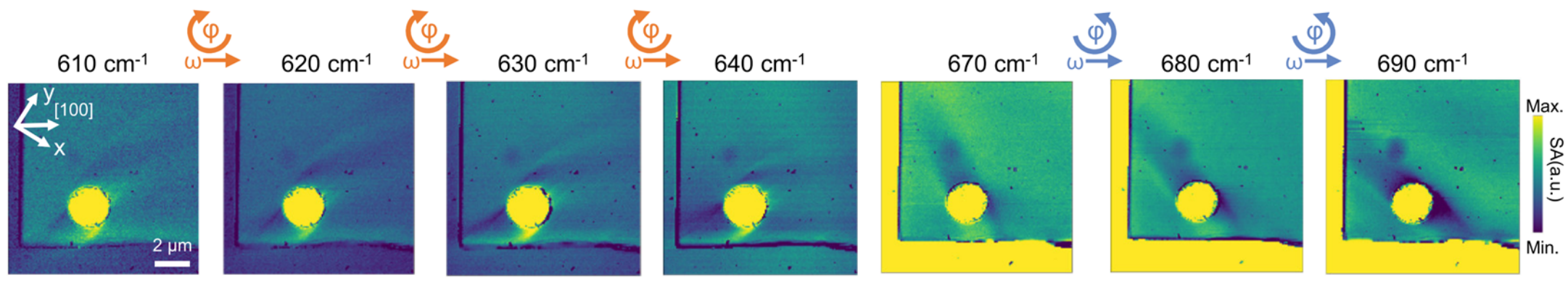

Fig. R9 The near-field images of quasi-1D polaritons with boundary of α-MoO_3/air. The twist angle of the α-MoO_3/β-Ga_2O_3 heterostructure is 33°. A gold disk is placed near the boundary. The thickness of α-MoO_3 flake is 85 nm.

(2) Supplement on the generality of the heterostructure platform:

We have extended the experimental platform from α-MoO_3/β-Ga_2O_3 to an α-MoO_3/$CdWO_4$ heterostructure. On this new platform, we not only observed quasi-one-dimensional polaritons but also demonstrated ultra-wide-angle zero reflection over a broad frequency range (Fig. R10, updated in Figure 4c-e of the revised main text), providing further validation of the generality of our strategy.

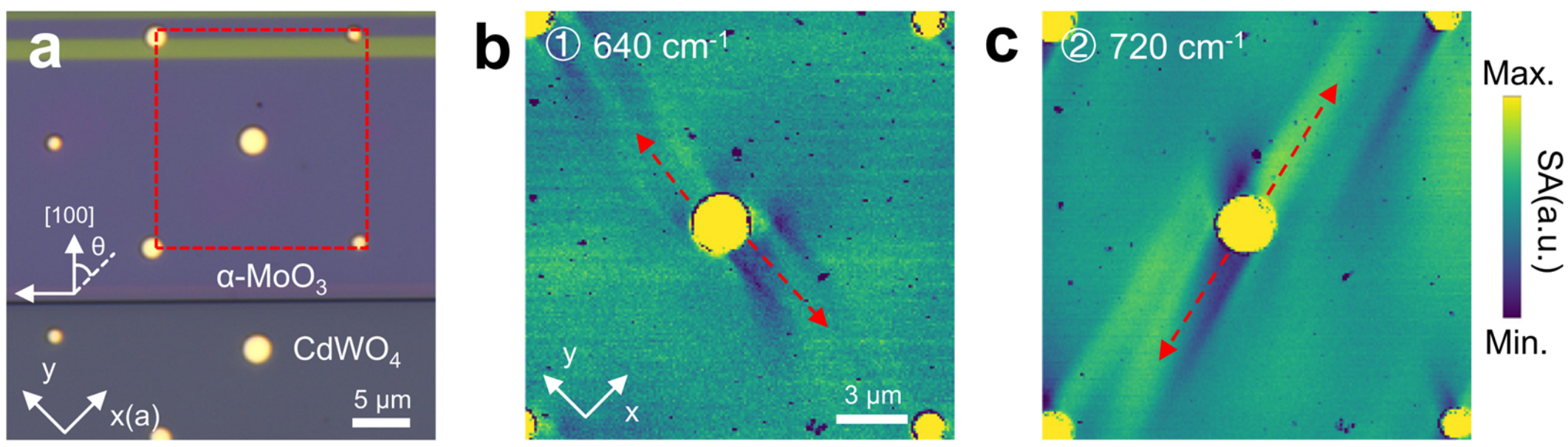

Fig. R10 The near-field images of quasi-1D polaritons in α-MoO_3/$CdWO_4$ heterostructure. a, Microscopic image of the measured 200-nm-thick α-MoO_3 flake/$CdWO_4$ bulk heterostructure, featuring a central gold disk as the excitation source. The twist angle between the [100] axis of these two kinds of material is 45°. **b, c,** Near-field optical amplitude distribution illustrating the quasi-1D polariton of the heterostructure

(3) Elucidation of the leakage mode mechanism:

The optical loss of phonon polaritons propagating in high-quality thin films can generally be classified into intrinsic material loss (i.e., phonon absorption) and extrinsic loss (Ref. *Nat. Commun.*, 2020, 11, 3649). Extrinsic loss can be further divided into two

components: scattering loss arising from radiation of polariton electromagnetic energy into the air, and leakage loss resulting from radiative coupling of electromagnetic energy into the substrate. Notably, phonon polaritons possess large in-plane wavevectors due to strong spatial confinement, which creates a significant momentum mismatch with external media (such as air or dielectric substrates) and strongly suppresses radiative coupling. Building upon this, in our work we introduce a phonon-induced, switchable leakage loss mechanism as a tunable loss-engineering platform: the substrate's hyperbolic bulk mode extends the continuous wavevector range to match the large in-plane wavevector, thereby enabling angle-selective radiative leakage of phonon polaritons from the top film into the substrate. This approach allows for the on-demand conversion of mirror-symmetric hyperbolic polaritons into quasi-1D propagating states.

Changes made in the revised manuscript and supplementary information:

We thank the reviewer for this question, in response to which we have made the following changes in the revised manuscript, as marked in blue:

In lines 201-205 in Page 7:

"A single-crystal gold flake is placed on top of the heterostructure. Due to its high reflectivity, the edge of the gold flake serves as a mirror to facilitate the observation of zero-reflection phenomena. A gold disk is placed on top of the heterostructure to excite quasi-1D polaritons which propagates towards the gold edge. The microscope image of the fabricated sample is shown in Fig. 3b. "

In lines 225-235 in Page 8:

" The wideband zero reflection is verified in experiment with s-SNOM using the sample in Fig. 3b. The experimental results are shown in the left panels of Fig. 3e and f. Cases of 630 and 680 cm^{-1} are presented for demonstration. Simulation results are performed using the measured geometry parameters and correspond well with the experiment results, shown in the right panels of Fig. 3e and f. No reflection is observed in the measured near field signal and simulation, and it should be noted that when quasi-1D polaritons arrive at the gold edge, its energy flow transmits into the α-MoO_3 layer beneath the gold flake, which cannot be observed in the measured near-field image. For

comparison, the reflection of hyperbolic polariton at the gold mirror is given in Fig. S22. The zero-reflection phenomenon of quasi-1D polariton is not limited to gold-mirror interfaces but is also observed at the natural boundaries of α-MoO_3 (Fig. S12b)."

In lines 277-282 in Page 10:

" To verify the generality of our scheme, we used the α-MoO_3/$CdWO_4$ heterostructure as an example to verify the reconfigurable propagation characteristics of quasi-1D polaritons. The microscope image is shown in Fig. 4c. By designing an appropriate twist angle for the heterostructure, a clear ray-like propagation of quasi-1D polaritons is achieved, with frequencies located at 640 cm^{-1} (Fig. 4d), and 720 cm^{-1} (Fig. 4e), respectively."

In lines 530-534 in Page 19:

"**c**, Microscopic image of the measured 200-nm-thick α-MoO_3 flake/$CdWO_4$ bulk heterostructure, featuring a central gold disk as the excitation source. The twist angle θ between the [100] axis of these two kinds of material is 45°. **d, e,** Near-field optical amplitude distribution illustrating the quasi-1D polariton of the heterostructure, corresponding to the cases ①, ② marked in **b**."

We have also added Fig. R8 as Fig. S22 and Fig. R9 as Fig. S12b of the revised supplementary information, as marked in blue:

"Fig. S22: The near-field images of quasi-1D polaritons with gold mirror.

a, Microscopic image of the measured α-MoO_3 flake/β-Ga_2O_3 bulk heterostructure with a twist angle θ of 26°, featuring a central gold disc as the excitation source. A single crystal of gold was placed beside the gold disc as a mirror. The thickness of the thinner α-MoO_3 is about 120 nm. The twist angle is 26°. **b-d**, The experimental results of ultra-broad-angle zero-reflection of quasi-1D polaritons **(b,c)** and reflection of hyperbolic polaritons of the heterostructure (measured in the red dashed box in **a**). **e-g**, Simulation corresponding to **b-d**. By tuning the operating frequency to 755 cm^{-1}, where the permittivities along the three principal axes of β-Ga_2O_3 are all positive (i.e., behaving as an ordinary dielectric), clear reflection of hyperbolic polaritons of α-MoO_3 was observed (**d, g**)"

"Fig. S12: Experimental results of α-MoO_3/β-Ga_2O_3 heterostructure with other

twist angles

a, Mapping diagram of frequency-dependent rotation varying with twist angle and frequency. The vertical axis of this plot gives the range of twist angle that supports quasi-1D polariton in α-MoO_3/β-Ga_2O_3 heterostructure. The experimental result points are marked in the diagram. **b,** Measured results for the case of 33°, corresponding to the first row of yellow dots in panel **a**. The gold disk is near the boundary. For excitation frequency higher than the phonon frequency (659 cm^{-1}), the β-Ga_2O_3 substrate has high reflection because of the negative sign of ε_{zz}. **c, d,** Cases of 26° and -26°, corresponding to the second and third row of yellow dots in panel **a**, respectively. The thickness of α-MoO_3 flake is 85 nm in **b**, 100 nm in **c** and 80 nm in **d**."

1. What is the propagation length of quasi-1D polaritons at different frequencies, and how is the loss quantitatively evaluated? Furthermore, what are the respective influences of substrate loss and flake thickness on the propagation length?

Reply: Thanks for the reviewer's valuable question. We conducted the following quantitative analysis to illustrate the propagation characteristics of quasi-1D polaritons. (1) To evaluate the propagation loss quantitatively, we extracted the propagation data from the simulations generated by a point dipole of different frequencies and performed the fitting of the attenuation coefficients α according to an exponential function $e^{-\alpha r}$ (Fig. R11), where r represents the distance from the excitation source (Ref. *Nat. Commun.*, 2024, 15, 9042). The calculated propagation length (i.e., 1/α) corresponding to the electric field amplitude decay to 1/*e* ranges from 2.3 to 2.7 μm. Experimentally, we extracted the propagation data from the experiments generated by a gold disk and performed the fitting of the attenuation coefficients α, and the calculated propagation length (i.e., 1/α) is 3.8 μm. Additionally, near-field experimental images reveal that the quasi-1D polaritons form ray-like (non-diffracting) patterns extending over a distance of up to 11 μm in Fig. R12 (following the definition of polariton characteristic length in Ref. *Nature* 2021, 596, 362).

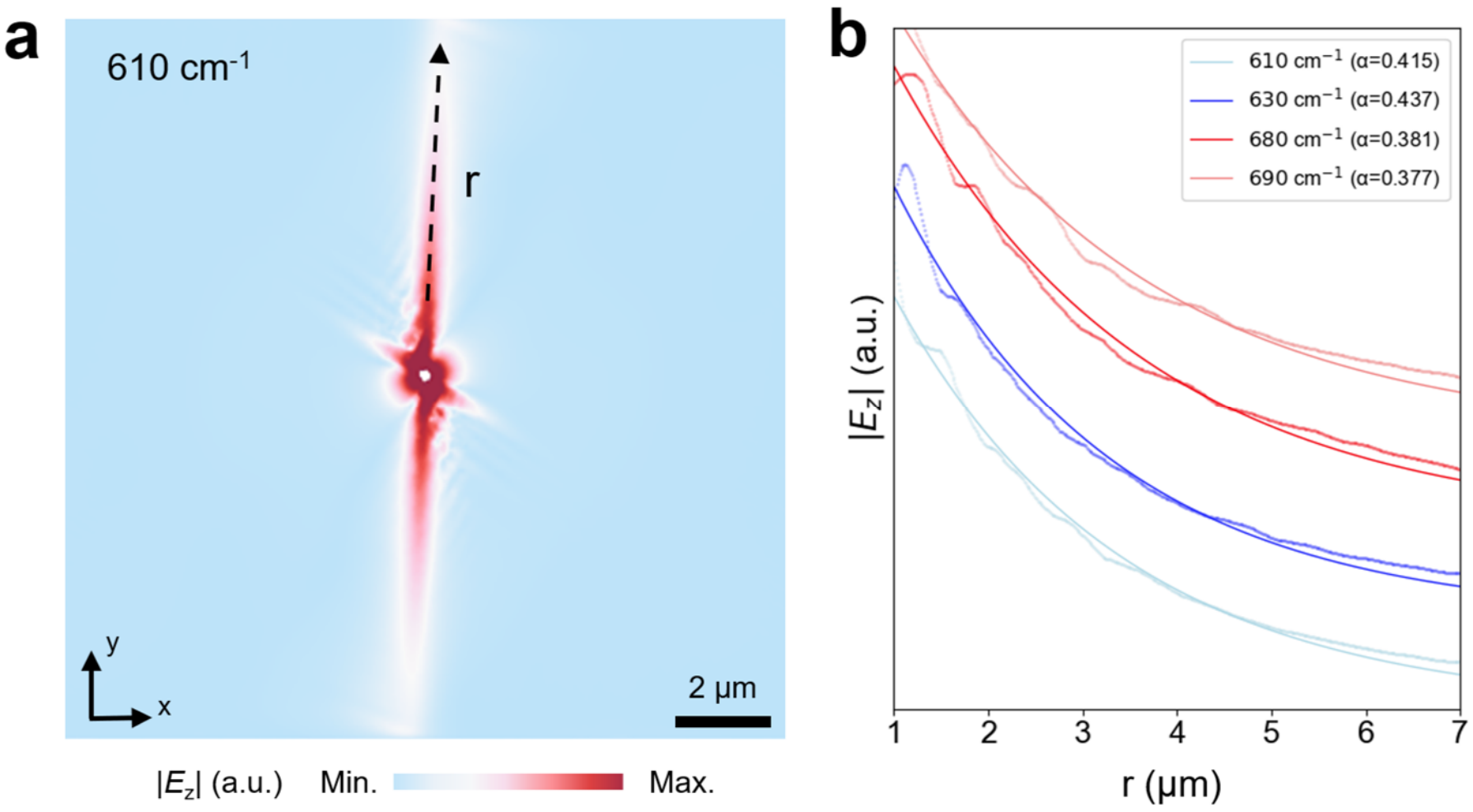

Fig. R11 Propagation analysis of real space distribution of quasi-1D polaritons generated by a point dipole. a, Electric field component $|\mathbf{E}_z|$ of 610 cm^{-1} generated by a point dipole of a 400 nm-thick α-MoO_3 film / bulk β-Ga_2O_3 heterostructure from simulations. The twist angle is 26°. **b**, Simulation profiles (scattered points) along the wavenumber-dependent propagation directions shown for distances of 1–7 μm from the dipole source (dashed line in panel **a** illustrates one example), with colors denoting cases of distinct wavenumbers. Different colored solid lines represent the curves fitted according to an exponential function $e^{-\alpha r}$. The attenuation coefficients α of different frequencies are displayed in the legend, and r represents the distance from the excitation source.

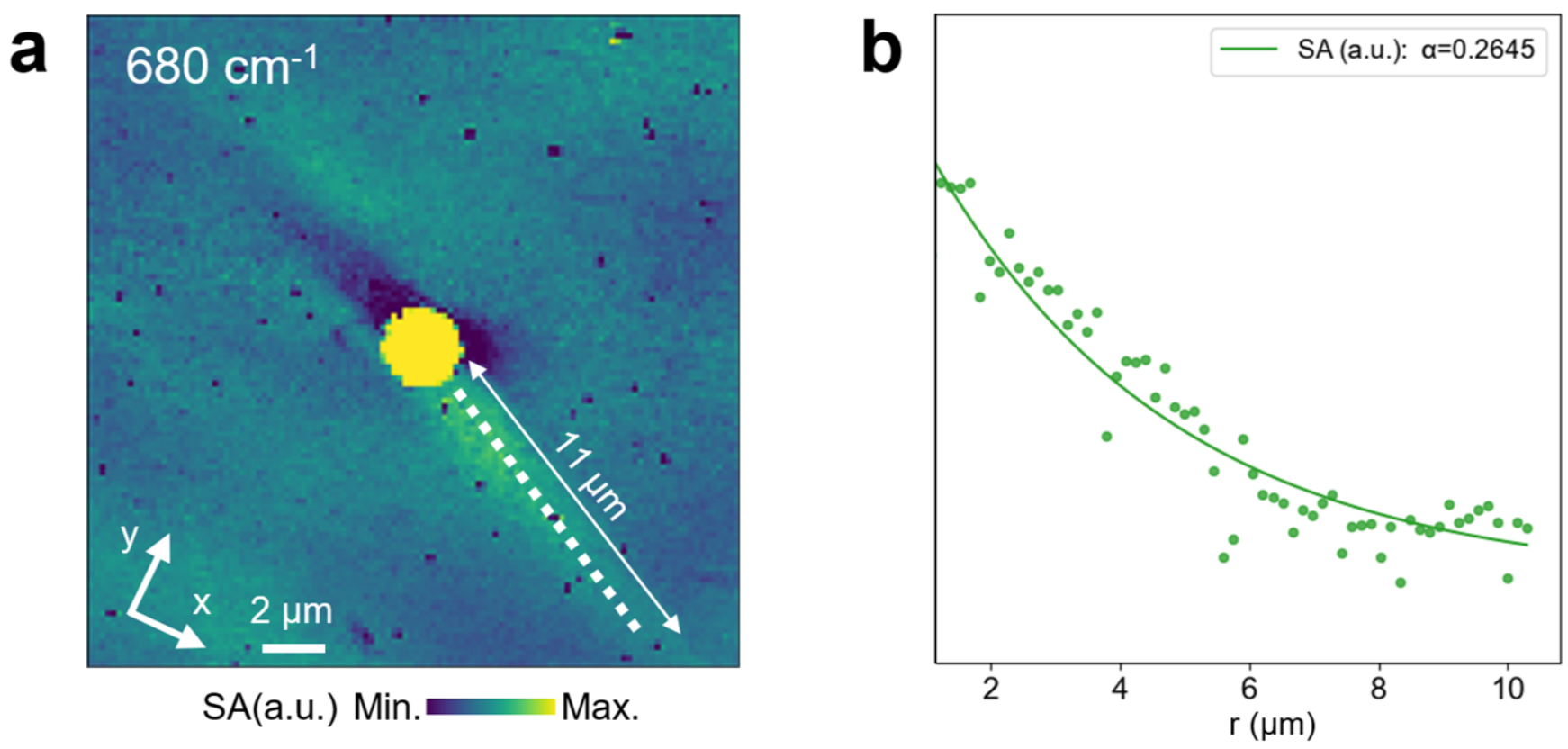

Fig. R12 Propagation analysis of real space distribution of quasi-1D polaritons generated by a gold disk. a, near-field images of 680 cm^{-1} generated by a gold disk of a 100 nm-thick α-MoO_3 film / bulk β-Ga_2O_3 heterostructure. The twist angle is 26°. **b**, experimental profiles (scattered points) along dashed line in panel **a**. Solid line represents the curve fitted according to an exponential

function $e^{-\alpha r}$. The attenuation coefficients α are displayed in the legend, and r represents the distance from the excitation source.

(2) Increasing the substrate's intrinsic loss will reduce the propagation length of quasi-1D polaritons. Within the range of angles where part of hyperbolic polariton is retained to form quasi-1D polaritons, the role of the substrate becomes that of a common dielectric substrate or metalloid substrate (depends on the sign of the real part out-of-plane permittivity). At this point, it is reasonable that increasing the substrate's loss will reduce the propagation length of the polariton mode. As shown in Fig. R13, the propagation length is reduced when increasing the loss of the substrate.

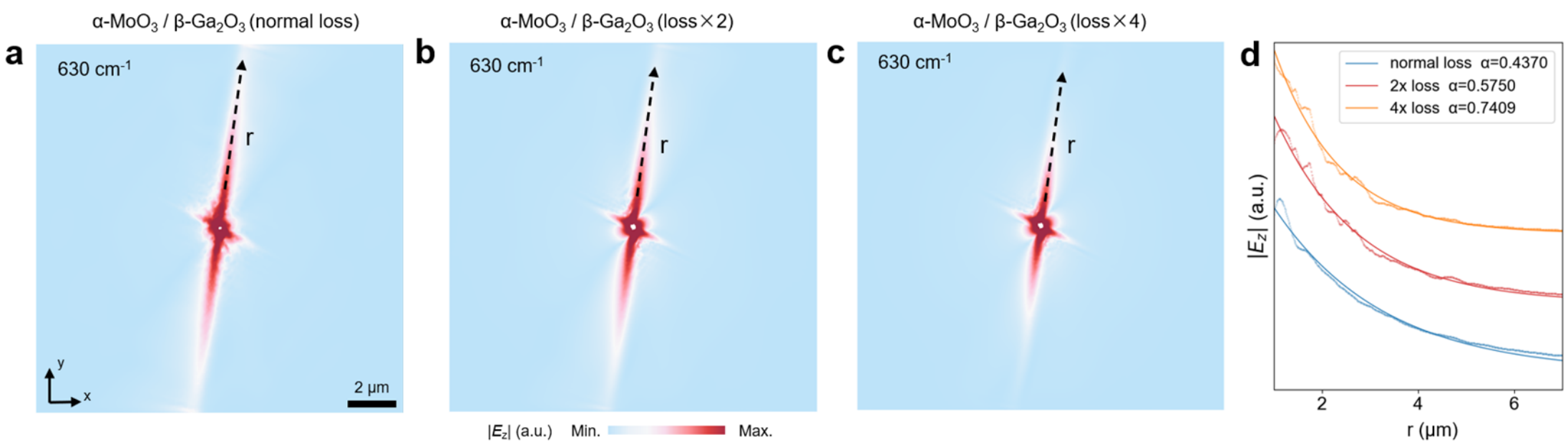

Fig. R13 Propagation analysis of real space distribution of quasi-1D polaritons with high-loss substrate. a, Electric field component $|\mathbf{E}_z|$ of 630 cm^{-1} generated by a point dipole of a 400 nm-thick α-MoO_3 film / bulk β-Ga_2O_3 heterostructure from simulations. The twist angle is 26°. **b, c,** Simulation of multiplying the imaginary parts of the dielectric functions in all directions of the substrate by 2 (**b**) or 4 (**c**), while maintaining the other conditions consistent with those in panel **a**. **d,** Simulation profiles (scattered points) along the wavenumber-dependent propagation directions shown for distances of 1–7 μm from the dipole source (dashed lines in panel **a-c**), with colors denoting cases of distinct times of loss. Different colored solid lines represent the curves fitted according to an exponential function $e^{-\alpha r}$. The attenuation coefficients α of different frequencies are displayed in the legend.

(3) To illustrate the influence of flake thickness on the propagation length, we have simulated the propagation of quasi-1D polariton excited by a gold disk, with the flake

thickness varied from 50 to 400 nm (Fig. R14). The frequency is 680 cm^{-1}. Overall, the propagation length of quasi-1D polaritons is not affected obviously by the upper flake thickness.

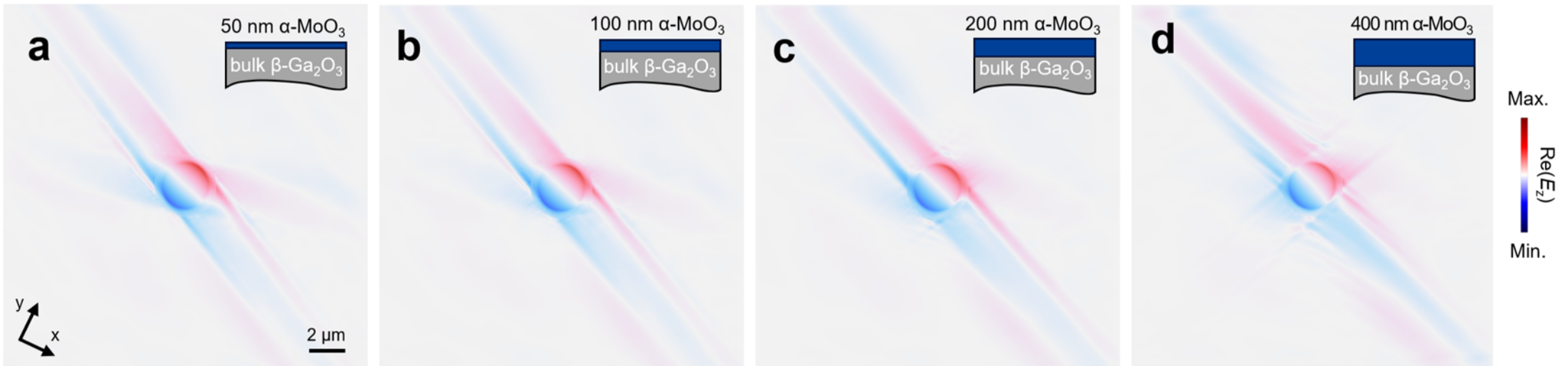

Fig. R14 The real part of z-component of electric field distribution of the heterostructure with the α-MoO_3 flake thickness of 50 (**a**), 100 (**b**), 200 (**c**), and 400 (**d**) nm.

Changes made in the revised manuscript and supplementary information:

We thank the reviewer for this question, in response to which we have made the following changes in the revised manuscript:

In lines 181-183 on Page 6:

"The analysis of quasi-1D polariton ray-like (diffraction-less) patterns[10], which extend over a distance up to 11 μm, is presented in Fig. S15 and Fig. S16."

In lines 193-195 on Page 7:

"Further studies demonstrate that the propagation characteristics of these quasi-1D polaritons are robust against variations in both the incident direction of the excitation light (Supplementary Note 12 and Fig. S19) and the α-MoO_3 flake thickness (Fig. S20)."

We have also included Fig. R11 as Fig. S15, Fig. R12 as Fig. S16, and Fig. R14 as Fig. S20 in the revised supplementary information, as marked in blue:

" **Fig. S15: Propagation analysis of quasi-1D polaritons generated by a point dipole.** **a**, Electric field component |$\mathbf{E}_z$| of 610 cm^{-1} generated by a point dipole of a 400 nm-thick α-MoO_3 film / bulk β-Ga_2O_3 heterostructure from simulations. The twist angle is 26°. **b**, Simulation profiles (scattered points) along the wavenumber-dependent propagation directions shown for distances of 1–7 μm from the dipole source (dashed line in panel **a** illustrates one example), with colors denoting cases of distinct wavenumbers. Different colored solid lines represent the curves fitted according to an

exponential function $e^{-\alpha r}$. The attenuation coefficients α of different frequencies are displayed in the legend, and r represents the distance from the excitation source [13]. The calculated propagation length (i.e., 1/α) corresponding to the electric field amplitude decay to 1/*e* ranges from 2.3 to 2.7 μm."

" Fig. S16: Propagation analysis of quasi-1D polaritons generated by a gold disk.

a, near-field images of 680 cm^{-1} generated by a gold disk of a 100 nm-thick α-MoO_3 film / bulk β-Ga_2O_3 heterostructure. The twist angle is 26°. **b**, experimental profiles (scattered points) along dashed line in panel **a**. Solid line represents the curve fitted according to $e^{-\alpha r}$. The attenuation coefficients α are displayed in the legend, and r represents the distance from the excitation source. We calculated propagation length (i.e., 1/α) which is ~3.8 μm. Additionally, near-field experimental image reveals that the quasi-1D polaritons form ray-like (non-diffracting) patterns extending over a distance up to 11 μm, as shown in **a** (following the definition of polariton characteristic length in reference [14])."

"Fig. S20: Simulation of propagation with different flake thickness.

The real part of z-component of electric field distribution of the heterostructure with the α-MoO_3 flake thickness of 50 (**a**), 100 (**b**), 200 (**c**), and 400 (**d**) nm. The frequency is 680 cm^{-1}. Overall, the propagation length of quasi-1D polaritons exhibit robustness with respect to the thickness of the flake varied from 50 to 400 nm."

2. The frequency-dependent rotation of quasi-1D polaritons is only qualitatively shown. A quantitative analysis (e.g., rotation angle vs. frequency) is needed. Is this rotation linear or nonlinear, and does it arise from intrinsic material dispersion or heterostructure coupling? More importantly, does the substrate material itself independently influence the rotation angle beyond serving as a leaky channel?

Reply: Thank you for the suggestion. We have performed a quantitative analysis of the frequency-dependent rotation and provide below a point-by-point explanation of its nonlinear characteristics, physical origin, and the role of the substrate:

(1) Quantitative analysis of the frequency-dependent rotation. We extracted the angular distribution of the electric-field intensity along a circular path of radius 5 μm centered

on the excitation source, quantitatively revealing how the propagation direction of the quasi-1D polariton evolves with frequency (see Fig. R15). As shown in Fig. R15b, the peak position of the angular spectrum exhibits opposite shifting trends above and below 659 cm^{-1} (the curves span blue-to-purple and purple-to-red color gradients, respectively), directly confirming that the rotation direction reverses at this critical frequency.

(2) Nonlinear characteristics of the rotation and their physical origin. The rotation angle of the quasi-1D polariton exhibits a nonlinear dependence on frequency. By measuring the frequency-dependent rotation angle of the quasi-1D polariton with 5 cm^{-1} steps, we find that the angle change is not kept the same. This arises primarily from the intrinsic dispersion of the in-plane dielectric function of the anisotropic film. Specifically, its real part follows a Lorentzian oscillator model with an inherently nonlinear spectral dependence that directly governs the opening angle of the hyperbolic isofrequency contour (Ref. *Sci. Adv.* 2019, 5, eaav8690), consequently giving rise to a nonlinear frequency dependence of the rotation angle. The rotation is therefore driven essentially by the intrinsic material dispersion that shapes the film's hyperbolic topology, rather than by heterostructure coupling.

(3) Role of the substrate. The substrate serves primarily as a tunable leakage channel for on-demand tailoring of the quasi-1D propagation characteristics of in-plane polaritons. The quasi-1D modes retained after leakage screening are inherited predominantly from the intrinsic polariton modes of the 2D film; the dielectric environment of the three-dimensional substrate introduces a moderate angular shift of the quasi-1D isofrequency contour (as illustrated by the comparison between the quasi-1D polariton and the α-MoO_3 M_0 mode, shown in Fig. R16), but its effect on the frequency-dependent rotation trend is negligible.

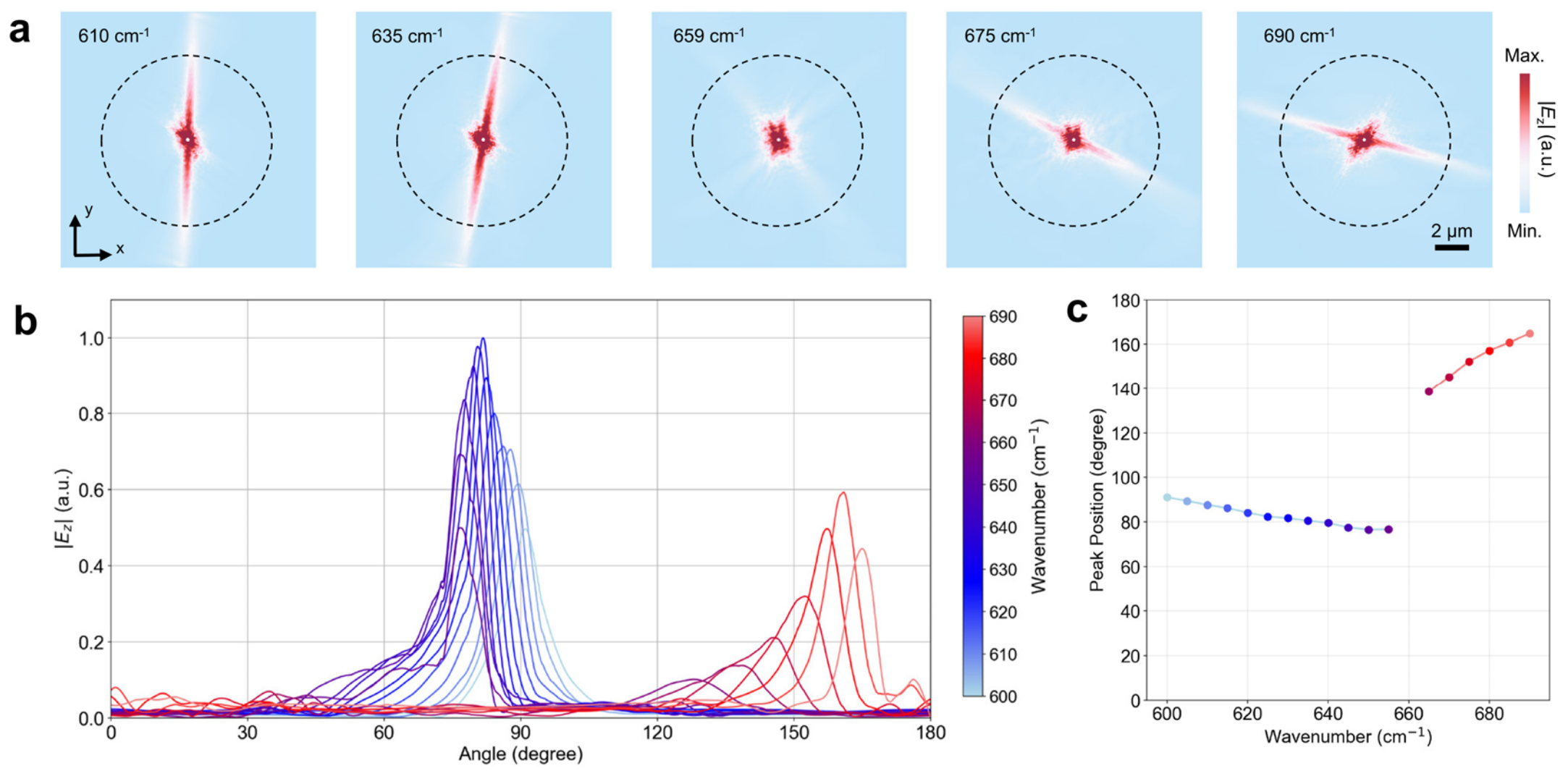

Fig. R15 Quantitative analysis of frequency-dependent rotation of quasi-1D polaritons. a, Electric field component |$\mathbf{E}_z$| at different frequencies generated by a point dipole of a 400 nm-thick α-MoO_3 film / bulk β-Ga_2O_3 heterostructure from simulations. The twist angle is 26°. **b**, Simulation profiles along circular cuts at 5 μm far from the dipole source (dashed line in panel **a**), with colors denoting cases of distinct wavenumbers. **c**, The extracted peak positions in **b** of different wavenumbers (except for the case 659 cm^{-1}) shows opposite rotation directions on both sides of 659 cm^{-1}.

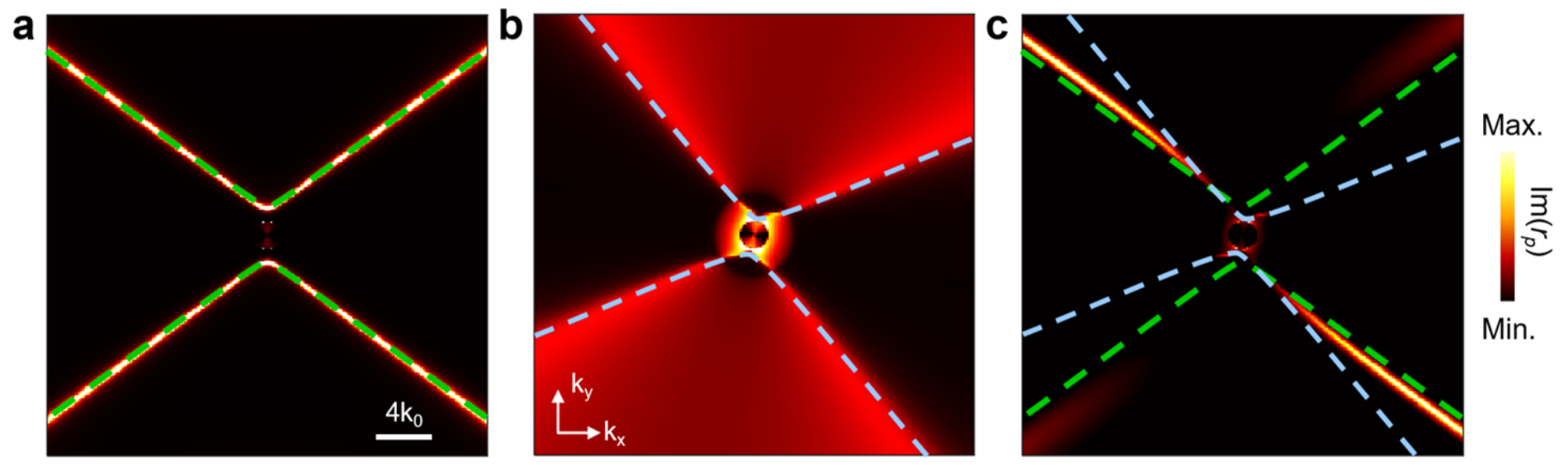

Fig. R16 Angular comparison between the quasi-1D polariton and the α-MoO_3 M_0 mode. IFCs of 200 nm-thick α-MoO_3 film (a), bulk β-Ga_2O_3 (b) and their heterostructure (c) of 640 cm^{-1}. The twist angle is 0°. The fundamental modes (M_0, l = 0) of α-MoO_3 (green) and bulk β-Ga_2O_3 (light blue) are marked with dotted lines, respectively.

Changes made in the revised manuscript and supplementary information:

We thank the reviewer for this question, in response to which we have made the following changes in lines 133 on Page 5 of the revised manuscript:

"as the frequency increases from 600 cm^{-1} to 659 cm^{-1}, the mode rotates clockwise;

beyond 659 cm^{-1}, it rotates counterclockwise up to 690 cm^{-1} (Fig. S9)."

We have also included Fig. R15 as Fig. S9 in the revised supplementary information, as marked in blue:

"Fig. S9 Quantitative analysis of frequency-dependent rotation of quasi-1D polaritons.

a, Electric field component $|\mathbf{E}_z|$ at different frequencies generated by a point dipole of a 400 nm-thick α-MoO_3 film / bulk β-Ga_2O_3 heterostructure from simulations. The twist angle is 26°. **b**, Simulation profiles along circular cuts at 5 μm far from the dipole source (dashed line in panel **a**), with colors denoting cases of distinct wavenumbers. **c**, The extracted peak positions in **b** of different wavenumbers (except for the case of 659 cm^{-1}) shows opposite rotation directions on both sides of 659 cm^{-1}."

3. What is the pruning efficiency of the leakage mechanism—can specific hyperbolic branches be completely suppressed, and under what physical conditions? Additionally, what is the polariton propagation behavior exactly at the critical frequency of 659 cm^{-1}, where the topological transition occurs? Does the quasi-1D character persist at this singular point, or does the mode exhibit fundamentally different behavior?

Reply: Thank you for the reviewer's in-depth questions regarding pruning efficiency and the critical behavior associated with the topological transition. We address these points in detail below.

(1) Conditions for enhance pruning efficiency:

To evaluate the pruning efficiency, we perform an integral of the angular distribution intensity I(θ) in *k*-space and compare the integrals of the quasi-1D polaritons (the brightest angular sector) and the eliminated branch (the second brightest angular sector) to get a figure of merit (FOM) for the measurement of pruning efficiency (Ref. *Nat. Commun.* 2023, 14, 5240). Specifically, we first define the angular distribution intensity I(θ). The total intensity within a small angular sector centered at θ with an angular width Δθ = 0.25° is expressed as

$$I(\theta)= \int_{\theta-\Delta\theta/2}^{\theta+\Delta\theta/2} \int_{1.5k_0}^{10k_0} I\left(r,\theta^{'}\right) r\ dr\, d\theta^{'}.$$

Accordingly, the figure of merit (FOM) for pruning efficiency is defined as $1-I(\theta_2)/I(\theta_1)$ where $I(\theta_1)$ and $I(\theta_2)$ denote the largest and second-largest values of $I(\theta)$, respectively. Fig. R17 shows the influence of the substrate's intrinsic loss on the pruning efficiency. The loss factor is obtained by multiplying the imaginary part of the dielectric function in each direction of β-Ga_2O_3 by the corresponding coefficient simultaneously. The closer the FOM is to 100%, the more effective the suppression becomes.

Given the inherent inevitability of material attenuation, complete cancellation in a strict quantitative sense remains practically unattainable. However, from an experimental standpoint, the limited signal-to-noise ratio of near-field imaging means that once the cancellation branch is sufficiently suppressed, it becomes unobservable, as evidenced by the near-field images presented in this work. Moreover, the FOM analysis presented herein provides a clear pathway for further enhancing the pruning efficiency with two conditions. As shown in the Fig. R17c, reducing the intrinsic loss of β-Ga_2O_3 to 50% of its original value increases the FOM from a baseline of 70% to 80%, and further reducing it to 10% boosts the FOM to 90%. In brief, pruning efficiency can be enhanced by two conditions: First, both the film and substrate must possess sufficiently low intrinsic losses (i.e., low phonon absorption) to ensure efficient coupling. Second, the substrate should be sufficiently thick to prevent back-reflection of leaked energy from its bottom surface. The mode analysis in Fig. R18 also supports this view.

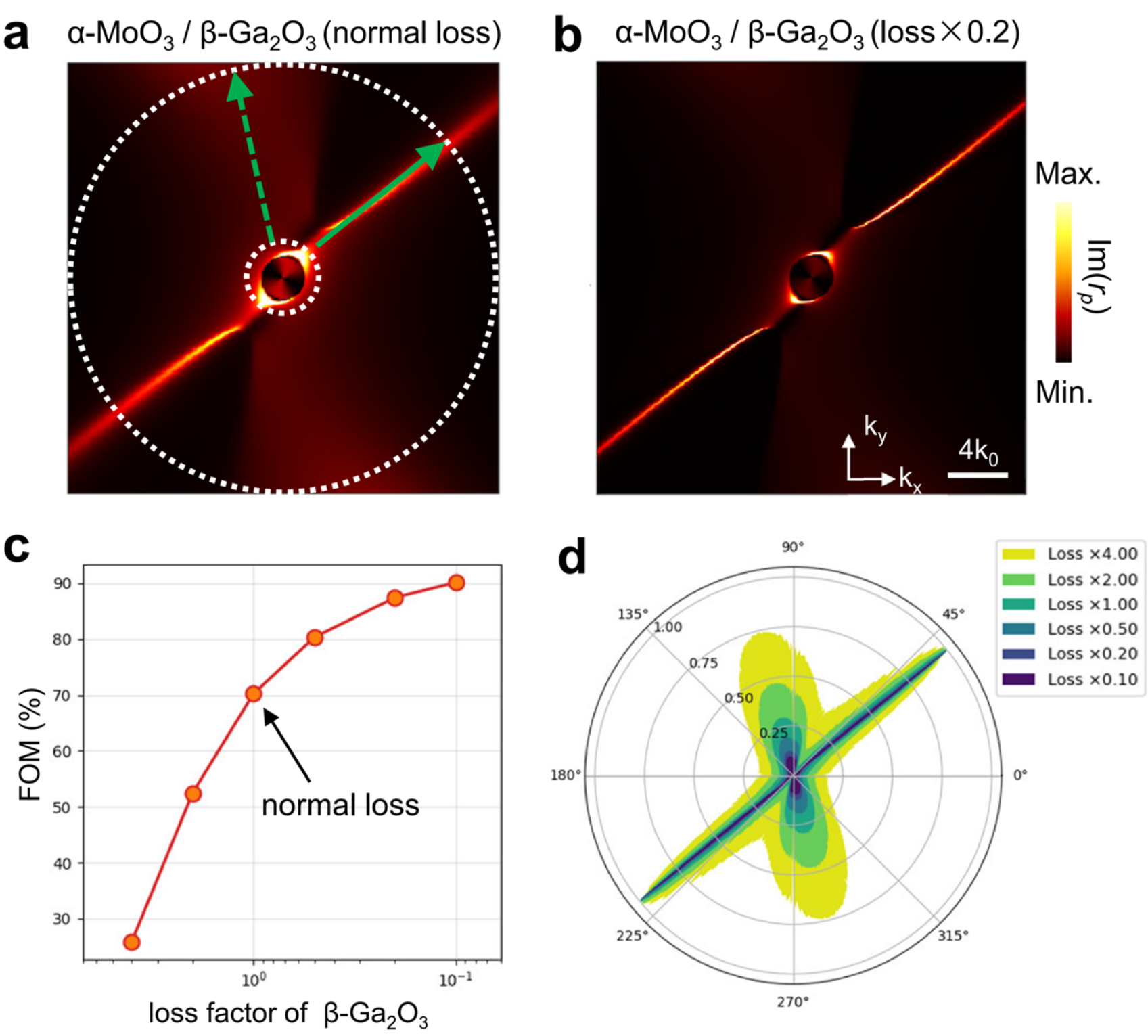

Fig. R17 The influence of the substrate's intrinsic loss on the pruning efficiency. a, b, IFCs of heterostructure of 200 nm-thick α-MoO_3 / bulk β-Ga_2O_3 with normal loss (**a**) and with 0.2 times of loss (**b**). **c**, FOM of pruning efficiency as a function of the loss factor in logarithmic scale. The direction of the largest (the second largest) value of I(θ) is indicated by a green solid (dashed) arrow in **a**. The loss factor of 1 indicates the normal condition. **d**, Polar overlay of the normalized angular integral profiles for all six loss multipliers. Each curve is individually normalized to its maximum.

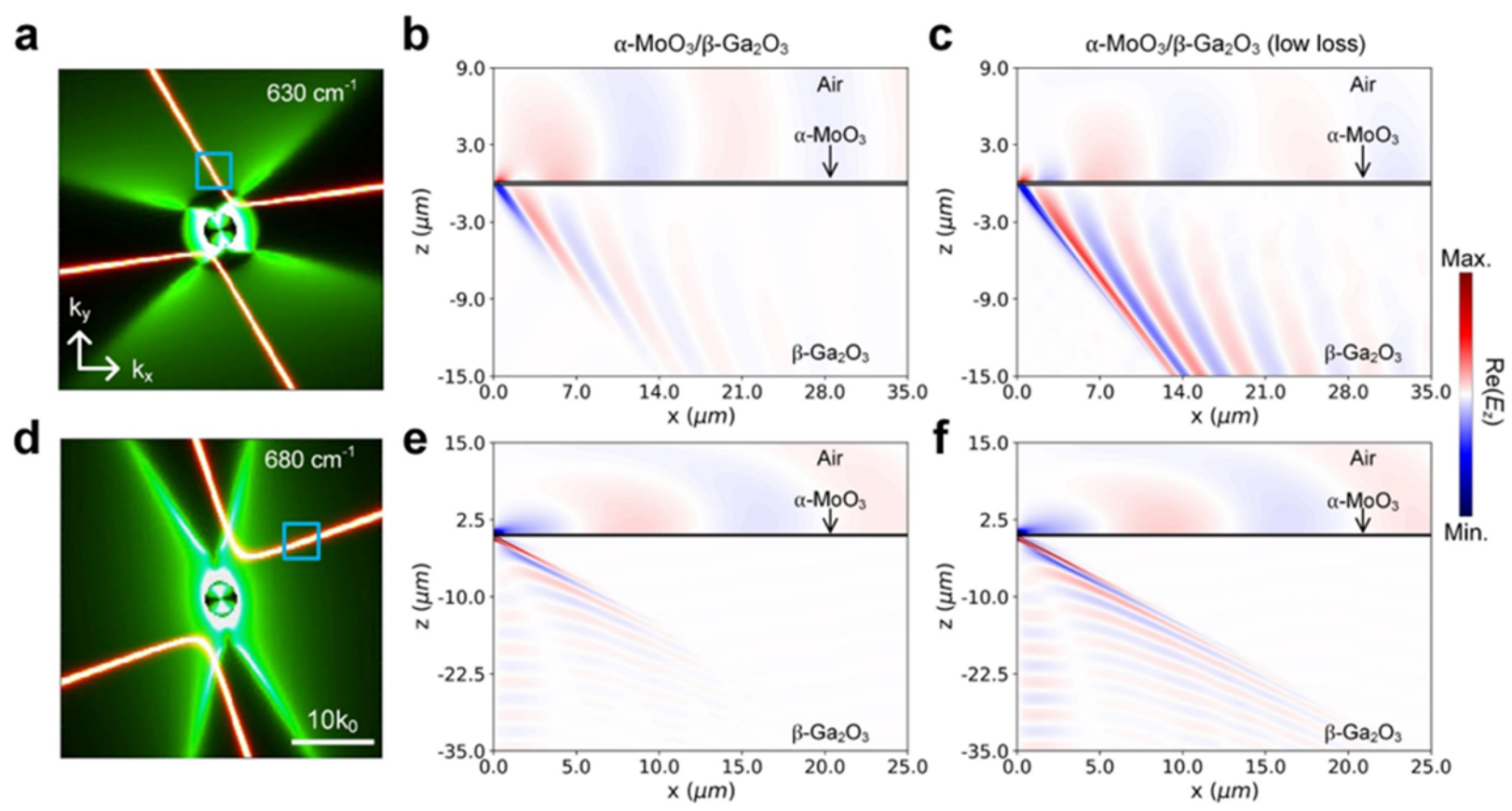

Fig. R18 Mode analysis of leakage directions at 630 cm^{-1} (a-c) and 680 cm^{-1} (d-f)

a, d, Isofrequency contours of β-Ga_2O_3 bulk (green) and 200-nm thick α-MoO_3 flake (orange) at 630 cm^{-1} (**a**) 680 cm^{-1} (**d**). **b, e**, The real part of z-component of the electric field of the heterostructure with normal-loss β-Ga_2O_3 substrate (ε_x=1.6542+0.19985i, ε_y=-5.4726+0.95184i,

ε_z=6.15+0.33194i, ε_{xy}=1.9445-0.19517i for 630 cm^{-1} and 8.5965+0.33336i, ε_y=-1.1628+0.30793i, ε_z=-9.6817+0.74983i, ε_{xy}=1.6747-0.027947i for 680 cm^{-1}). **c, f**, The real part of z-component of the electric field of the heterostructure with low-loss β-Ga_2O_3 substrate (ε_x=1.6542+0.019985i, ε_y=-5.4726+0.095184i, ε_z=6.15+0.033194i, ε_{xy}=1.9445-0.19517i for 630 cm^{-1} and ε_x= 8.5965+0.033336i, ε_y=-1.1628+0.030793i, ε_z=-9.6817+0.074983i, ε_{xy}=1.6747-0.027947i for 680 cm^{-1}). The analysis location of panel **b, c** (**e, f**) is indicated by the blue box in **a** (**d**).

(2) Polariton behavior at the transition frequency (659 cm^{-1}):

At the transition frequency of 659 cm^{-1}, the substrate undergoes strong intrinsic absorption due to out-of-plane phonon resonance. Under such conditions, the substrate ceases to function as an angle-selective leakage channel and instead acts as a lossy medium that imposes uniform damping on the intrinsic hyperbolic modes of the film. As a result, quasi-1D propagation characteristics vanish at this critical frequency, and the polariton modes in the heterostructure return to conventional, highly damped hyperbolic polaritons without preferred orientation (Fig. R19).

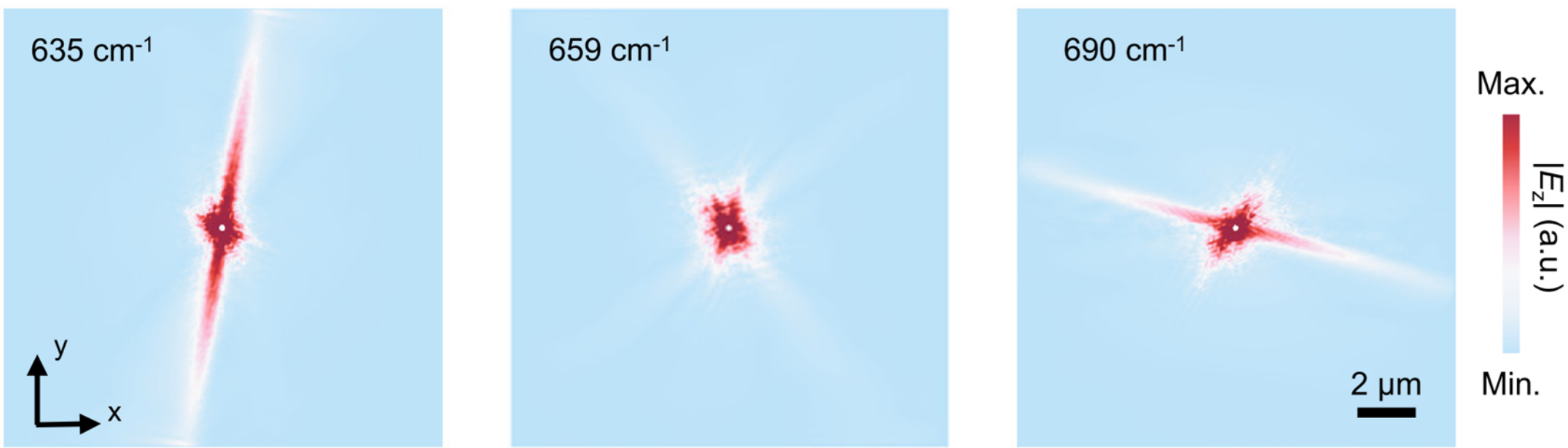

Fig. R19 Electric field component |**E**$_z$| at different frequencies generated by a point dipole of a 400 nm-thick α-MoO_3 film / bulk β-Ga_2O_3 heterostructure from simulations. The twist angle is 26°.

Changes made in the revised manuscript and supplementary information:

We thank the reviewer for this question, in response to which we have made the following changes in the revised manuscript, as marked in blue:

In lines 178-179 on Page 6:

"If the intrinsic loss of β-Ga_2O_3 substrate can be reduced, the pruning efficiency can be improved (Supplementary Note 9 and Fig. S13) ......"

In line 135 on Page 5:

"There's a discontinuous jump at the transition frequency of 659 cm^{-1}, where the polaritons exhibit a linear crossing-like dispersion characteristic (Fig. S9a). "

We have also added Note 9, included Fig. R17 as Fig. S13 and Fig. R19 as part of Fig. S9a in the revised supplementary information, as marked in blue:

"Note 9: Analysis of the pruning efficiency

To evaluate the pruning efficiency, we perform an integral of the angular distribution intensity I(θ) in *k*-space and compare the integrals of the quasi-1D polaritons (the brightest angular sector) and the eliminated branch (the second brightest angular sector) to get a figure of merit (FOM) for the measurement of pruning efficiency [11]. Specifically, we first define the angular distribution intensity I(θ). The total intensity within a small angular sector centered at θ with an angular width Δθ = 0.25° is expressed as

$$I(\theta)= \int_{\theta-\Delta\theta/2}^{\theta+\Delta\theta/2} \int_{1.5k_0}^{10k_0} I(r,\theta')r\ dr\, d\theta'.$$

Accordingly, the figure of merit (FOM) for pruning efficiency is defined as $1-I(\theta_2)/I(\theta_1)$ where $I(\theta_1)$ and $I(\theta_2)$ denote the largest and second-largest values of I(θ), respectively. Fig. S13 shows the influence of the substrate's intrinsic loss on the pruning efficiency. The loss factor is obtained by multiplying the imaginary part of the dielectric function in each direction of β-Ga_2O_3 by the corresponding coefficient simultaneously. The closer the FOM is to 100%, the more effective the suppression becomes. As shown in the Fig. S13c, reducing the intrinsic loss of β-Ga_2O_3 to 50% of its original value increases the FOM from a baseline of 70% to 80%, and further reducing it to 10% boosts the FOM to 90%. In brief, pruning efficiency can be enhanced by reducing intrinsic losses (i.e., low phonon absorption) to ensure efficient coupling."

"Fig. S13 The calculation of pruning efficiency.

a, b, IFCs of heterostructure of 200 nm-thick α-MoO_3 / bulk β-Ga_2O_3 with normal loss (**a**) and with 0.2 times of loss (**b**). **c**, FOM of pruning efficiency as a function of the loss factor in logarithmic scale. The direction of the largest (the second largest) value of I(θ) is indicated by a green solid (dashed) arrow in **a**. The loss factor of 1 indicates the

normal condition. **d**, Polar overlay of the normalized angular integral profiles for all six loss multipliers. Each curve is individually normalized to its maximum."

4. The near-field images at step boundaries (Fig. 3d and Fig. S15) show relatively low signal-to-noise ratio, which may affect the reliability of the claimed zero-reflection observation. The authors should discuss the origin of this noise and whether it limits the angular or frequency range over which zero reflection can be confidently asserted. Additionally, I wonder whether the effect remains equally valid when polaritons propagate from the thicker region to the thinner region compared to the opposite direction, or if asymmetry in the step geometry introduces directional dependence.

Reply: Thanks for the Reviewer's valuable suggestions. The low signal-to-noise ratio is a result of the environmental noise and the interference of the far-field signal, which indeed weakened the robustness of our claim on zero-reflection. We have improved our experimental conditions and retested the samples. New results with higher signal-to-noise ratio are given in the first column of the Fig. R20, with the simulation results shown on the right side. To further demonstrate the ultra-broad-angle zero-reflection phenomenon, we redesigned the experimental setup by implementing a gold reflective structure (Fig. R8), which provides a more robust and well-defined reflective boundary compared to a physical step edge, as mentioned above. We have updated Fig. 3 in the revised manuscript with the experimental data shown in Fig. R8

Additionally, the zero-reflection effect still works when polaritons propagate from the thicker region to the thinner region. The simulation results are given Fig. R21. The step height is 60 nm, the gold disk is placed on the thicker area. When the polariton propagates across the step boundary, there is no reflection observed.

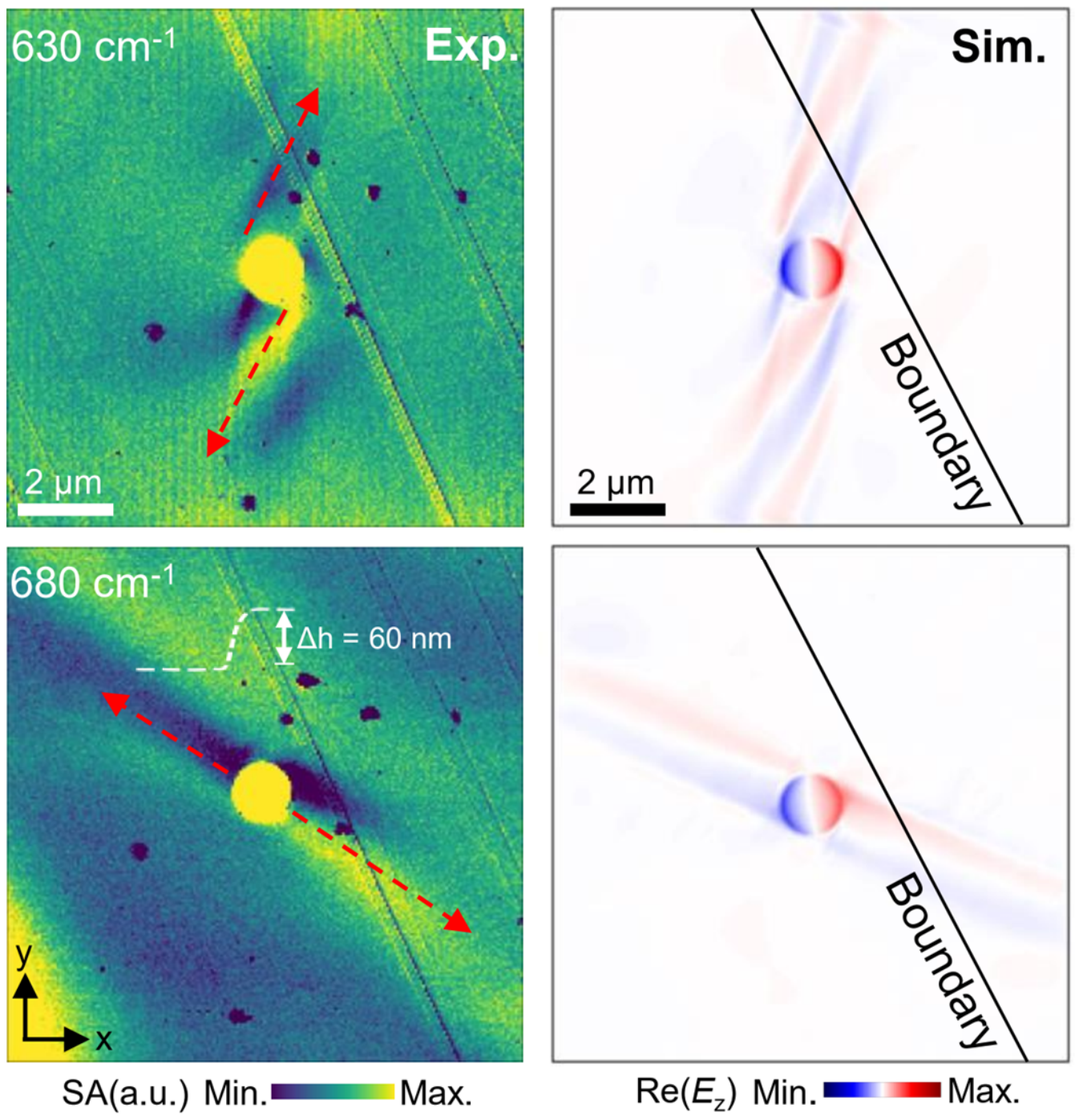

Fig. R20 The newly measured results of the zero-reflection sample and the corresponding simulation results.

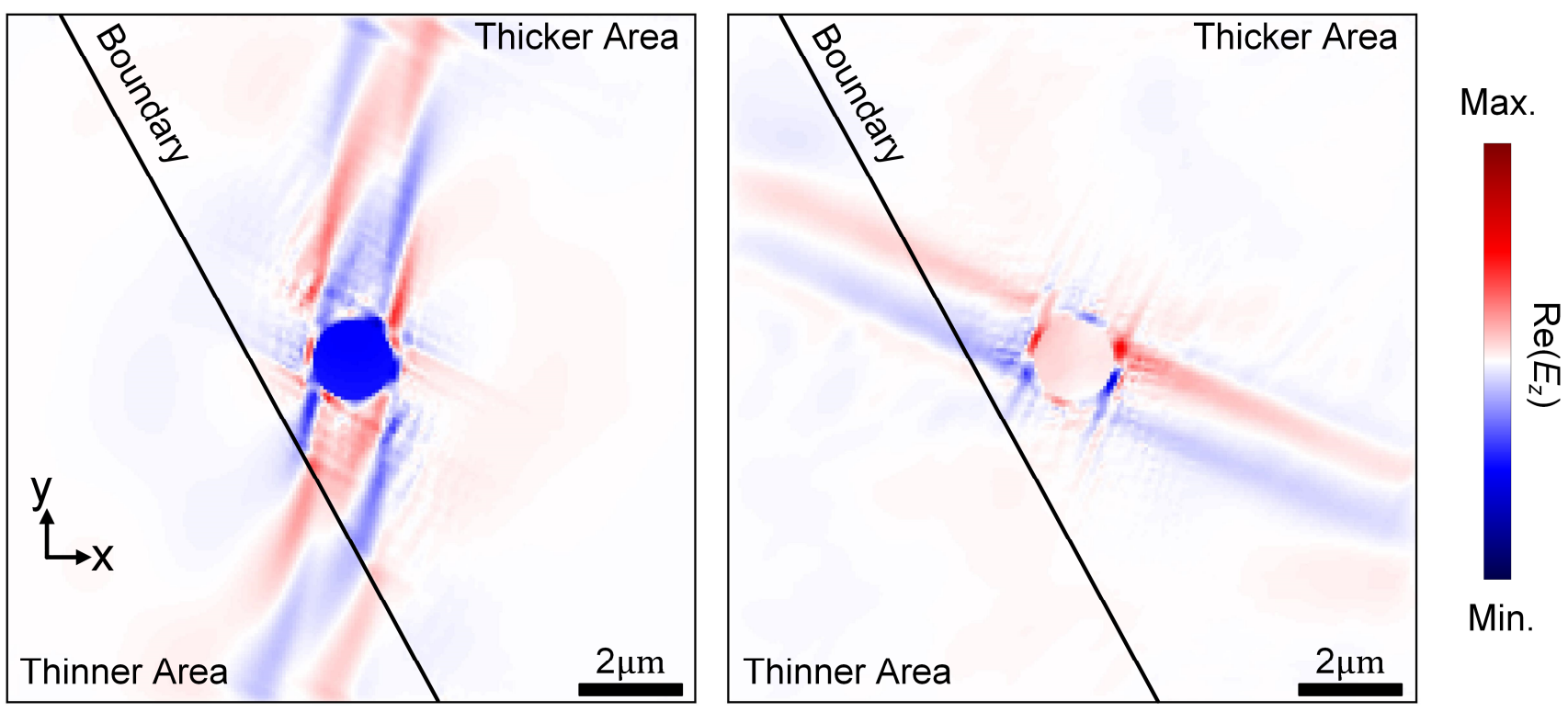

Fig. R21 The simulation results of quasi-1D polariton propagating across the boundary from thicker area to thinner area.

Changes made in the revised manuscript and supplementary information:

We thank the reviewer for this question, in response to which we have updated Fig. 3 with the experimental data in Fig. R8 in the revised manuscript and made the following changes in the revised manuscript, as marked in blue:

In lines 201-205 in Page 7:

"A single-crystal gold flake is placed on top of the heterostructure. Due to its high reflectivity, the edge of the gold flake serves as a mirror to facilitate the observation of zero-reflection phenomena. A gold disk is placed on top of the heterostructure to excite quasi-1D polaritons which propagates towards the gold edge. The microscope image of the fabricated sample is shown in Fig. 3b. "

In lines 225-235 in Page 8:

" The wideband zero reflection is verified in experiment with s-SNOM using the sample in Fig. 3b. The experimental results are shown in the left panels of Fig. 3e and f. Cases of 630 and 680 cm^{-1} are presented for demonstration. Simulation results are performed using the measured geometry parameters and correspond well with the experiment results, shown in the right panels of Fig. 3e and f. No reflection is observed in the measured near field signal and simulation, and it should be noted that when quasi-1D polaritons arrive at the gold edge, its energy flow transmits into the α-MoO_3 layer beneath the gold flake, which cannot be observed in the measured near-field image. For comparison, the reflection of hyperbolic polariton at the gold mirror is given in Fig. S22. The zero-reflection phenomenon of quasi-1D polariton is not limited to gold-mirror interfaces but is also observed at the natural boundaries of α-MoO_3 (Fig. S12b)."

In lines 501-515 on Page 18:

Text before revision:

"**a**, Schematic of wide-band zero-reflection of quasi-1D polariton. Two bricks with different colors represent different thickness. Quasi-1D polaritons with different frequencies excited by input light through a gold disk propagates cross the step without reflection. **b**, The theoretical analysis in momentum space of zero reflection of quasi-1D polariton at the boundary. The blue solid and dashed arrows show the incident wave vector and corresponding Poynting vector. The gray solid and dashed vectors show the reflective wave vector and Poynting vector, which are forbidden by the IFC. **c**, The simplified real space schematic of zero-reflection transmission corresponding to the case in **b**. Subscripts “i”, “r” and “t” stand for incidence, reflection and transmission. **d**, The experimental and simulation results of zero-reflection in α-MoO_3/β-Ga_2O_3, with a step height of 60 nm, measured with AFM (white dashed line). The thickness of the

thinner α-MoO_3 is about 120 nm. The twist angle is 26°. The presented frequencies are 630 cm^{-1} and 680 cm^{-1}. The quasi-1D polariton crosses the boundary with no reflection, which is verified by the simulation results on the right side."

Text after revision:

" **a,** Schematic of wide-band zero-reflection of quasi-1D polaritons whose frequencies are indicated by different colors. **b,** Microscopic image of the measured sample. The twist angle is 26°. A single crystal gold plate is used as a mirror. The thickness of the α-MoO_3 is 120 nm. The scale bar is 5 μm. **c**, The theoretical analysis of zero reflection. The blue solid and dashed arrows show the incident wave vector and corresponding Poynting vector. The gray solid and dashed vectors show the reflective wave vector and Poynting vector that would occur if it was a hyperbolic situation (brown dashed curve), which are eliminated by the leakage mechanism. Subscripts "i" and "r" stand for incidence and reflection. The scale bar is $2k_0$. **d**, Simplified real space schematic of zero-reflection of the case in **c**. **e-f**, The experimental (left) and simulation (right) results of zero-reflection of quasi-1D polaritons of the heterostructure (measured in the red dashed box in b) for excitation frequency of 630 cm^{-1} (**e**) and 680 cm^{-1} (**f**). The quasi-1D polaritons propagate towards the boundary with no reflection, which is verified by the simulation results. The scale bar is 2 μm."

5. The near-field image in Fig. 4b (α-MoO_3/$CdWO_4$ heterostructure) exhibits noticeably lower signal-to-noise ratio compared to Fig. 2b, particularly in the right panel where the propagation pattern appears more characteristic of hyperbolic spreading rather than well-confined quasi-1D rays. The authors should explain the reason.

Reply: Thanks for Reviewer's valuable suggestion. We have carefully identified the root cause of this discrepancy: in the previous manuscript, the twist angle and top-layer thickness of the α-MoO_3/$CdWO_4$ heterostructure were not optimally configured, resulting in suboptimal leakage trimming efficiency. This, in turn, compromised the confinement of the quasi-1D polaritons, manifesting as a degraded signal-to-noise ratio and a propagation mode characteristic of hyperbolic diffusion in the near-field images.

To address this issue, we fabricated a new α-MoO_3/$CdWO_4$ sample with geometric

parameters optimized according to our theoretical predictions. The twist angle is changed from -10° to 45° and the thickness of α-MoO_3 is changed from 960 nm to 200 nm. The near-field measurements of this sample are presented in Figure R22 (updated as Figure 4c-e in the revised manuscript). The results show that the optimized quasi-1D polaritons exhibit a propagation length approaching 10 μm, confirming their excellent long-range propagation capability.

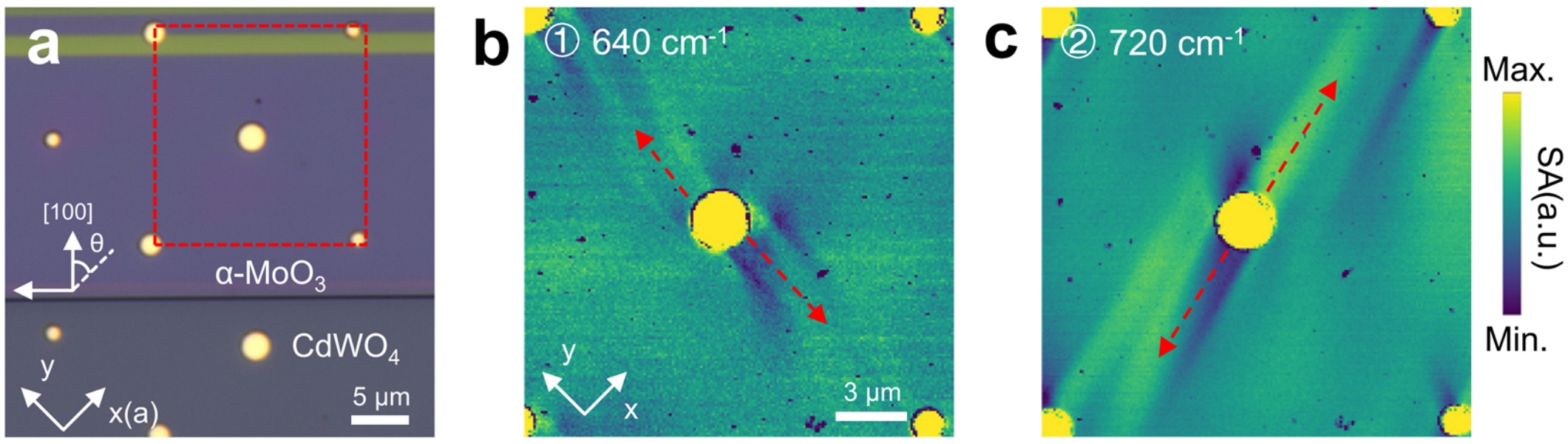

Fig. R22 The near-field images of quasi-1D polaritons in α-MoO_3/$CdWO_4$ heterostructure. a, Microscopic image of the measured 200-nm-thick α-MoO_3 flake/$CdWO_4$ bulk heterostructure, featuring a central gold disk as the excitation source. The twist angle between the [100] axis of these two kinds of material is 45°. **b, c,** Near-field optical amplitude distribution illustrating the quasi-1D polariton of the heterostructure

Changes made in the revised manuscript and supplementary information:

We thank the reviewer for this question, in response to which we have updated Fig. 4 with Fig. R22 in the revised manuscript and made the following changes in the revised manuscript, as marked in blue:

In lines 277-282 in Page 10:

" To verify the generality of our scheme, we used the α-MoO_3/$CdWO_4$ heterostructure as an example to verify the reconfigurable propagation characteristics of quasi-1D polaritons. The microscope image is shown in Fig. 4c. By designing an appropriate twist angle for the heterostructure, a clear ray-like propagation of quasi-1D polaritons is achieved, with frequencies located at 640 cm^{-1} (Fig. 4d), and 720 cm^{-1} (Fig. 4e), respectively."

In lines 530-534 in Page 19:

"**c**, Microscopic image of the measured 200-nm-thick α-MoO_3 flake/$CdWO_4$ bulk heterostructure, featuring a central gold disk as the excitation source. The twist angle θ between the [100] axis of these two kinds of material is 45°. **d, e,** Near-field optical amplitude distribution illustrating the quasi-1D polariton of the heterostructure, corresponding to the cases ①, ② marked in **b**."

Our point-by-point responses to the Reviewers:

We thank all referees and the editor for their careful reviewing of this work and the recommendation for the publication of this work. Please, find our point-by-point reply below.

Reviewer #1 (Comments for the Author):

>> All the previous concerns have been fully addressed and the revised manuscript has improved significantly in terms of both scientific rigor and data presentation. I recommend acceptance now.

Response: We sincerely thank the reviewer for the constructive comments, which have greatly helped improve our manuscript. We also appreciate the reviewer’s recommendation of our work.

Reviewer #2 (Comments for the Author):

>> The authors have addressed most of my technical concerns, and the manuscript has been substantially improved. Therefore, I would recommend the manuscript to be published on Nature Communications.

Response: We are grateful to the reviewer for their constructive feedback on our manuscript, which has greatly helped us improve its quality. We also deeply appreciate the reviewer’s recommendation for our publication in *Nature Communications*.

Best regards

The authors
